# Medication Use by Older Adults with Frailty: A Scoping Review

**DOI:** 10.3390/pharmacy13060170

**Published:** 2025-11-21

**Authors:** Rishabh Sharma, Tanaya Sharma, Brent McCready-Branch, Arshia Chauhan, Caitlin Carter, SooMin Park, Imra Hudani, Prapti Choudhuri, Tejal Patel

**Affiliations:** 1 School of Pharmacy, University of Waterloo, 10 Victoria St S A, Kitchener, ON N2G 1C5, Canada; 2 Schlegel-UW Research Institute for Aging, 250 Laurelwood Dr, Waterloo, ON N2J 0E2, Canada

**Keywords:** older adults, potentially inappropriate medication, frailty, polypharmacy

## Abstract

Frailty among older adults heightens their risk of negative health outcomes, and medication use plays a major role in this increased vulnerability. Various aspects of medication use elevate the risk of poor outcomes in individuals with frailty. The current scoping review was designed to explore medication use in older adults with frailty in primary care, focusing on the prevalence of potentially inappropriate medications (PIMs), polypharmacy, medication adherence, and their role in contributing to adverse drug events. This scoping review was conducted using the Arksey and O’Malley, supplemented by the Preferred Reporting Items for Systematic Reviews and Meta-Analyses (PRISMA) Extension for Scoping Reviews (PRISMA-ScR) guidelines. A search of the literature was conducted from inception to November 2023 in Ovid EMBASE, PubMed (MEDLINE), Scopus, EBSCOhost CINAHL, and Ovid International Pharmaceutical Abstracts. Studies which met the eligibility criteria included older adults with frailty (≥65 years) living at home, defined frailty criteria, and assessment of medication use. Out of the 4726 studies screened, 223 were included, conducted across 39 countries. Frailty prevalence varied widely from 0.9% to 89.2%. Polypharmacy (5–9 medications) and hyper-polypharmacy (≥10 medications) were notably more common among individuals with frailty, with polypharmacy rates ranging from 1.3% to 96.4%. Twelve studies reported PIM prevalence among individuals with varying levels of frailty, ranging from 2.4% to 95.9%. This scoping review highlights the challenges and complexities involved in understanding the relationship between medication use and frailty in older adults.

## 1. Introduction

The global population is aging rapidly. By 2030, one in six people worldwide will be aged 60 or older, from 1 billion in 2020 to 1.4 billion. By 2050, this number is expected to reach 2.1 billion, with those aged 80 years or over tripling to 426 million [1]. In Canada, the aging trend is accelerating as baby boomers move into the 65+ age group. Currently, over 800,000 Canadians are 85 or older, a number projected to triple by 2050 [2]. This demographic shift presents significant implications for healthcare systems, economies, and societies at large. Older adults with comorbidities such as hypertension, diabetes, cardiovascular diseases, and stroke are at a higher risk of morbidity and mortality [3]. Multimorbidity—defined as having two or more chronic conditions—is increasingly common, with 8.3% of Canadians reporting three or more in 2021, up from 7.5% in 2015 [4,5]. There are several challenges that are associated with aging, one of the most significant contributors being the increased risk of frailty [6].

Currently, there is no clear consensus on the definition of frailty. A widely accepted definition, proposed by Fried et al., describes frailty as “a state of age-related physiological vulnerability resulting from impaired homeostatic reserve and a reduced capacity of the organism to withstand stress” [7]. Increasingly, researchers acknowledge the multifactorial nature of frailty [8]. A recent integral conceptual model by Gobbens et al. defines frailty as a dynamic state affecting an individual who experiences losses in one or more domains of human functioning (physical, psychological, and social), caused by various influences, which increases the risk of adverse outcomes [9]. Frailty can be described through various lenses, including physical, psychological, and social aspects [10].

Frailty is commonly classified into three categories: robust, pre-frail, and frail [7]. Individuals may move between these stages depending on various factors. Frailty is often worsened by multiple medication use, poor nutrition, inactivity, and social isolation [11]. Older adults with frailty are more vulnerable to serious health outcomes from minor illnesses or events, such as influenza or falls, and face higher risks of hospitalization, long-term care, and mortality [12]. These issues can significantly affect daily functioning and increase dependence on caregivers, impacting both the individual’s and their family’s quality of life [13]. Several tools are available to assess frailty, helping to guide clinical care and identify those at risk. Common tools include the Frailty Index (FI), which evaluates accumulated health deficits; the Fried Frailty Phenotype, based on physical markers like weight loss and weakness; and the Clinical Frailty Scale (CFS), a visual rating of fitness levels. Other tools include the Geriatric 8 (G8), Edmonton Frail Scale (EFS), Tilburg Frailty Indicator (TFI), and PRISMA-7, each assessing various physical, psychological, or social dimensions [14]. These tools vary in complexity and focus. With the increases in the aging population in Canada, it is important to monitor this population due to its impact on quality of life, ability to age at home, and increased risk of mortality [15,16,17].

Individuals with frailty experience heightened sensitivity to medication, which can increase the chances for adverse effects even at regular dosages [18,19,20]. Furthermore, use of polypharmacy, potentially inappropriate medications (PIMs), medication adherence, and errors impact health outcomes [21]. A systematic review and meta-analysis by Toh et al. reported that among frail older adults (synthesized data from 66 studies, involving 167,888 participants), polypharmacy (≥5 medications) had a pooled prevalence of 59%, and hyper-polypharmacy (≥10 medications) was observed in 22% of cases [19]. Additionally, cognitive impairments may cause difficulties in remembering doses, while physical limitations can make tasks like opening pill bottles challenging, further complicating medication management [22]. Finally, medication use in this population is further complicated by age-related changes such as altered drug metabolism, and reduced renal and liver function, all of which increase the risk of medication toxicity and treatment-related harm [20].

In recent years, there has been an increase in both the number and variety of studies exploring the relationship between medication use and frailty. Researchers are delving into various aspects, such as how specific medications might contribute to or alleviate frailty, the impact of polypharmacy, and the interplay between different drug classes and frailty risk. Despite this expanding body of research, a comprehensive review that synthesizes all these findings and provides a holistic understanding of the concepts is still missing. This scoping review was conducted to explore and summarize the existing research on medication use in community-dwelling older adults with frailty.

## 2. Materials and Methods

To conduct this scoping review, we followed the five-stage framework proposed by Arksey and O’Malley [23], as described below. Additionally, we utilized the Preferred Reporting Items for Systematic Reviews and Meta-Analyses (PRISMA) Extension for Scoping Reviews (PRISMA-ScR) to structure and present our results comprehensively (Supplementary Table S1) [23,24].

### 2.1. Identifying the Research Question

This scoping review aims to comprehensively explore medication use patterns and outcomes among older adults with frailty in primary care settings. The primary objectives are as follows:

- To analyze medication use in older adults with frailty by examining the prevalence of PIMs, polypharmacy, capacity for medication management, adherence to prescribed regimens, medication errors, and associated health outcomes.
- To provide recommendations for addressing medication-related issues in this population by detailing available tools (such as medication review instruments) and processes (including deprescribing strategies) that can aid in identifying and managing these problems.

### 2.2. Identifying the Relevant Studies

A comprehensive search strategy was designed and implemented by an experienced librarian (CC) across five databases—PubMed (MEDLINE); Ovid Embase; Scopus; EBSCOhost CINAHL; and Ovid International Pharmaceutical Abstracts—from inception to 3 November 2023. The core search concepts included in the search strategy were frailty; elderly; and medication use. All database search strategies contained keywords and associated synonyms for each of these core concepts, as well as relevant subject headings, if the database contained an index of controlled vocabulary. Search terms were linked with Boolean operators (AND/OR) and keywords were limited to the title and abstract fields only. Truncation was applied to several keywords and the proximity or adjacency search operator was utilized, based on the database’s functionality. All search strategies were limited to English only, and conference abstracts were removed from the Ovid Embase search results. The complete search strategies for each database can be found in Supplementary Table S2. The search results were exported into Covidence (Veritas Health Innovation, Melbourne, Australia) and duplicates were removed.

### 2.3. Study Selection

Eligibility criteria for study inclusion were developed using the Evidence-Based Medicine PICO framework (Population, Intervention or Exposure, Comparison or Control, Outcomes), focusing on community-dwelling frail older adults (mean or median age ≥ 65 years). Studies were included if they assessed frailty using validated criteria and examined any aspect of medication use, such as polypharmacy, PIMs, medication errors, deprescribing, medication management, or medication-related health outcomes. Eligible study designs included observation (cohort, cross-sectional, case–control) and interventional (randomized-controlled trials (RCTs), pre–post) studies. Articles were excluded if they were not published in English, were expert opinions, case reports, abstracts, literature reviews, and protocols without data analysis, involved non-human participants, or were conducted in institutional settings such as hospitals or long-term care. A two-stage screening process was conducted by a team of seven research assistants (RS, TS, BMB, AC, SP, IH, PC). In the first stage, titles and abstracts were independently reviewed by two independent research assistants. In the second stage, full-text articles were independently reviewed by two independent research assistants, with disagreements resolved through discussion with a third reviewer (TP). Weekly team meetings were held to ensure consistency and resolve discrepancies.

### 2.4. Data Charting

We created a standard Microsoft^®^ Excel^®^ spreadsheet, specifically the Office 365 ProPlus Version 1906 form, in advance to extract essential information from the articles. The following data were abstracted: study design (qualitative/quantitative studies, RCTs, non-RCTs, retrospective studies), location of the study (country and setting such as primary care), primary and secondary objectives, sample size, type and measure of frailty, population demographics (age, gender, number of medications, number of medical conditions, severity of frailty, baseline medication use, etc.), medication use interventions, study outcomes, results, and additional comments. Data abstraction was independently completed by two reviewers from the team of seven research assistants. The extracted data were then compared by one more reviewer from the team of research assistants who had not performed data extraction for the study to ensure accuracy, consistency, and completeness.

### 2.5. Collating, Summarizing, and Reporting the Results

To address the first primary objective, we employed a narrative synthesis approach. We began by developing a preliminary synthesis, grouping studies that focused on similar concepts, such as the prevalence of frailty using different criteria, the prevalence of PIMs, and the prevalence of polypharmacy, into a tabular format. Using Excel, we created tables to explore relationships between medication use, polypharmacy, hyper-polypharmacy, and frailty. Additionally, we reported the results of any type of statistical analyses conducted to investigate the association between medication use and frailty in these studies.

For the second objective, we detailed any interventions or recommendations for addressing medication-related issues in this population. These included descriptions of available tools, such as medication review instruments, and processes, including deprescribing strategies, that can aid in identifying and managing these problems.

## 3. Results

**Figure 1:**
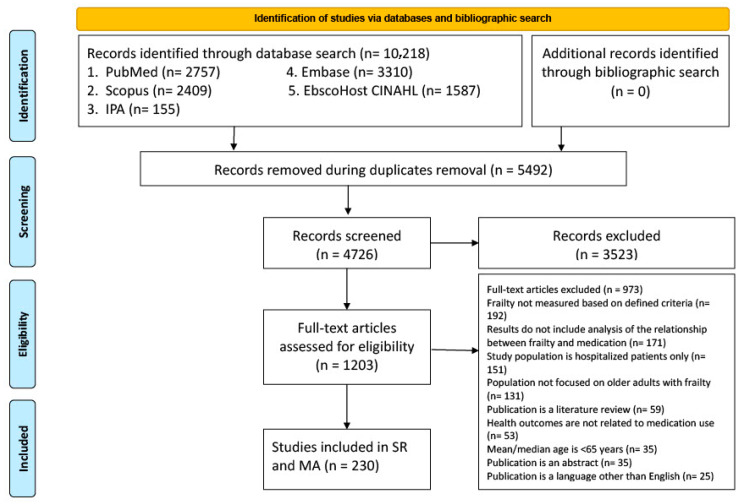
PRISMA flow diagram.

A total of 10,218 references were identified across various electronic databases (see Figure 1, PRISMA Flow diagram). After removing 5492 duplicate entries, 4726 titles and abstracts were screened based on the study’s predefined inclusion and exclusion criteria. Of these, 1203 articles were selected for full-text review, resulting in 230 eligible studies. Out of 230 studies, 223 are unique, indicating that 7 studies are overlapping or use the same population data.

### 3.1. Characteristics of Included Studies

**Figure 2:**
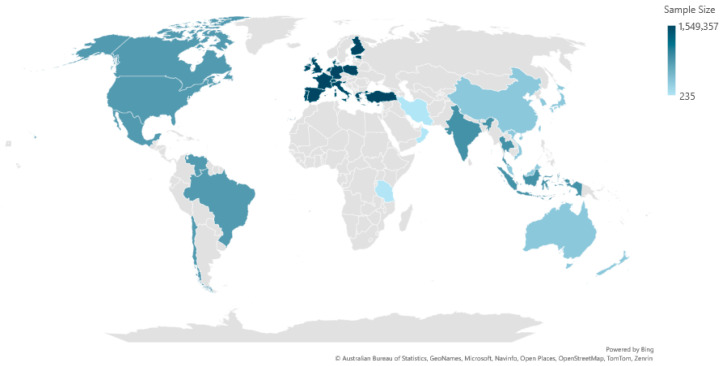
Global distribution of studies collected in this scoping review by region and sample size.

The 223 studies were conducted across 39 countries [25,26,27,28,29,30,31,32,33,34,35,36,37,38,39,40,41,42,43,44,45,46,47,48,49,50,51,52,53,54,55,56,57,58,59,60,61,62,63,64,65,66,67,68,69,70,71,72,73,74,75,76,77,78,79,80,81,82,83,84,85,86,87,88,89,90,91,92,93,94,95,96,97,98,99,100,101,102,103,104,105,106,107,108,109,110,111,112,113,114,115,116,117,118,119,120,121,122,123,124,125,126,127,128,129,130,131,132,133,134,135,136,137,138,139,140,141,142,143,144,145,146,147,148,149,150,151,152,153,154,155,156,157,158,159,160,161,162,163,164,165,166,167,168,169,170,171,172,173,174,175,176,177,178,179,180,181,182,183,184,185,186,187,188,189,190,191,192,193,194,195,196,197,198,199,200,201,202,203,204,205,206,207,208,209,210,211,212,213,214,215,216,217,218,219,220,221,222,223,224,225,226,227,228,229,230,231,232,233,234,235,236,237,238,239,240,241,242,243,244,245,246,247,248,249,250,251,252,253,254]. The characteristics of the included studies are summarized in Supplementary Table S3. The studies have been divided into different regions as follows: 41 studies were conducted in North and South America ([25,31,32,37,45,50,53,57,64,69,71,74,75,77,84,86,90,98,104,108,109,112,115,123,126,132,134,143,162,164,167,170,175,195,196,210,216,223,224,244]), 6 studies in the Middle East ([34,106]), 106 studies in Europe ([26,27,28,29,30,33,35,37,39,41,43,47,51,55,59,60,62,65,66,72,76,78,79,81,85,91,96,99,100,103,105,106,107,111,114,116,117,118,119,120,121,124,125,127,130,131,133,135,136,137,142,146,147,150,151,152,153,154,156,158,159,160,161,165,166,168,173,177,179,180,181,183,185,187,188,190,192,194,197,200,203,204,205,206,208,209,211,213,217,219,225,226,229,233,235,238,239,241,242,243,245,247,250,251,254]), 11 studies in Southeast Asia ([42,128,138,140,145,155,191,199,212,220,246]), 56 studies in the Western Pacific ([36,40,44,46,58,61,63,67,68,80,82,83,87,88,92,95,102,113,122,129,139,144,149,157,163,169,171,172,176,178,186,189,193,198,201,202,207,214,215,218,221,230,231,232,234,236,237,240,248,249,252,253]), 1 study in Africa [89], and 2 studies in Australia and the USA ([70,93]). Figure 2 illustrates the heatmap showing the distribution of studies collected in this scoping review by region and sample size. A total of 202 studies included both women and men ([25,26,27,28,29,30,31,32,33,34,35,36,37,38,39,41,42,44,45,46,47,48,49,50,51,52,53,54,55,56,57,58,59,60,61,62,64,65,66,67,68,69,70,71,72,73,74,75,76,77,78,79,80,81,84,85,86,87,88,89,90,91,92,93,94,95,96,97,98,99,100,101,102,103,104,105,106,107,108,109,110,111,112,113,114,115,116,117,118,119,120,121,122,123,124,125,126,127,128,129,130,131,132,133,134,135,136,137,138,140,141,142,143,144,145,146,147,148,150,151,152,153,154,155,156,157,158,160,161,162,163,165,166,167,168,169,170,171,172,173,174,175,176,177,178,179,180,181,182,183,185,186,187,188,189,190,191,192,193,194,195,196,197,198,199,200,201,202,203,204,205,206,207,208,209,210,211,212,213,215,216,217,219,220,221,224,225,226,227,228,229,230,231,232,233,234,235,236,237,238,239,240,241,242,243,244,245,246,247,248,249,250,251,252,253,254]); however, seven studies have only included female populations ([40,149,164,186,223,224,249]) and ten studies only included male populations ([43,63,82,83,139,159,184,214,218,222]). Among the studies, 120 were cross-sectional studies ([25,27,28,29,30,31,32,34,36,38,41,42,44,45,50,55,58,61,64,65,68,69,71,72,73,75,77,78,81,84,86,89,94,95,99,100,102,105,107,109,110,111,112,113,115,123,124,125,128,130,131,133,138,139,140,142,143,144,145,148,149,151,154,155,158,160,161,162,167,171,173,175,177,179,180,182,185,187,189,192,193,196,197,198,199,200,201,203,204,206,208,212,213,214,215,216,217,218,219,221,224,225,228,229,231,232,233,234,239,240,242,243,244,247,248,250,251,252,253]), 31 studies were prospective cohort studies, ([40,46,51,57,63,66,67,82,83,85,91,121,132,134,156,157,164,170,178,184,186,188,202,205,207,211,220,222,226,230,236,245]), 18 studies were secondary analyses of a cohort study ([25,33,37,47,63,76,80,88,116,118,126,135,144,152,159,163,183,190,191,223,237]), 6 studies were randomized controlled trials ([70,98,127,150,166,172]), 5 studies were secondary analyses of a trial, ([79,92,93,106,117]), 13 studies were longitudinal studies ([26,59,74,101,104,122,129,136,147,169,181,210,246]), 20 studies were retrospective cohort studies ([39,43,53,60,62,87,114,120,153,165,168,176,194,195,209,227,235,238,241,249]), 3 studies were case–control studies ([56,137,146]), and 1 study was a quasi-experimental uncontrolled pre–post [96]; one each was a randomized, unblinded noninferiority trial [103], repeated cross-sectional cohort study [254], quasi-experimental pre–post test design [90], retrospective pre–post study [108], cluster randomized trial [35], and pilot study [119].

Notably, Ye et al., 2022 [26] published one more study using the same population. Similarly, Thiruchelvam, 2021, authored four studies utilizing data from the Australian Longitudinal Study on Women’s Health (ALSWH), focusing on various aspects such as the prevalence and association of continuous polypharmacy and frailty among older women, frailty and PIMs using the 2019 Beers Criteria, and the impact of home medicines review on frailty among community-dwelling older women ([40,49,97,141]). Additionally, Muhlack et al. published two studies using the same population data [51,52].

### 3.2. Prevalence of Frailty

The analysis of the prevalence of individuals with frailty across different countries reveals significant variability. The prevalence of individuals identified as non-frail, pre-frail, and frail ranged from 7.5 to 93.2% ([75,96]), 11.8 to 72.9% ([125,225]), and 0.9 to 89.2% ([76,99]), respectively. The prevalence of frailty among older adults varies within and across different countries. For example, in Taiwan, frailty prevalence ranged from 2.9% to 45.8%, ([73,189]), while in Brazil, it ranged from 9.4% to 67.4% ([64,109]). Similar variations were found in countries in Europe and North America (Netherlands: 11.6% to 55% ([200,250]); Spain: 3.7% to 89.2% ([99,136]); UK: 9.2% to 51.5% ([43,120]); USA: 6.8% to 41% ([75,104]); Canada: 1.8% to 44% ([57,216])) and in Asia (Turkey: 7.1% to 45% ([81,187]); Singapore: 6.2% to 27% ([145,220]. The highest prevalence of frailty was reported in a cross-sectional study in Spain, with 89.2% (74 out of 83 participants) [99]. 

A total of 40 validated scales to measure frailty were used in studies included in this scoping review. The most used scales were the Fried Frailty Phenotype ([29,31,33,43,44,45,47,51,55,56,63,64,70,71,73,75,76,77,81,82,83,84,85,93,94,95,98,105,109,110,116,117,122,123,126,127,132,134,136,139,149,162,163,164,166,174,178,179,186,188,192,193,196,198,203,204,206,208,209,211,212,217,218,219,223,225,227,230,233,239,244,245,246,247,250,251] and the 5-item FRAIL Scale [28,40,42,88,112,133,142,148,156,157,170,184,191,213,220,234,242]). Few studies used a combination of two or more frailty criteria. A total of 77 studies utilized the Fried Frailty Phenotype, with the frailty prevalence ranging from 0.9% to 67.4% ([76,109]). The prevalence for the 5-item FRAIL Scale ranged from 6.2% to 30.8% ([112,220]). The prevalence for the Edmonton Frailty Scale ranged from 25 to 78% ([79,155]).

### 3.3. Medication Use in Frailty

Twenty studies reported the mean number of medications used by individuals with different frailty statuses, while eight studies reported the median number of medications used by individuals with different frailty statuses, and one study reported both the mean and median numbers of medications used. Supplementary Table S4 shows the distribution of studies reporting the mean or median number of medications in older adults with different frailty statuses. The mean number of medications among older adults with pre-frailty ranges from 2.9 (±standard deviation (SD) 2.2) [234] to 9.9 (±SD 3.7) [215]. For those with frailty, the mean number of medications ranges from 4.3 (±SD 2.9) [234] to 15.6 (±SD 16.8) [173]. Across the studies reporting the median number of medications, among older adults with pre-frailty it ranged from 3 (Interquartile range (IQR) 4) [36] to 12 (range 9–16) [50]. For those with frailty, it ranged from 3 (IQR 3.5) [36] to 16 (range 12–20) [50]. Of the twenty-nine studies that reported on mean and median numbers of medications, nineteen used statistical tests to find out if there is a statistically significant difference in the mean or median number of medications among the three groups of frailty (non-frail, pre-frail, and frail). Seventeen of the nineteen studies that used statistical tests yielded statistically significant results (see Table S4 in Supplementary).

Supplementary Table S5 summarizes the findings from eleven studies on medication usage among different categories of frailty: non-frail, pre-frail, and frail. The most reported medication class among older adults with frailty was for hypertension, with five studies identifying it as the most common medication among participants. Analgesics were reported as the most common medication class in two studies, while medications for the treatment of diabetes was the most prescribed in two studies. Finally, one study found that medications for osteoporosis were the most common among individuals with frailty. The study by Chaitoff et al. [32] reports that among individuals with frailty, the usage rates for diabetes medication, hypertension medication, statins, and aspirin are 33.8%, 75.3%, 45.5%, and 54.9%, respectively. In a study by Ballew et al. [75], 75.7% of individuals identified as non-frail were on a hypertension medication and 52.9% on statins, while a higher proportion of individuals with frailty were on each (87.4% and 54.8%, respectively). Additionally, the study by Jankowska-Polańska et al. [78] highlights that angiotensin-converting enzyme inhibitors are commonly used, with 48% of individuals identified as non-frail and 42.9% of individuals with frailty taking them. The study by Ribeiro et al. [115] shows high usage rates of angiotensin-converting enzyme inhibitors/angiotensin receptor blockers (ACEI/ARBs) and beta-blockers among individuals with frailty, ranging from 83% to 100%. A study by Liu et al. [178] reveals metformin and glucosidase inhibitors as commonly used diabetes medications among individuals with frailty, with usage rates of 39% and 48.8%, respectively. Finally, studies by Koponen et al. [208] and Chen et al. [230] indicate significant usage of analgesics and hypnotics among individuals identified as frail, with rates for analgesics reaching up to 68.1% and for hypnotics up to 19.3%.

### 3.4. Prevalence of Polypharmacy and Hyper-Polypharmacy

Thirty-three studies investigated the prevalence of polypharmacy across three groups of participants that differed in frailty status (Supplementary Table S6). The prevalence of polypharmacy in older adults with frailty ranges from 1.3% to 96.4% ([76,177]). In a study by König et al. [76], polypharmacy was defined as the use of five or more medications per day, with 1.3% of frail individuals affected, compared to 40.7% of pre-frail individuals. In contrast, Krawariti et al., using a definition of four or more medications per day, found a much higher prevalence of 96.4% among frail individuals in a small Greek sample of 53 community-dwelling older adults [177]. Another German study by Buttery et al. [152] observed that 85.8% of frail individuals were on polypharmacy (≥5 medications), with differences across non-frail, pre-frail, and frail populations.

Eleven studies investigated the prevalence of polypharmacy and hyper-polypharmacy across groups of participants identified as non-frail, pre-frail, and frail (Supplementary Table S7). Among these studies, four ([105,124,208,217]) studies found polypharmacy to be most prevalent among groups with pre-frailty. However, in six studies, polypharmacy was found to be more prevalent among the frailty group ([47,70,83,182,204,211]). In contrast, six ([83,105,120,204,211]) found hyper-polypharmacy to be most prevalent among groups with frailty.

The studies reviewed consistently show that polypharmacy (5–9 medications) and hyper-polypharmacy (≥10 medications) are more prevalent among individuals with pre-frailty and frailty compared to individuals identified as non-frail, as summarized in Supplementary Table S7. Polypharmacy rates among individuals identified as non-frail ranged from 16.2% [124] to 49.1% [182]. For individuals with pre-frailty, the prevalence ranged from 39.1% [208] to 74.2% [182]. Polypharmacy rates among the frail individuals ranged from 39.8% [124] to 76% [182].

### 3.5. Prevalence of PIMs in Older Adults Based on Frailty

Twelve studies investigated the prevalence of PIMs across groups ranging in frailty status, each using one or more PIMs criteria ([25,33,40,45,49,52,58,70,71,90,179,184,211]). Five studies used Beers Criteria ([25,40,45,70,184]), three studies used STOPP/START (Screening Tool to Alert doctors to Right Treatment) Criteria ([33,58,211]), three studies ([52,90,179]) used a combination of Beers Criteria and STOPP criteria, and one study used each of the European Union (EU) (7)-PIM List [52], PRISCUS List [52], the Turkish Inappropriate Medication Use in the Elderly (TIME) [179], and Brazilian Consensus Potentially Inappropriate Drugs for Older People [71]. Generally, across all studies that reported on PIMs, the prevalence of PIM among individuals with frailty ranged from 2.4% [211] to 95.9% [40]. Across studies that reported PIMs based on Beers Criteria, the prevalence among individuals with frailty ranged from 13.9% [184] to 84.7% [49]. In contrast, for studies that reported PIMs based on STOPP/START Criteria, the prevalence ranged from 2.4% [211] to 86.7% [33] (refer to Table 1).

### 3.6. Drug-Related Problems in Older Adults with Frailty

Six studies investigated the prevalence of drug-related problems (DRPs) across groups ranging in frailty status (refer to Table 2). Two studies used the Medication Risk Questionnaire (MRQ-10) ([26,161]), two studies used anticholinergic burden (ACB) ([55,160]), one study used Short Emergency Geriatric Assessment (SEGA) [130], and one study used the Swedish Finnish Interaction X-reference (SFINX) Monitor [184]. Generally, across all these studies, the prevalence of DRPs among individuals with frailty ranged from 0.2% [160] to 77.6% [160]. Rhalimi et al. [130] used the SEGA to categorize DRPs. Among the group identified as somewhat frail, 77% (492 out of 638) had no DRPs [130]. In the group with frailty, 62% had no DRPs [130]. For the group identified as very frail, 60% had no DRPs, and 40% had DRPs. These differences were statistically significant (*p* < 0.001) [130].

The studies by Ye et al. ([26,161]) investigated the risk of DRPs in different populations categorized by their frailty status, using the MRQ-10 (refer to Table 2). The MRQ-10, a validated tool, assesses risks associated with polypharmacy, inappropriate prescribing, poor adherence, and multiple medical problems. In the 2022 study [26], the group identified as non-frail had a significantly lower risk of DRPs, with 65.2% categorized as low risk and 34.8% as high risk at 12 months follow-up. Conversely, the group with pre-frailty exhibited a higher risk, with 52.4% at low risk and 47.6% at high risk, showing a significant difference with *p*-values of <0.001 for both categories [26].

The study by Alves et al. [71] assessed occurrences of drug–drug interactions (DDIs) at baseline using the Micromedex Drug Reax System across various frailty levels. In the group identified as non-frail, out of 181 individuals, 65 (35.9%) experienced DDIs [71]. In the group with pre-frailty, 131 out of 323 individuals (40.6%) had DDIs [71]. The group with frailty had the highest proportion, with 46 out of 76 individuals (60.5%) experiencing DDIs [71].

Athuraliya et al. [184] investigated the prevalence of DDIs based on the SFINX Monitor. They found that individuals with frailty were significantly more likely to experience negative DDIs compared to individuals identified as non-frail [184]. In the group identified as non-frail, 5.47% (195 out of 3550) were at risk of potential adverse DDIs, and 6.82% (242 out of 3550) experienced ADEs [184]. In the group with frailty, 18.22% (127 out of 684) were at risk of potential adverse DDIs, and 18.71% (128 out of 684) experienced ADEs [184] (see Table 2).

### 3.7. Impact of Medication Review on Polypharmacy Across Different Frailty Levels

The study by Molist-Brunet et al. [96] investigates the effects of medication review on polypharmacy (defined as the continuous use of ≥5 medications) across varying degrees of frailty, examining whether polypharmacy decreased, remained unaltered, or increased after the review. Among individuals with no frailty, 11.5% (4/32) experienced a decrease in polypharmacy, 69.3% (22/32) remained unchanged, and 19.2% (6/32) saw an increase, with no significant change (*p* > 0.05) [96]. In those with mild frailty, 22.1% (25/113) saw a decrease, 71.2% (80/113) were unaltered, and 6.7% (8/113) experienced an increase, with a significant *p*-value of 0.04 [96]. For moderate frailty, 21.6% (43/201) had a decrease, 76.6% (154/201) remained unchanged, and 1.8% (4/201) saw an increase, showing high significance (*p* < 0.001) [96]. In severe frailty, 54.5% (45/82) experienced a decrease in polypharmacy, while 45.5% (37/82) remained unchanged, and no increase was observed, with a highly significant *p*-value (*p* < 0.001) [96].

### 3.8. Association Between Polypharmacy, Hyper-Polypharmacy, Medication Use, PIMs, and Frailty

Table 3 provides a comprehensive analysis of the associations between medication use, polypharmacy, PIMs, DRPs, and frailty. The data are divided into various factors and their significant or non-significant associations based on different studies.

#### 3.8.1. Association Between Polypharmacy and Frailty

A total of 27 studies examined the association between polypharmacy and frailty. Of the 27 studies, 20 studies report a significant association between polypharmacy and frailty (refer to Table 3) ([25,26,37,46,47,77,80,83,101,105,110,117,129,133,186,189,191,209,217,251]). The strength of the association varied depending on how polypharmacy was defined. Studies by Aprahamian et al. and Aznar-Tortonda et al. found significant associations between using three or more drugs and the risk of frailty ([25,133]). Polypharmacy with ≥4 drugs was also found to be significantly associated with frailty in one study [25] but not in another [234].

However, the association with frailty became more consistent and robust when polypharmacy was defined as (≥5 drugs). Numerous studies, such as those by Aprahamian et al. [25], Ye et al. [26], Kurnat-Thoma et al. [37], Moon et al. [46], Closs et al. [77], Jung et al. [80], Gnjidic et al. [83], Cheung et al. [101], Badrkhahan et al. [110], de Godoi Rezende Costa Molino et al. [117], Thapaliya et al. [186], Setiati [191], Jazbar et al. [60], and Prieto-Contreras et al. [251], confirmed significant associations with an increase in the risk of frailty. However, studies by Huang et al. [73], Hasan et al. [128], and Arslan et al. [180] did not find significant associations.

Some studies explored even higher thresholds, such as ≥6 or ≥8 medications; for instance, polypharmacy (≥6 drugs) showed a positive association with frailty ([25,129]). In the case of polypharmacy with ≥8 drugs, one study found a positive association [189] while the second did not [221]. Polypharmacy (5–9 drugs) shows a positive association with frailty, with significant associations found by Saum et al. [47], Reallon et al. [105], and Herr et al. [217], while Chaouacha et al. [182] did not find a significant association. Polypharmacy (≥5 drugs) plus PIMs shows a positive association, as indicated by Aprahamian et al. [25].

#### 3.8.2. Association Between Hyper-Polypharmacy and Frailty

Six studies assessed the association between hyper-polypharmacy and frailty, all of which demonstrated a significant association between hyper-polypharmacy and frailty (refer to Table 3). Hyper-polypharmacy (both >9 or ≥10 drugs) shows a significant association with higher risk of frailty ([47,83,105,182,217]), as did both polypharmacy (≥5 drugs)[70].

#### 3.8.3. Association Between Medication Use and Frailty

A total of 13 studies reported a significant association between different medication use factors and frailty (refer to Table 3) ([60,83,105,128,142,165,171,178,195,205,223,227,234]). A higher average number of medications shows a positive association with frailty ([163,234]). Medications for high blood pressure, coronary and other heart diseases, diabetes, joint and other pain, anxiety, depression, sleep problems, osteoporosis, chronic bronchitis, and inflammation also showed a positive association [60]. Medication for high blood cholesterol showed a negative association with frailty [60]. Prescription of oral anticoagulation among those with non-valvular atrial fibrillation showed a positive association with frailty [195]. Metformin use shows a positive association with frailty [178]. However, another study determined no significant associations between oral anti-diabetic drugs, insulin, antihypertensives, and lipid-lowering drugs [226].

Antidepressant users with depressive symptoms show a positive association with frailty, as indicated by Lakey et al. [223]. First-generation and second-generation anti-psychotics show a positive association with frailty [165]. Exposure to anticholinergic medications, sedative medications, benzodiazepine, and sleep medication use shows a positive association with frailty ([105,142,171,234]).

#### 3.8.4. Association Between PIMs and Frailty

Seven studies have reported a significant association between PIMs (using any criteria) and frailty (refer to Table 3) ([26,45,51,70,71,160,168]). PIM use determined by 2012 and 2019 Beers Criteria show a positive association with frailty ([45,70]) while PIMs determined by 2015 Beers Criteria show mixed results. For example, Muhlack et al. [51] found positive associations, while Aprahamian et al. [25] and Hasan et al. [128] found no significant association. Beers Criteria to avoid cognitively impaired patients (BEERS dementia PIM), PRISCUS PIM, and EU (7) all show a positive association with frailty, as indicated by Muhlack et al. [51]. The Brazilian Consensus on Potentially Inappropriate Drugs for Older People shows a positive association with frailty, as indicated by Alves et al. [71]. START/STOPP version 2 criteria show no association: Tampaki et al. [91] and Hasan et al. [128] found no significant associations.

The Drug Burden Index (DBI) shows a positive association with frailty, with significant associations ([83,128,227]). Anticholinergic burden (ACB) shows a positive association with frailty, as indicated by Cheong et al. [160]. Anticholinergic drug scale (ADS) score (>0) shows a positive association with frailty, as indicated by Lampela et al. [168].

### 3.9. Recommendations for Addressing Drug-Related Problems (DRPs) in Older Adults with Frailty

This section highlights recommendations for addressing DRPs in older adults with frailty, providing a comprehensive overview of strategies and interventions designed to manage DRPs in this vulnerable population.

#### 3.9.1. Comprehensive Medication Review and Deprescribing

The cornerstone of addressing DRPs in older adults involves thorough medication reviews and targeted deprescribing practices. One notable intervention is the Family Conferences to Facilitate Deprescribing in Older Outpatients with Frailty and With Polypharmacy (COFRAIL) initiative [35]. The COFRAIL cluster randomized trial aimed to assess the impact of general practitioner (GP)-led family conferences on medication use and clinical outcomes in older adults with frailty and polypharmacy [35]. The study included community-dwelling adults aged 70 and older, taking at least five medications daily. The intervention group, which participated in structured family conferences, showed a temporary reduction in the number of medications at six months. At six months, the mean number of medications in the intervention group decreased from 8.98 (3.56) to 8.11 (3.2) compared to 9.24 (3.44) to 9.32 (3.59) in the control group with a statistically significant difference at six months in the mixed-effect Poisson regression model (*p* = 0.001). However, in twelve months, the difference was no longer significant, indicating a lack of sustained deprescribing effects over time. After six months, the mean (SD) number of EU (7)-PIMs was significantly lower in the intervention group (1.30 [1.05]) than in the control group (1.71 [1.25]; *p* = 0.04). There was no significant difference in the mean number of EU (7)-PIMs after 12 months. Commonly deprescribed medications included proton pump inhibitors, urate-lowering drugs, statins, and oral antidiabetics. Despite a modest reduction in medication use, the study did not find a significant reduction in hospitalizations between the intervention and control groups after twelve months.

#### 3.9.2. Pharmacist-Led Deprescribing Reviews

Nishtala et al. (2023) reported a post hoc analysis of an RCT to evaluate the impact of a pharmacist-led deprescribing intervention on anticholinergic burden in frail, community-dwelling older adults [92]. Using the Anticholinergic Cognitive Burden (ACB) scale, the intervention aimed to reduce high risk medication use, such as amitriptyline, diphenhydramine, and oxybutynin, to mitigate cognitive decline. While the pharmacist-led reviews led to a modest reduction (−0.23 mean reduction) in anticholinergic burden, the effect was small and nonsignificant overall (Hedges’ g = −0.04, 95% CI −0.26 to 0.19) [92]. Before the COVID-19 lockdown, a greater deprescribing effect was observed (mean ACB change of −0.38, 95% CI −0.84 to 0.04), but this was not sustained post-lockdown. Limited GP engagement and healthcare disruptions were key barriers to deprescribing success. These findings emphasize the need for stronger interdisciplinary collaboration and structured deprescribing strategies to reduce inappropriate medication use in frail older adults.

#### 3.9.3. Interdisciplinary Team Approaches

Molist-Brunet et al. (2022) conducted a quasi-experimental (uncontrolled pre–post) study to assess the impact of a patient-centered medication review on medication use in older adults with multimorbidity [96]. The intervention, conducted by an interdisciplinary team including a geriatrician and a clinical pharmacist, aimed to reduce inappropriate prescriptions and optimize pharmacological treatment based on individual health goals [96]. The mean number of chronic medications per patient decreased by 17.96%, from 8.13 (SD 3.87) to 6.67 (SD 3.72), after the intervention (*p* < 0.001). The Medication Regimen Complexity Index (MRCI) was reduced by 19.03% from 31.0 (SD 16.2) to 25.1 (SD 15.1), reflecting a simpler and more manageable medication regimen for patients. The Drug Burden Index (DBI), indicating sedative and anticholinergic medication exposure, decreased by 8.40% from 1.19 (SD 0.82) to 1.09 (SD 0.82) (*p* < 0.001). The greatest reduction in medication use and regimen complexity was observed in patients with severe frailty (*p*< 0.001). Frail patients were more likely to benefit from deprescribing interventions, particularly in reducing high risk medications.

#### 3.9.4. Deprescribing Tools and Criteria

The Marta Mejías-Trueba et al. (2023) study evaluated the prevalence of PIMs in polymedicated older adults with multimorbidity using two deprescribing tools: LESS-CHRON and STOPPFrail [99]. The median number of prescribed drugs per patient was 14.4, highlighting a high burden of polypharmacy in this population. LESS-CHRON identified 158 PIMs, while STOPPFrail detected 127 PIMs, with both tools showing a correlation with the number of prescribed drugs. LESS-CHRON was found to be more sensitive in detecting PIMs, particularly in patients with multimorbidity, whereas STOPPFrail provided complementary insights for frail patients with limited life expectancy. The most frequently flagged medications for deprescribing included benzodiazepines for insomnia, antidepressants for reactive depression, and lipid-lowering therapies. Despite their effectiveness, the study underscores the need for individualized deprescribing strategies to reduce inappropriate medication use and improve medication safety in older adults with complex health conditions.

#### 3.9.5. Multifactorial Interventions

A randomized clinical trial was conducted in a Barcelona primary healthcare center by Huguet et al. to evaluate the impact of a multifactorial intervention on preventing the progression from pre-frailty to frailty in community-dwelling adults aged 80 years and older [127]. The intervention included physical exercise, nutritional counseling (Mediterranean diet), social support, and medication review for polypharmacy using STOPP-START criteria.

Among 62 participants with inappropriate prescriptions, 48.4% (30/62) were resolved. After 12 months, frailty progression was lower in the intervention group (8.2%, n = 7) compared to the control group (23.9%, n = 21) (RR was 2.90 times higher in the CG (95% CI 1.45–8.69), with 14.1% (n = 12) of intervention participants reverting to robustness (*p* < 0.001). Frailty progression was linked to declining autonomy, functional status, and increased social risk, reinforcing the importance of structured interventions in preventing frailty and optimizing medication use in older adults.

Overall, these detailed recommendations and interventions focus on reducing adverse drug events, improving medication appropriateness, and enhancing the overall health and quality of life for frail older adults through a structured and patient-centered approach to medication management.

## 4. Discussion

This scoping review provides a comprehensive overview of the prevalence and management of frailty among older adults across different regions and populations. It highlights the significant variability in frailty prevalence and the impact of various assessment tools and methodologies. The discussion below synthesizes the key findings and implications, emphasizing the importance of tailored interventions and systematic approaches to managing frailty and associated DRPs in older adults.

### 4.1. Variability in Frailty Prevalence

This scoping review underscores the substantial variability in frailty prevalence across different countries and studies. For instance, in Taiwan, frailty prevalence ranges from 2.9% to 45.8% ([73,189]), while in Brazil, it spans from 9.4% to 67.4% ([64,109]). The Netherlands exhibits the most variability, with rates ranging from 11.6 to 55% ([200,250]). This wide range of prevalence highlights the influence of demographic factors, study designs, and frailty measurement tools. A frailty prevalence of 1.8% among individuals in BC, as determined through electronic medical records, suggests a relatively low burden of frailty [216]. The study notes that identifying frailty using electronic medical record data is particularly challenging because many functional deficits are not routinely recorded in structured data fields [216]. This limitation results in an under-capture of frailty cases. Differences in how frailty is identified in electronic medical records versus administrative data also contribute to the low prevalence. For instance, studies using administrative data might capture those residing in long-term care facilities or those who are terminally ill, both of whom might not be represented in primary care electronic medical records and represent populations where there is a higher prevalence of frailty. The study methodology and population might also play a role. For example, linked data from BC and Manitoba might not fully represent all individuals with frailty due to differences in healthcare utilization, reporting practices, and regional health demographics [216].

The prevalence of individuals with pre-frailty ranged from 11.8 to 72.9% ([125,225]). The study by Varan et al. had lower prevalence of pre-frailty due to the selection criteria implemented [125]. The study focused on community-dwelling older adults, who are generally more active and independent compared to those in assisted living or nursing homes. The EFS has specific scoring cut-offs that may categorize individuals differently compared to the FFI. Individuals who show mild declines in physical abilities (captured by FFI) may not meet the threshold for pre-frailty under the EFS unless additional domains are affected [125]. Similarly, as the mean age of the population studied increases, so does the prevalence of pre-frailty and frailty, as demonstrated by Pérez-Ros et al. in their study of individuals with a mean age 76.05 years [225], as well as a study conducted in Germany amongst a population with a mean age of 68.7 years and prevalence of frailty of 0.9% [76].

The choice of frailty assessment tools significantly impacts the reported prevalence rates. The article compares various tools, such as the 5-item FRAIL Scale and Fried’s Frailty Phenotype, revealing differing outcomes [7,255]. For instance, the 5-item FRAIL Scale, used by Hung et al. [189] in Taiwan, reported 22.43% of participants as frail. In contrast, Fried’s Frailty Phenotype, also utilized in the study by Hung et al. [189], showed a higher frailty rate of 45.85%. Similarly, the Edmonton Frail Scale (EFS) and the Clinical Frailty Scale (CFS) are two widely used frailty assessment tools that measure different components of frailty. For instance, the EFS might identify a higher prevalence of frailty due to its inclusion of cognitive and functional domains, whereas the CFS, which is more focused on physical performance and clinical judgment, might report a lower prevalence. Different frailty measures can significantly impact the reported outcomes in studies involving older adults [256]. The choice of frailty measure can also influence the observed relationship between frailty and outcomes such as medication use, hospitalization, and mortality [257]. This variability highlights the critical need for standardization in frailty definition and operationalization of frailty identification and monitoring.

### 4.2. Medication Use and Frailty

The scoping review delves into the relationship between medication use, polypharmacy, and frailty. It consistently finds that individuals with frailty tend to use more medications compared to their counterparts identified as non-frail. For instance, Aprahamian et al. [25] reported mean medication numbers of 2.56, 3.88, and 5.01 for individuals identified as non-frail, pre-frail, and frail, respectively, with significant differences (*p* < 0.001). Similarly, Gutiérrez-Zúñiga et al. [30] found mean medication numbers of 1.3, 3.2, and 6 for individuals identified as non-frail, pre-frail, and frail, respectively, also with significant differences (*p* < 0.001). Other studies, such as those by Moon et al. [46] and Gnjidic et al. [83], showed comparable trends. These results underscore a relationship between frailty and the number of medications. This relationship may be bidirectional—an increasing number of chronic conditions increases the risk of frailty but also may require more medications to be managed; however, increasing the use of medications may increase the risk of frailty as well.

Polypharmacy, defined as the use of multiple medications, is prevalent among individuals with frailty and pre-frailty [19]. The article highlights that polypharmacy rates significantly increase with frailty, with individuals with frailty consistently exhibiting higher rates of polypharmacy compared to individuals identified as non-frail and pre-frail. This association is robust across various study designs and populations, although some exceptions exist. Studies by Saeidimehr et al. [228] present atypical results where individuals identified as non-frail had higher polypharmacy prevalence. These exceptions underscore the need for context-specific analyses and interventions.

Definitions of polypharmacy varied slightly across studies, with most defining it as the use of ≥5 medications. Some studies had more specific definitions, such as the use of 5–9 medications per day or the concurrent use of ≥5 medications. The studies by Larsen et al. [57] and Kume et al. [68] explore the prevalence of polypharmacy among individuals identified as non-frail, pre-frail, and frail using different definitions. Larsen et al. [57] used two definitions: regular or occasional use of ≥9 medications and the use of ≥5 medications. The findings showed that with the first definition, 33.4%, 45%, and 52.7% of individuals identified as non-frail, pre-frail, and frail, respectively, experienced polypharmacy. With the second definition, the prevalence was higher, with 72.1%, 81.6%, and 85.5% of individuals identified as non-frail, pre-frail and frail, respectively. Similarly, Kume et al. [68] also used two definitions: the international classification (≥5 medications) and the Japanese classification (≥6 medications). According to the international classification, 18.8%, 29.4%, and 58.3% of individuals identified as non-frail, pre-frail, and frail, respectively, were on polypharmacy (*p* = 0.004) [68]. The Japanese classification showed 10.1% of non-frail, 19.6% of pre-frail, and 50% of frail individuals on polypharmacy (*p* = 0.001) [68].

### 4.3. Potentially Inappropriate Medications (PIMs)

Older adults with multiple health conditions often require numerous medications, increasing the risk of PIMs. The use of PIMs is notably higher among individuals with frailty, as indicated by various studies using criteria like the Beers Criteria and STOPP/START. For instance, Aprahamian et al. [25] using the Beers Criteria (2015) found PIM prevalence at 18.1%, 30.8%, and 46.5% in individuals identified as non-frail, pre-frail, and frail, respectively. Similarly, Bolina et al. [45] using the Beers Criteria (2012) reported 17%, 33.2%, and 51.1% in older adults identified as non-frail, pre-frail, and frail, respectively. Cox et al. [33] using the STOPP Fall tool indicated varied prevalence based on the number of Fall Risk Increasing Drugs (FRIDs), with the highest rates in the groups with frailty. Other studies, like Thiruchelvam et al. [49] and Uragami et al. [58], showed a clear trend of increasing PIM use from non-frail to frail categories using different versions of the Beers Criteria and STOPP-J. Durmuş et al. [179] found similar results using the TIME criteria, reporting PIMs in 37.5%, 44.5%, and 48.4% in individuals identified as non-frail, pre-frail, and frail, respectively.

The lack of a universally accepted definition of frailty complicates an understanding of the relationship between polypharmacy and PIMs and frailty. Moreover, this variability extends to the criteria used for determining PIMs as well, with tools such as the Beers Criteria, STOPP/START Criteria, and others showing different prevalences of PIMs among individuals with frailty. The discrepancies in frailty definitions and PIM criteria hinder the development of standardized interventions, emphasizing the need for a consensus on these definitions to enhance the quality of care for older adults with frailty [258]. Specific drug categories like antipsychotics, anticholinergics, and antidepressants are also associated with an increasing risk of frailty, as highlighted by Porter et al. [211].

### 4.4. Association Between Medication Use, Polypharmacy, and DRPs with Frailty and Its Implications

As mentioned previously, there is likely a bidirectional relationship between polypharmacy and PIM use and frailty [259]. Frailty significantly impacts medication use, as individuals with frailty are more likely to experience DRPs. Ye et al. [26] found that individuals with frailty had higher risks of DRPs at 12 months (OR: 1.75, *p* < 0.001), while Saum et al. [47] noted that hyper-polypharmacy (≥10 drugs) was associated with increased odds of frailty both at baseline and within a 3-year follow-up. Negative outcomes associated with polypharmacy and inappropriate medication use include falls, hospitalization, and mortality, particularly among individuals with frailty and pre-frailty [260].

### 4.5. Recommendations for Addressing DRPs

The report identifies several recommendations for managing DRPs in older adults with frailty. Comprehensive medication reviews and deprescribing practices, such as the COFRAIL initiative and pharmacist-led reviews, may be helpful [35]. These interventions involve interdisciplinary teams and structured approaches to assess and optimize medication use. Tools like the LESS-CHRON and SFINX Monitor can aid in identifying and addressing PIMs and drug–drug interactions ([99,184]).

Multifactorial interventions, combining several approaches, show promise in reducing sedative and anticholinergic medications and improving patient outcomes [172]. Randomized controlled trials (RCTs) evaluating these interventions highlight their effectiveness in lowering DBI scores and enhancing the quality of life for individuals with frailty.

The scoping review on medication use by older adults with frailty has several strengths and limitations. Among its strengths, the review employed a comprehensive methodology, utilizing a five-stage framework proposed by Arksey and O’Malley, supplemented by the PRISMA Extension for Scoping Reviews (PRISMA-ScR). This structured approach ensures thoroughness in identifying, selecting, and analyzing relevant studies. The literature search was extensive, covering multiple databases, which increased the likelihood of capturing all relevant studies. Additionally, the review clearly defined eligibility criteria, focusing on older adults with frailty living at home, and excluding non-English publications and studies without medication-related health outcomes, thereby maintaining the focus and relevance of the included studies. Data regarding study design, demographics, frailty measures, and outcomes were systematically extracted and analyzed, with multiple reviewers enhancing the reliability of the data. The review identified significant findings related to the prevalence of polypharmacy, hyper-polypharmacy, and PIMs among individuals with frailty, as well as the association between polypharmacy and increased odds of frailty.

However, the review also has limitations. There is a lack of a universally accepted definition of frailty and the use of various frailty measurement tools, leading to inconsistencies in diagnosis and treatment, which complicate comparisons across studies and may impact the generalizability of the findings. The exclusion of non-English publications introduces potential language bias, possibly overlooking relevant studies from non-English speaking regions. The heterogeneity of the included studies, in terms of design, population characteristics, and methodologies, poses challenges in synthesizing the findings and drawing definitive conclusions. Despite the comprehensive search strategy, there is always a possibility of selection bias, where some relevant studies might have been missed or excluded during the screening process. The review’s focus on community-dwelling older adults may limit the applicability of the findings to those in hospital or institutional settings where medication use patterns and frailty prevalence might differ. Our search ended in November 2023 and does not include more recent studies, and country-level data for 2024–2025 remain incomplete, leaving a knowledge gap for these years. Another limitation is that our review did not quantitatively analyze the range of participant ages or the length of follow-up used to assess frailty across the included studies. These factors likely contribute to the observed heterogeneity in frailty prevalence and outcomes across countries and study settings.

An additional consideration that may affect the generalizability of these findings is the role of multiple prescribers and transitions of care. Older adults, particularly those with frailty or multimorbidity, often receive prescriptions from several healthcare providers across different settings (e.g., primary care, specialists, hospital discharge). This fragmentation increases the risk of duplicate therapy, drug–drug interactions, and discrepancies in medication reconciliation. Furthermore, transitions between care settings—such as hospital to community or long-term care—represent vulnerable periods when medication regimens may change abruptly without adequate communication among prescribers. These factors can significantly contribute to the development or worsening of potentially inappropriate medication use and related geriatric syndromes.

## 5. Conclusions

In conclusion, the article highlights the significant variability in frailty prevalence and the impact of assessment tools and methodologies on reported rates. It emphasizes the complex relationship between medication use, polypharmacy, and frailty, underscoring the need for tailored and systematic approaches to manage DRPs. Comprehensive medication reviews, interdisciplinary approaches, and targeted interventions are crucial in optimizing medication use and improving the health outcomes of frail older adults. Standardizing frailty assessment tools and methodologies can enhance comparability across studies and inform more effective and targeted public health interventions.

## Figures and Tables

**Table 1:** Prevalence of potentially inappropriate medications (PIMs) among older adults based on frailty.

Study ID Country	PIMs Criteria Used	Category	Non-Frail (%)	Pre-Frail (%)	Frail (%)
Bolina., 2019 [45] (Brazil)	Beers 2012	At least one PIM	94/554 = 17	277/834 = 33.2	112/219 = 51.1
Aprahamian., 2018 * [25] (Brazil)	Beers 2015	At least one PIM	36/199 = 18.1	102/331 = 30.8	46/99 = 46.5
Athuraliya., 2022 [184] (Australia)	Beers 2019	At least one PIM	221/3550 = 6.2	N/A	97/684 = 13.9
Thiruchelvam., 2021 [40] (Australia)	Beers 2019	At least one PIM in non-continuous polypharmacy	4046/5655 = 71.5	N/A	993/1263 = 78.6
		At least one PIM in continuous polypharmacy	1174/1258 = 93.3	N/A	786/820 = 95.9
Thiruchelvam., 2021 [49] (Australia)	Beers 2019	At least one PIM	5316/7218 = 73.6	N/A	1811/2137 = 84.7
Lockery., 2020 [70] (Australia, USA)	Beers 2019	At least one PIM	55/116 = 47.4	42/79 = 53.2	3/5 = 60.0
Muhlack., 2018 [52] (Germany)	Beers 2019	At least one PIM	N/A	N/A	119/265 = 44.9
	EU (7)-PIM List				153/265 = 57.7
	PRISCUS List				71/265 = 26.8
Porter., 2019 [211] (UK)	STOPP/START	Antipsychotic	4/204 = 2	7/530 = 1.3	10/420 = 2.4
		Anticholinergics	6/204 = 2.9	36/530 = 6.8	36/420 = 8.6
		Tricyclic antidepressants	5/204 = 2.5	30/530 = 5.6	41/420 = 9.8
		Other antidepressants	14/204 = 6.9	35/530 = 6.6	70/420 = 16.7
		Benzodiazepines	4/204 = 2	14/530 = 2.6	28/420 = 6.7
		Proton pump inhibitors	34/204 = 16.7	149/530 = 28.1	148/420 = 35.2
Cox., 2023 * [33] (UK)	STOPP	No FRIDs	14/39 = 35.9%	12/46 = 26.1%	1/15 = 6.67%
		1 FRID	16/39 = 41.0%	9/46 = 19.6%	1/15 = 6.67%
		>1 FRID	9/39 = 23.1%	25/46 = 54.3%	13/15 = 86.7%
Uragami., 2021 * [58] (Japan)	STOPP-J	At least one PIM	208/556 = 37.4	178/318 = 56	34/49 = 69.4
Khera 2019 [90] (Canada)	STOPP/START v2 and Beers 2015	At least one PIM in Frailty	N/A	N/A	31/54 = 57.4
Durmuş., 2023 [179] (Turkey)	TIME	PIM	6/16 = 37.5	81/182 = 44.5	89/184 = 48.4
	Beers 2019	PIM	4/16 = 25.0	52/182 = 28.6	61/184 = 33.2
	STOPP/START v2	PIM	4/16 = 25.0	42/182 = 23.1	52/184 = 28.3
Alves., 2020 * [71] (Brazil)	Brazilian Consensus on PIM for Older People		53/181 = 29.3	160/323 = 49.5	41/76 = 53.9

* = Statistically significant; Beers: American Geriatrics Society Beers Criteria for Potentially Inappropriate Medication; EU: European Union 7 potentially inappropriate medication list; FRID: Falls risk increasing drug.

**Table 2:** Prevalence of drug-related problems (DRPs) among older adults based on frailty.

Study ID Country	Criteria Used to Identify DRP	Category	Non-Frail (%)	Pre-Frail (%)	Frail (%)
(Ye., 2022) * [26] (Netherlands, Greece, Croatia, Spain, United Kingdom)	MRQ-10	Low risk ^	525/804 = 65.2	N/A	513/979 = 52.4
		High risk ^	279/804 = 34.8	N/A	466/979 = 47.6
(Ye., 2021) * [161] (UK, Greece, Croatia, Netherlands, Spain)	MRQ-10	Mean score	4.06 (1.51)	N/A	4.67 (1.68)
(Sargent., 2020) * [55] (Italy)	Anticholinergic Burden	ACB 1	N/A	N/A	117/512 = 22.9
		ACB 2	N/A	N/A	61/512 = 11.9
		ACB 3	N/A	N/A	45/512 = 8.8
(Cheong., 2023) [160] (UK)	Anticholinergic Burden	ACB 1	72,010/255,402 = 28.2	N/A	Mild: 99,685/185,902 = 53.6 Moderate: 47,608/69,867 = 68.1 Severe: 13,914/17,924 = 77.6
		ACB 2	170/255,402 = <0.1	N/A	Mild: 269/185,902 = 0.2 Moderate: 168/69,867 = 0.2 Severe: 62/17,924 = 0.4
		ACB 3	12,508/255,402 = 4.9	N/A	Mild: 21,582/185,902 = 11.6; Moderate: 11,239/69,867 = 16.1 Severe: 3438/17,924 = 19.2
(Rhalimi 2018) * [130] (France)	SEGA	No DRP	N/A	N/A	Somewhat: 492/638 = 77 Frail: 75/121 = 62 Very: 79/131 = 60.3
		DRP	N/A	N/A	Somewhat: 146/638 = 23 Frail: 46/121 = 38 Very: 52/131 = 40
(Athuraliya., 2022) [184] (Australia)	SFINX	Potential adverse DDI	195/3550 = 5.47	N/A	127/684 = 18.22
		Frequency of any ADE	242/3550 = 6.82	N/A	128/684 = 18.71

* = Statistically significant; ^ = risk of medication-related problems at 12 months follow-up; medication-related follow-up using the Medication Risk Questionnaire. MRQ is a 10-item validated self-administered tool that can identify participants at a higher risk of medication-related problems, notably in the older population. It covers polypharmacy, inappropriate prescribing, poor adherence, and multiple medical problems; SEGA = Short Emergency Geriatric Assessment grid by French Society of Clinical Pharmacy; SFINX = Swedish Finnish Interaction X-reference Monitor (Quality Pharma AB, Sweden). The participants were defined as at risk of potential adverse drug–drug interactions (aDDI) if the Swedish Finnish Interaction X-reference (SFINX) Monitor (Quality Pharma AB, Sweden), identified a clinically significant aDDI of class C (interactions managed by dose reduction) or D (interactions always avoided) out of 4 classes (from A to D) with a high level of evidence (3 and 4 from levels 1 to 4). ADE = Mental and behavioral disorder due to multiple drug use, medicaments, and biological substances causing adverse effects, Unspecified adverse effects of drugs and medicaments, Accidental poisoning by and exposure to other drugs acting on ANS, CVS, and gastrointestinal-related ADE; ADE markers = Non-accidental falls events, Acute delirium, Total AKI events, Drug class associated with AKI: NSAID, Diuretics, Beta-blockers.

**Table 3:** Associations (from adjusted models) between medication use, polypharmacy, PIMs, DRPs, and frailty.

Factor	Studies with No Significant Association (*p* ≥ 0.05)	Studies with Significant Association (*p* < 0.05)	Type of Association
Polypharmacy			
≥3 drugs		(Aprahamian., 2018; Brazil) [25] (1), (Aznar-Tortonda., 2020; Spain) [133] (8)	Positive
≥4 drugs	(JeanWoo., 2015; China) [234] (2)	(Aprahamian., 2018; Brazil) [25] (1)	
≥5 drugs	(Huang., 2018; Taiwan) [73] (3), (Hasan., 2018; Malaysia) [128] (3), (Arslan., 2023; Turkey) [180] (2)	(Aprahamian., 2018; Brazil) [25] (1), (Ye., 2022; Netherlands, Greece, Croatia, Spain, United Kingdom) [26] (2), (Kurnat-Thoma., 2022; USA) [37] (3), (Moon., 2019; South Korea) [46] (3), (Closs., 2016; Brazil) [77] (4), (Jung., 2016; Korea) [80] (8), (Gnjidic., 2012; Australia) [83] (8), (Cheung 2020; Hong Kong, Israel, European countries) [101] (10), #1716 (Badrkhahan 2023; Iran) [110] (3), (DeGodoiRezendeCostaMolino 2022; European countries: Basel, Berlin, Coimbra, Geneva, Innsbruck, Toulouse, and Zurich) [117] (3), (Thapaliya., 2021; Australia) [186] (3), (Setiati., 2021; Indonesia) [191] (3), (Jazbar., 2021; Slovenia) [209] (9), (Prieto-Contreras., 2023; Spain) [251] (8)	Positive
≥6 drugs	(Kume., 2021; Japan) [36] (3)	(Aprahamian., 2018; Brazil) [25] (1), (Yuki 2018; Japan) [129] (8)	Positive
≥8 drugs	(Pao., 2018; Taiwan) [221] (8)	(Hung., 2021; Taiwan) [189] (8)	
5–9 drugs	(Chaouacha., 2022; Oman) [182] (4)	(Saum., 2017; Germany) [47] (5,6), (Reallon 2020; France) [105] (4), (Herr., 2015; France) [217] (3)	Positive
≥5 drugs plus PIM		(Aprahamian., 2018; Brazil) [25] (1)	Positive
Hyper-polypharmacy			
≥10 drugs		(Saum., 2017; Germany) [47] (5,6), (Gnjidic., 2012; Australia) [83] (8), (Herr., 2015; France) [217] (3)	Positive
(≥5 drugs) and (≥10 drugs)		(Lockery., 2020; Australia, USA) [70] (3)	Positive
(>9 drugs)		(Reallon 2020; France) [105] (4), (Chaouacha., 2022; Oman) [182] (4)	Positive
Medication use			
Number of medications		(Gnjidic 2012; Australia) [83] (8), (Woo., 2015; China) [234] (3)	Positive
Medication for high blood pressure, coronary and other heart diseases, diabetes, joint and other pain, anxiety, depression and sleep problems, osteoporosis, chronic bronchitis, inflammation		(Jazbar., 2021; Austria, Germany, Sweden, Spain, Italy, France, Denmark, Switzerland, Belgium, Israel, Czech Republic, Luxembourg, Slovenia, and Estonia) [60] (3)	Positive
Medication for high blood cholesterol		(Jazbar., 2021; Austria, Germany, Sweden, Spain, Italy, France, Denmark, Switzerland, Belgium, Israel, Czech Republic, Luxembourg, Slovenia, and Estonia) [60] (3)	Negative
Use of sleep medication		(Arias-FernÃ¡ndez., 2021; Spain) [142] (8), (Mizuno., 2023; Japan) [171] (8), (Woo., 2015; China) [234] (3)	Positive
Current statin users, < 1 years of statin medication use, 1–3 years of statin medication use, >3 years of statin medication use	(LaCroix., 2008; USA) [164] (4)		No association between dose and duration of statin use
First- and second-generation anti-psychotics		(Gafoor., 2019; UK) [165] (9)	Positive
Benzodiazepine use		(Mizuno., 2023; Japan) [171] (8)	Positive
Metformin use		(Liu., 2023; China) [178] (1)	Positive
Oral anti-diabetic drugs, insulin, antihypertensives, lipid-lowering drugs	(García-Esquinas., 2015; Spain) [226] (8)		No association
Antidepressant users with depressive symptoms		(Lakey., 2012; USA) [223] (4)	Positive
Prescription of oral anticoagulation, among those with non-valvular atrial fibrillation; use of DOAC vs. warfarin, among those receiving OAC		(Sanghai., 2022; USA) [195] (8)	Positive
Drug Burden Index		(Gnjidic., 2012; Australia) [83] (8), (Hasan 2018; Malaysia) [128] (3), (Byrne., 2019; Ireland) [227] (3)	Positive
Cumulative exposure to anticholinergic and sedative drugs (Drug Burden Index)		(Reallon 2020; France) [105] (11)	Positive
PIMs			
PIMs (2019 Beers Criteria)		(Lockery., 2020; Australia, USA) [70] (3)	Positive association
PIMs (2015 Beers Criteria)	(Aprahamian., 2018; Brazil) [25] (1), (Hasan 2018; Malaysia) [128] (3)	(Muhlack., 2019; Germany) [51] (7)	No association
PIMs (2012 Beers Criteria)		(Bolina., 2019; Brazil) [45] (4)	Positive
Beers Criteria to avoid cognitively impaired patients (BEERS dementia PIM)		(Muhlack., 2019; Germany) [51] (7)	Positive
PRISCUS PIM		(Muhlack., 2019; Germany) [51] (7)	Positive
EU (7) PIM		(Muhlack., 2019; Germany) [51] (7)	Positive
Brazilian Consensus on PIMs for Older People		(Alves., 2020; Brazil) [71] (3)	Positive
START/STOPP version 2 criteria	(Tampaki 2023; Greece) [91] (9), (Hasan 2018; Malaysia) [128] (3)		No association
Anticholinergic burden		(Cheong., 2023; UK) [160] (8)	Positive
Anticholinergic drug scale (ADS) score (>0)		(Lampela., 2016; Finland) [168] (8)	Positive
Drug-related problems			
Risk of MRPs		(Ye., 2022; Netherlands, Greece, Croatia, Spain, United Kingdom) [26] (2)	Positive

^1^ Binary logistic regression; ^2^ Hierarchical logistic regression analyses; ^3^ Multivariate logistic regression; ^4^ Multinomial logistic regression; ^5^ Cross-sectional logistic regression; ^6^ Longitudinal logistic regression; ^7^ Cox proportional hazard regression model; ^8^ Logistic regression; ^9^ Multiple logistic regression; ^10^ Ordinal logistic regression; ^11^ Stepwise multinomial logistic regression. Example of positive association: Significantly increased odds for pre-frailty and frailty were confirmed for the following medication groups: high blood pressure (for pre-frailty and for frailty) (Jazbar., 2021) [60] ^3^. Example of negative association: Taking medications for high blood cholesterol was significantly associated with lower odds for frailty (Jazbar., 2021) [60] ^3^. American Geriatrics Society Beers Criteria for Potentially Inappropriate Medication Use in Older Adults (Beers Criteria) and Screening Tool of Older People’s Prescriptions (STOPP) Criteria; Turkish Inappropriate Medication Use in the Elderly.

## Data Availability

No new data were created or analyzed in this study.

## Supplementary Materials

The following supporting information can be downloaded at https://www.mdpi.com/article/10.3390/pharmacy13060170/s1, Table S1: Preferred Reporting Items for Systematic reviews and Meta-Analyses extension for Scoping Reviews (PRISMA-ScR) Checklist; Table S2: Search Strategies; Table S3: Characteristics of the included studies (n = 223); Table S4: Studies reporting mean or median number of medications in older adults with different frailty status; Table S5: Distribution of patients across frailty groups based on medications prescribed; Table S6: Distribution of polypharmacy based on frailty status; Table S7: Distribution of polypharmacy and hyper-polypharmacy based on frailty status.

## References

[1] World Health Organization Ageing and Health. https://www.who.int/news-room/fact-sheets/detail/ageing-and-health.

[2] Government of Canada, Statistics Canada A Portrait of Canada’s Growing Population Aged 85 and Older from the 2021 Census. 27 April 2022. https://www12.statcan.gc.ca/census-recensement/2021/as-sa/98-200-X/2021004/98-200-X2021004-eng.cfm.

[3] Fillenbaum G.G., Pieper C.F., Cohen H.J., Cornoni-Huntley J.C., Guralnik J.M. (2000). Comorbidity of five chronic health conditions in elderly community residents: Determinants and impact on mortality. J. Gerontol. Ser. A Biol. Sci. Med. Sci..

[4] Roberts K.C., Rao D.P., Bennett T.L., Loukine L., Jayaraman G.C. (2015). Prevalence and patterns of chronic disease multimorbidity and associated determinants in Canada. Health promotion and chronic disease prevention in Canada: Research, policy and practice. Health Promot. Chronic Dis. Prev. Can..

[5] Tam T. (2020). Aging and Chronic Diseases: A Profile of Canadian Seniors.

[6] Levers M., Estabrooks C.A., Kerr J.C.R. (2006). Factors contributing to frailty: Literature review. J. Adv. Nurs..

[7] Fried L.P., Tangen C.M., Walston J., Newman A.B., Hirsch C., Gottdiener J., Seeman T., Tracy R., Kop W.J., Burke G. (2001). Frailty in older adults: Evidence for a phenotype. J. Gerontol. Ser. A.

[8] Feng Z., Lugtenberg M., Franse C., Fang X., Hu S., Jin C., Raat H. (2017). Risk factors and protective factors associated with incident or increase of frailty among community-dwelling older adults: A systematic review of longitudinal studies. PLoS ONE.

[9] Gobbens R.J.J., Luijkx K.G., Wijnen-Sponselee M.T., Schols J.M.G.A. (2010). Towards an integral conceptual model of frailty. J. Nutr. Health Aging.

[10] Kaufman S.R. (1994). The social construction of frailty: An anthropological perspective. J. Aging Stud..

[11] Sciacchitano S., Carola V., Nicolais G., Sciacchitano S., Napoli C., Mancini R., Rocco M., Coluzzi F. (2024). To be frail or not to be frail: This is the question—A critical narrative review of frailty. J. Clin. Med..

[12] Fulop T., Larbi A., Witkowski J.M., McElhaney J., Loeb M., Mitnitski A., Pawelec G. (2010). Aging, frailty and age-related diseases. Biogerontology.

[13] Schulz R., Sherwood P.R. (2008). Physical and mental health effects of family caregiving. J. Soc. Work. Educ..

[14] O’caoimh R., McGauran J., O’donovan M.R., Gillman C., O’hea A., Hayes M., O’connor K., Moloney E., Alcock M. (2022). Frailty screening in the emergency department: Comparing the variable indicative of placement risk, clinical frailty scale and PRISMA-7. Int. J. Environ. Res. Public Health.

[15] Lunenfeld B., Stratton P. (2013). The clinical consequences of an ageing world and preventive strategies. Best. Pract. Res. Clin. Obstet. Gynaecol..

[16] Canadian Frailty Network AVOID Frailty. https://www.cfn-nce.ca/frailty-matters/avoid-frailty/#:~:text=Over%201.6%20million%20older%20Canadians,older%20adults%20living%20with%20frailty.

[17] Matos A.D., Barbosa F., Cunha C., Voss G., Correia F. (2021). Social isolation, physical inactivity and inadequate diet among European middle-aged and older adults. BMC Public Health.

[18] Ofori-Asenso R., Chin K.L., Mazidi M., Zomer E., Ilomaki J., Zullo A.R., Gasevic D., Ademi Z., Korhonen M.J., LoGiudice D. (2019). Global incidence of frailty and prefrailty among community-dwelling older adults: A systematic review and meta-analysis. JAMA Netw. Open.

[19] Toh J.J.Y., Zhang H., Soh Y.Y., Zhang Z., Wu X.V. (2023). Prevalence and health outcomes of polypharmacy and hyperpolypharmacy in older adults with frailty: A systematic review and meta-analysis. Ageing Res. Rev..

[20] Hubbard R.E., O’mahony M.S., Woodhouse K.W. (2013). Medication prescribing in frail older people. Eur. J. Clin. Pharmacol..

[21] Akkawi M.E., Aziz H.H.A., Nahas A.R.F. (2023). The impact of potentially inappropriate medications and polypharmacy on 3-month hospital readmission among older patients: A retrospective cohort study from Malaysia. Geriatrics.

[22] Beckman A., Bernsten C., Parker M.G., Thorslund M., Fastbom J. (2005). The difficulty of opening medicine containers in old age: A population-based study. Pharm. World Sci..

[23] Arksey H., O’Malley L. (2005). Scoping studies: Towards a methodological framework. Int. J. Soc. Res. Methodol..

[24] Tricco A.C., Lillie E., Zarin W., O’Brien K.K., Colquhoun H., Levac D., Moher D., Peters M.D.J., Horsley T., Weeks L. (2018). PRISMA Extension for Scoping Reviews (PRISMA-ScR): Checklist and Explanation. Ann. Intern. Med..

[25] Aprahamian I., Biella M.M., De Almeida G.V.A., Pegoraro F., Pedrini A.V.A., Cestari B., Bignotto L.H., De Melo B.A.R., Martinelli J.E. (2018). Polypharmacy but not potential inappropriate prescription was associated with frailty in older adults from a middle-income country outpatient clinic. J. Frailty Aging.

[26] Ye L., Yang-Huang J., Franse C.B., Rukavina T., Vasiljev V., Mattace-Raso F., Verma A., Borrás T.A., Rentoumis T., Raat H. (2022). Factors associated with polypharmacy and the high risk of medication-related problems among older community-dwelling adults in European countries: A longitudinal study. BMC Geriatr..

[27] Derhem B., Özsari S. (2023). Frailty and Polypharmacy in Primary Care. Biol. Res. Nurs..

[28] Pala F., Yalçin Gürsoy M. (2023). Prevalence of Frailty and Related Factors Among Community-Dwelling Older Adults: A Cross-Sectional Study from Western Türkiye. Turk. Klin. J. Nurs. Sci..

[29] Tchalla A., Laubarie-Mouret C., Cardinaud N., Gayot C., Rebiere M., Dumoitier N., Rudelle K., Druet-Cabanac M., Laroche M.-L., Boyer S. (2022). Risk factors of frailty and functional disability in community-dwelling older adults: A cross-sectional analysis of the FREEDOM-LNA cohort study. BMC Geriatr..

[30] Gutiérrez-Zúñiga R., Davis J.R., Ruddy K., De Looze C., Carey D., Meaney J., Kenny R.A., Knight S.P., Romero-Ortuno R. (2023). Structural brain signatures of frailty, defined as accumulation of self-reported health deficits in older adults. Front. Aging Neurosci..

[31] Santos P.H.S., dos Santos L., Fernandes M.H., Brito T.A., Munaro H.L.R., Carneiro J.A.O. (2023). Factors associated with frailty syndrome in older adults with three-and four-criteria clustering. Geriatr. Nurs..

[32] Chaitoff A., Haff N., Lauffenburger J.C., Choudhry N.K. (2023). Medication de-escalation opportunities among frail older adults with strictly-controlled cardiometabolic disease. J. Am. Geriatr. Soc..

[33] Cox N., Ilyas I., Roberts H.C., Ibrahim K. (2023). Exploring the prevalence and types of fall-risk-increasing drugs among older people with upper limb fractures. Int. J. Pharm. Pract..

[34] Sobhani A., Sharifi F., Fadayevatan R., Kamrani A.A.A., Moodi M., Khorashadizadeh M., Kazemi T., Khodabakhshi H., Fakhrzadeh H., Arzaghi M. (2022). Low physical activity is the strongest factor associated with frailty phenotype and frailty index: Data from baseline phase of Birjand Longitudinal Aging Study (BLAS). BMC Geriatr..

[35] Mortsiefer A., Löscher S., Pashutina Y., Santos S., Altiner A., Drewelow E., Ritzke M., Wollny A., Thürmann P., Bencheva V. (2022). Family conferences to facilitate deprescribing in older outpatients with Frailty and with polypharmacy: The COFRAIL Cluster Randomized trial. JAMA Netw. Open.

[36] Kume Y., Kodama A., Takahashi T., Lee S., Makizako H., Ono T., Shimada H., Ota H. (2022). Social frailty is independently associated with geriatric depression among older adults living in northern Japan: A cross-sectional study of ORANGE registry. Geriatr. Gerontol. Int..

[37] Kurnat-Thoma E.L., Murray M.T., Juneau P. (2022). Frailty and determinants of health among older adults in the United States 2011–2016. J. Aging Health.

[38] de Sousa C.R., Coutinho J.F.V., Neto J.B.F., Barbosa R.G.B., Marques M.B., Diniz J.L. (2021). Factors associated with vulnerability and fragility in the elderly: A cross-sectional study. Rev. Bras. Enferm..

[39] Österdahl M.F., Sinnott S.-J., Douglas I., Clegg A., Tomlinson L., Wong A. (2022). Frailty and rate of fractures in patients initiating antihypertensive medications: A cohort study in primary care. Drugs Ther. Perspect..

[40] Thiruchelvam K., Byles J., Hasan S.S., Egan N., Kairuz T. (2021). Prevalence and association of continuous polypharmacy and frailty among older women: A longitudinal analysis over 15 years. Maturitas.

[41] Rhalimi M., Housieaux E., Mary A., Detuncq C., Muller A., Georgin F., Comby F., Wehrlé C., Davoust N., Brazier M. (2021). Role of the community pharmacist in detecting frailty and spatio-temporal disorientation among community-dwelling older people in France. Aging Clin. Exp. Res..

[42] Chen M.Z., Wong M.W.K., Lim J.Y., Merchant R.A. (2021). Frailty and quality of life in older adults with metabolic syndrome—Findings from the healthy older people everyday (HOPE) study. J. Nutr. Health Aging.

[43] McKechnie D.G.J., Papacosta A.O., Lennon L.T., Ramsay S.E., Whincup P.H., Wannamethee S.G. (2021). Associations between inflammation, cardiovascular biomarkers and incident frailty: The British Regional Heart Study. Age Ageing.

[44] Bonfiglio V., Umegaki H., Kuzuya M. (2019). Potentially inappropriate medications and polypharmacy: A study of older people with mild cognitive impairment and mild dementia. J. Alzheimer’s Dis..

[45] Bolina A.F., Gomes N.C., Marchiori G.F., Pegorari M.S., Tavares D.M.d.S. (2019). Potentially inappropriate medication use and frailty phenotype among community-dwelling older adults: A population-based study. J. Clin. Nurs..

[46] Moon J.H., Huh J.S., Won C.W., Kim H.J. (2019). Is polypharmacy associated with cognitive frailty in the elderly? Results from the Korean frailty and aging cohort study. J. Nutr. Health Aging.

[47] Saum K., Schöttker B., Meid A.D., Holleczek B., Haefeli W.E., Hauer K., Brenner H. (2017). Is polypharmacy associated with frailty in older people? Results from the ESTHER cohort study. J. Am. Geriatr. Soc..

[48] Ye L., Nieboer D., Yang-Huang J., Borrás T.A., Garcés-Ferrer J., Verma A., van Grieken A., Raat H. (2023). The association between frailty and the risk of medication-related problems among community-dwelling older adults in Europe. J. Am. Geriatr. Soc..

[49] Thiruchelvam K., Byles J., Hasan S.S., Egan N., Kairuz T. (2021). Frailty and potentially inappropriate medications using the 2019 Beers Criteria: Findings from the Australian Longitudinal Study on Women’s Health (ALSWH). Aging Clin. Exp. Res..

[50] Callahan K.E., Lenoir K.M., Usoh C.O., Williamson J.D., Brown L.Y., Moses A.W., Hinely M., Neuwirth Z., Pajewski N.M. (2022). Using an Electronic Health Record and Deficit Accumulation to Pragmatically Identify Candidates for Optimal Prescribing in Patients With Type 2 Diabetes. Diabetes Spectr..

[51] Muhlack D.C., Hoppe L.K., Saum K.-U., Haefeli W.E., Brenner H., Schöttker B. (2020). Investigation of a possible association of potentially inappropriate medication for older adults and frailty in a prospective cohort study from Germany. Age Ageing.

[52] Muhlack D.C., Hoppe L.K., Stock C., Haefeli W.E., Brenner H., Schöttker B. (2018). The associations of geriatric syndromes and other patient characteristics with the current and future use of potentially inappropriate medications in a large cohort study. Eur. J. Clin. Pharmacol..

[53] Bergen A.W., Cil G., Sargent L.J., Dave C.V. (2022). Frailty risks of prescription analgesics and sedatives across frailty models: The health and retirement study. Drugs Aging.

[54] Ekram A.R.M.S., Woods R.L., Ryan J., Espinoza S.E., Gilmartin-Thomas J.F., Shah R.C., Mehta R., Kochar B., Lowthian J.A., Lockery J. (2022). The association between polypharmacy, frailty and disability-free survival in community-dwelling healthy older individuals. Arch. Gerontol. Geriatr..

[55] Sargent L., Nalls M., Amella E.J., Mueller M., Lageman S.K., Bandinelli S., Colpo M., Slattum P.W., Singleton A., Ferrucci L. (2020). Anticholinergic drug induced cognitive and physical impairment: Results from the InCHIANTI study. J. Gerontol. Ser. A.

[56] Naharci M.I., Tasci I. (2020). Frailty status and increased risk for falls: The role of anticholinergic burden. Arch. Gerontol. Geriatr..

[57] Larsen R.T., Turcotte L.A., Westendorp R., Langberg H., Hirdes J.P. (2020). Frailty index status of Canadian home care clients improves with exercise therapy and declines in the presence of polypharmacy. J. Am. Med. Dir. Assoc..

[58] Uragami Y., Takikawa K., Kareki H., Kimura K., Yamamoto K., Iihara N. (2021). Effect of number of medications and use of potentially inappropriate medications on frailty among early-stage older outpatients. J. Pharm. Health Care Sci..

[59] Midão L., Brochado P., Almada M., Duarte M., Paúl C., Costa E. (2021). Frailty status and polypharmacy predict all-cause mortality in community dwelling older adults in Europe. Int. J. Environ. Res. Public Health.

[60] Jazbar J., Locatelli I., Kos M. (2021). The association between medication or alcohol use and the incidence of frailty: A retrospective cohort study. BMC Geriatr..

[61] Kim M.J., Jang S.Y., Cheong H.-K., Oh I.-H. (2021). Association of frailty with healthcare costs using claims data in Korean older adults aged 66. J. Nutr. Health Aging.

[62] Gulliford M., Ravindrarajah R., Hamada S., Jackson S., Charlton J. (2017). Inception and deprescribing of statins in people aged over 80 years: Cohort study. Age Ageing.

[63] Jamsen K.M., Bell J.S., Hilmer S.N., Kirkpatrick C.M.J., Ilomäki J., Le Couteur D., Blyth F.M., Handelsman D.J., Waite L., Naganathan V. (2016). Effects of changes in number of medications and drug burden index exposure on transitions between frailty states and death: The concord health and ageing in men project cohort study. J. Am. Geriatr. Soc..

[64] Fernandes T.G., Silva K.R., Guerra R.O., Parente R.C.P., Borges G.F., Junior R.C.F. (2021). Influence of the Amazonian context on the frailty of older adults: A population-based study. Arch. Gerontol. Geriatr..

[65] Coelho T., Paúl C., Gobbens R.J.J., Fernandes L. (2015). Determinants of frailty: The added value of assessing medication. Front. Aging Neurosci..

[66] Martinot P., Landré B., Zins M., Goldberg M., Ankri J., Herr M. (2018). Association between potentially inappropriate medications and frailty in the early old age: A longitudinal study in the GAZEL cohort. J. Am. Med. Dir. Assoc..

[67] Lee H., Chong J., Jung H.-W., Baek J.Y., Lee E., Jang I.-Y. (2021). Association of the FRAIL scale with geriatric syndromes and health-related outcomes in Korean older adults. Ann. Geriatr. Med. Res..

[68] Kume Y., Takahashi T., Itakura Y., Lee S., Makizako H., Ono T., Shimada H., Ota H. (2021). Polypharmacy and lack of joy are related to physical frailty among Northern Japanese community-dwellers from the ORANGE cohort study. Gerontology.

[69] Carneiro J.A., Souza A.S., Maia L.C., Costa F.M., Moraes E.N., Caldeira A.P. (2020). Frailty in community-dwelling older people: Comparing screening instruments. Rev. Saude Publica.

[70] Lockery J.E., Ernst M.E., Broder J.C., Orchard S.G., Murray A., Nelson M.R., Stocks N.P., Wolfe R., Reid C.M., Liew D. (2020). Prescription medication use in older adults without major cardiovascular disease enrolled in the aspirin in reducing events in the elderly (ASPREE) clinical trial. Pharmacother. J. Hum. Pharmacol. Drug Ther..

[71] Alves M.K.L., Oliveira N.G.N., Pegorari M.S., Tavares D.M.d.S., Rodrigues M.C.S., Bolina A.F. (2020). Evidence of association between the use of drugs and community-dwelling older people frailty: A cross-sectional study. Sao Paulo Med. J..

[72] Braun T., Thiel C., Ziller C., Rasche J., Bahns C., Happe L., Retzmann T., Grüneberg C. (2019). Prevalence of frailty in older adults in outpatient physiotherapy in an urban region in the western part of Germany: A cross-sectional study. BMJ Open.

[73] Huang C.H., Lai Y.-C., Lee Y.C., Teong X.T., Kuzuya M., Kuo K.-M. (2018). Impact of health literacy on frailty among community-dwelling seniors. J. Clin. Med..

[74] Fhon J.R., Rodrigues R.A., Santos J.L., Diniz M.A., Santos E.B., Almeida V.C., Giacomini S.B. (2018). Factors associated with frailty in older adults: A longitudinal study. Rev. Saude Publica.

[75] Ballew S.H., Chen Y., Daya N.R., Godino J.G., Windham B.G., McAdams-DeMarco M., Coresh J., Selvin E., Grams M.E. (2017). Frailty, kidney function, and polypharmacy: The atherosclerosis risk in communities (ARIC) study. Am. J. Kidney Dis..

[76] König M., Spira D., Demuth I., Steinhagen-Thiessen E., Norman K. (2018). Polypharmacy as a risk factor for clinically relevant sarcopenia: Results from the Berlin Aging Study II. J. Gerontol. Ser. A.

[77] Closs V.E., Ziegelmann P.K., da Silva Filho I.G., Schwanke C.H.A. (2016). Frailty and geriatric syndromes in elderly assisted in primary health care. Acta Sci. Health Sci..

[78] Jankowska-Polańska B., Dudek K., Szymanska-Chabowska A., Uchmanowicz I. (2016). The influence of frailty syndrome on medication adherence among elderly patients with hypertension. Clin. Interv. Aging.

[79] Verloo H., Goulet C., Morin D., von Gunten A. (2016). Association between frailty and delirium in older adult patients discharged from hospital. Clin. Interv. Aging.

[80] Jung H.-W., Jang I.-Y., Lee Y.S., Lee C.K., Cho E.-I., Kang W.Y., Chae J.H., Lee E.J., Kim D.H. (2016). Prevalence of frailty and aging-related health conditions in older Koreans in rural communities: A cross-sectional analysis of the aging study of Pyeongchang rural area. J. Korean Med. Sci..

[81] Çakmur H. (2015). Frailty among elderly adults in a rural area of Turkey. Med. Sci. Monit. Int. Med. J. Exp. Clin. Res..

[82] Wang R., Chen L., Fan L., Gao D., Liang Z., He J., Gong W., Gao L. (2015). Incidence and effects of polypharmacy on clinical outcome among patients aged 80+: A five-year follow-up study. PLoS ONE.

[83] Gnjidic D., Hilmer S.N., Blyth F.M., Naganathan V., Cumming R.G., Handelsman D.J., McLachlan A.J., Abernethy D.R., Banks E., Le Couteur D.G. (2012). High-risk prescribing and incidence of frailty among older community-dwelling men. Clin. Pharmacol. Ther..

[84] Correa Miguel R.d.C., Dias R.C., Domingues Dias J.M., Azevedo da Silva S.L., Menicucci Filho P.R., Ribeiro T.M.S. (2012). Frailty syndrome in the community-dwelling elderly with osteoarthritis. Rev. Bras. Reumatol..

[85] Rodríguez-Laso Á., García-García F.J., Rodríguez-Mañas L. (2023). Predictors of Maintained Transitions Between Robustness and Prefrailty in Community-Dwelling Older Spaniards. J. Am. Med. Dir. Assoc..

[86] Oliveira A.D., Reiners A.A., Azevedo R.C., Silva K.M., Silva A.M. (2022). Pre-frailty in older adults: Prevalence and associated factors. Texto Contexto-Enferm..

[87] Chen Y.-Z., Huang S.-T., Wen Y.-W., Chen L.-K., Hsiao F.-Y. (2021). Combined effects of frailty and polypharmacy on health outcomes in older adults: Frailty outweighs polypharmacy. J. Am. Med. Dir. Assoc..

[88] Kim E., Sok S.R., Won C.W. (2021). Factors affecting frailty among community-dwelling older adults: A multi-group path analysis according to nutritional status. Int. J. Nurs. Stud..

[89] Lewis E.G., Whitton L.A., Collin H., Urasa S., Howorth K., Walker R.W., Dotchin C., Mulligan L., Shah B., Mohamed A. (2020). A brief frailty screening tool in Tanzania: External validation and refinement of the B-FIT screen. Aging Clin. Exp. Res..

[90] Khera S., Abbasi M., Dabravolskaj J., Sadowski C.A., Yua H., Chevalier B. (2019). Appropriateness of medications in older adults living with frailty: Impact of a pharmacist-led structured medication review process in primary care. J. Prim. Care Community Health.

[91] Tampaki M., Livada A., Fourka M.-N., Lazaridou E., Kotsani M., Benetos A., Sfikakis P.P., Kravvariti E. (2023). Inappropriate prescribing in geriatric rural primary care: Impact on adverse outcomes and relevant risk factors in a prospective observational cohort study. Aging Clin. Exp. Res..

[92] Nishtala P.S., Pickering J.W., Bergler U., Mangin D., Hilmer S.N., Jamieson H. (2023). Post Hoc Analyses of a Randomized Controlled Trial for the Effect of Pharmacist Deprescribing Intervention on the Anticholinergic Burden in Frail Community-Dwelling Older Adults. J. Am. Med. Dir. Assoc..

[93] Fravel M.A., Ernst M.E., Gilmartin-Thomas J., Woods R.L., Orchard S.G., Owen A.J., The ASPirin in Reducing Events in the Elderly Investigator Group ASPirin in Reducing Events in the Elderly Investigator Group (2023). Dietary supplement and complementary and alternative medicine use among older adults in Australia and the United States. J. Am. Geriatr. Soc..

[94] Daou T., Kharma J.A., Daccache A., Bassil M., Naja F., Rahi B. (2022). Association between Lebanese Mediterranean Diet and Frailty in Community-Dwelling Lebanese Older Adults—A Preliminary Study. Nutrients.

[95] Xu X., Zhou X., Liu W., Ma Q., Deng X., Fang R. (2022). Evaluation of the correlation between frailty and sleep quality among elderly patients with osteoporosis: A cross-sectional study. BMC Geriatr..

[96] Molist-Brunet N., Sevilla-Sánchez D., Puigoriol-Juvanteny E., Barneto-Soto M., González-Bueno J., Espaulella-Panicot J. (2022). Improving individualized prescription in patients with multimorbidity through medication review. BMC Geriatr..

[97] Thiruchelvam K., Byles J., Hasan S.S., Egan N., Kairuz T. (2021). Home Medicines Review and frailty among community-dwelling older women. Int. J. Pharm. Pract..

[98] Brown P.J., Ciarleglio A., Roose S.P., Garcia C.M., Chung S., Fernandes S., Rutherford B.R. (2021). Frailty and depression in late life: A high-risk comorbidity with distinctive clinical presentation and poor antidepressant response. J. Gerontol. Ser. A.

[99] Mejías-Trueba M., Rodríguez-Pérez A., Sotillo-Sánchez I., Sánchez-Fidalgo S., Nieto-Martin M.D., García-Cabrera E. (2023). Prevalence of potentially inappropriate medications in patients with multimorbidity according to LESS-CHRON and STOPPFrail Criteria. J. Am. Med. Dir. Assoc..

[100] Salaffi F., Di Carlo M., Carotti M., Farah S., Giovagnoni A. (2021). Frailty prevalence according to the Survey of Health, Ageing and Retirement in Europe-Frailty Instrument (SHARE-FI) definition, and its variables associated, in patients with symptomatic knee osteoarthritis: Findings from a cross-sectional study. Aging Clin. Exp. Res..

[101] Cheung J.T.K., Yu R., Woo J. (2020). Is polypharmacy beneficial or detrimental for older adults with cardiometabolic multimorbidity? Pooled analysis of studies from Hong Kong and Europe. Fam. Pract..

[102] Ambagtsheer R.C., Visvanathan R., Dent E., Yu S., Schultz T.J., Beilby J.M.D. (2020). Commonly used screening instruments to identify frailty among community-dwelling older people in a general practice (primary care) setting: A study of diagnostic test accuracy. J. Gerontol. Ser. A.

[103] Sheppard J.P., Burt J., Lown M., Temple E., Lowe R., Fraser R., Allen J., Ford G.A., Heneghan C., Hobbs F.R. (2020). Effect of antihypertensive medication reduction vs usual care on short-term blood pressure control in patients with hypertension aged 80 years and older: The OPTIMISE randomized clinical trial. JAMA.

[104] Cil G., Park J., Bergen A.W. (2019). Self-reported prescription drug use for pain and for sleep and incident frailty. J. Am. Geriatr. Soc..

[105] Reallon E., Chavent B., Gervais F., Dauphinot V., Vernaudon J., Krolak-Salmon P., Mouchoux C., Novais T. (2020). Medication exposure and frailty in older community-dwelling patients: A cross-sectional study. Pharm. Weekbl..

[106] Rieckert A., Trampisch U.S., Klaaßen-Mielke R., Drewelow E., Esmail A., Johansson T., Keller S., Kunnamo I., Löffler C., Mäkinen J. (2018). Polypharmacy in older patients with chronic diseases: A cross-sectional analysis of factors associated with excessive polypharmacy. BMC Fam. Pract..

[107] Öztürk Y., Cömertoğlu E.O., Hafızoğlu M., Kahyaoğlu Z., Çavuşoğlu Ç., Balcı C., Doğu B.B., Halil M., Aki Ö.E., Cankurtaran M. (2023). The Relationship Between Polypharmacy and Geropsychiatric Assessment Scales in Geriatric Outpatients. Eur. J. Geriatr. Gerontol..

[108] Fikree S., Hafid S., Lawson J., Agarwal G., Griffith L.E., Jaakkimainen L., Mangin D., Howard M. (2023). The association between patients’ frailty status, multimorbidity, and demographic characteristics and changes in primary care for chronic conditions during the COVID-19 pandemic: A pre-post study. Fam. Pract..

[109] Vendola M.C.C., Jacob-Filho W. (2023). Impact of oral health on frailty syndrome in frail older adults. Einstein.

[110] Badrkhahan S.Z., Ala M., Fakhrzadeh H., Yaghoobi A., Mirzamohamadi S., Arzaghi S.M., Shahabi S., Sharifi F., Ostovar A., Fahimfar N. (2023). The prevalence and predictors of geriatric giants in community-dwelling older adults: A cross-sectional study from the Middle East. Sci. Rep..

[111] Dixe M.d.A., Pinho J., Pereira F., Verloo H., Meyer-Massetti C., Pereira S.G. (2023). Patterns of medication management and associated medical and clinical features among home-dwelling older adults: A cross-sectional study in Central Portugal. Int. J. Environ. Res. Public Health.

[112] Salazar J., Borges I., Rivas-Motenegro A., Villasmil-Hernandez N., Nava M., Añez R. (2022). Association of Newly Diagnosed Hypertension and Polypharmacy with Frailty in a Tertiary Hospital Patients from Maracaibo City, Venezuela. Curr. Hypertens. Rev..

[113] Kinoshita K., Satake S., Murotani K., Takemura M., Matsui Y., Arai H. (2022). Physical frailty and hemoglobin-to-red cell distribution width ratio in Japanese older outpatients. J. Frailty Aging.

[114] Dautzenberg L., van Aarle T.T.M., Stella P.R., Emmelot-Vonk M., Weterman M.A., Koek H.L. (2022). The impact of frailty on adverse outcomes after transcatheter aortic valve replacement in older adults: A retrospective cohort study. Catheter. Cardiovasc. Interv..

[115] Ribeiro É.C.T., Sangali T.D., Clausell N.O., Perry I.S., Souza G.C. (2022). C-reactive protein and frailty in heart failure. Am. J. Cardiol..

[116] Pilleron S., Le Goff M., Ajana S., Helmer C., Pérès K., Dartigues J.-F., Tabue-Teguo M., Féart C. (2022). Self-rated health and frailty in older adults from the population-based three-city bordeaux cohort. Gerontology.

[117] Molino C.d.G.R.C., Chocano-Bedoya P.O., Sadlon A., Theiler R., Orav J.E., Vellas B., Rizzoli R., Kressig R.W., Kanis J.A., Guyonnet S. (2022). Prevalence of polypharmacy in community-dwelling older adults from seven centres in five European countries: A cross-sectional study of DO-HEALTH. BMJ Open.

[118] Álvarez-Bustos A., Carnicero-Carreño J.A., Sanchez-Sanchez J.L., Garcia-Garcia F.J., Alonso-Bouzón C., Rodríguez-Mañas L. (2022). Associations between frailty trajectories and frailty status and adverse outcomes in community-dwelling older adults. J. Cachexia Sarcopenia Muscle.

[119] O’dOnoghue P.J., Claffey P., Rice C., Byrne L., Cunningham C., Kenny R.A., Romero-Ortuno R. (2021). Association between gait speed and the SHARE Frailty Instrument in a Falls and Syncope Clinic. Eur. Geriatr. Med..

[120] Houghton J.S., Nickinson A.T., Helm J.R., Dimitrova J., Dubkova S., Rayt H.S., Gray L.J., Haunton V.J., Davies R.S., Sayers R.D. (2021). Associations of clinical frailty with severity of limb threat and outcomes in chronic limb-threatening ischaemia. Ann. Vasc. Surg..

[121] de Breij S., van Hout H.P., De Bruin S.R., Schuster N.A., Deeg D.J., Huisman M., Hoogendijk E.O. (2021). Predictors of frailty and vitality in older adults aged 75 years and over: Results from the longitudinal aging study Amsterdam. Gerontology.

[122] Jung H., Kim M., Lee Y., Won C.W. (2020). Prevalence of physical frailty and its multidimensional risk factors in Korean community-dwelling older adults: Findings from Korean frailty and aging cohort study. Int. J. Environ. Res. Public Health.

[123] Arauna D., Cerda A., Garcia-García J.F., Wehinger S., Castro F., Méndez D., Alarcón M., Fuentes E., Palomo I. (2020). Polypharmacy is associated with frailty, nutritional risk and chronic disease in chilean older adults: Remarks from piei-es study. Clin. Interv. Aging.

[124] O’COnnell J., Henman M.C., McMahon N., Burke É., McCallion P., McCarron M., O’DWyer M. (2020). Medication burden and frailty in older adults with intellectual disability: An observational cross-sectional study. Pharmacoepidemiol. Drug Saf..

[125] Doğan Varan H.A., Kilic M., Kizilarslanoğlu M., Tuna Doğrul R., Arik G., Kara O., Güner G., Şengül Ayçiçek G., Can B., Halil M. (2020). Frailty and its correlates in older adults: A challenging and preventable geriatric syndrome. Erciyes Med. J..

[126] Mino-León D., Sánchez-García S., Giraldo-Rodríguez L., Reyes-Morales H. (2019). Potentially inappropriate prescribing to older adults in ambulatory care: Prevalence and associated patient conditions. Eur. Geriatr. Med..

[127] Huguet L.G., González M.N., Kostov B., Carmona M.O., Francia C.C., Nieto M.C., Docón A.H., Sauquet R.V., Prado R.G., Sisó-Almirall A. (2018). Pre frail 80: Multifactorial intervention to prevent progression of pre-frailty to frailty in the elderly. J. Nutr. Health Aging.

[128] Hasan S.S., Liew A.S.C., Chong D.W.K., Thiruchelvam K., Babar Z.-U. (2018). Associations between Drug Burden Index, medication appropriateness and patient-reported outcomes in the community pharmacy setting in Malaysia. Drugs Ther. Perspect..

[129] Yuki A., Otsuka R., Tange C., Nishita Y., Tomida M., Ando F., Shimokata H. (2018). Polypharmacy is associated with frailty in Japanese community-dwelling older adults. Geriatr. Gerontol. Int..

[130] Rhalimi M., Rauss A., Housieaux E. (2018). Drug-related problems identified during geriatric medication review in the community pharmacy. Pharm. Weekbl..

[131] Hoeksema A., Spoorenberg S., Peters L., Meijer H., Raghoebar G., Vissink A., Wynia K., Visser A. (2017). Elderly with remaining teeth report less frailty and better quality of life than edentulous elderly: A cross-sectional study. Oral Dis..

[132] Shmuel S., Lund J.L., Alvarez C., Hsu C.D., Palta P., Kucharska-Newton A., Jordan J.M., Nelson A.E., Golightly Y.M. (2019). Polypharmacy and incident frailty in a longitudinal community-based cohort study. J. Am. Geriatr. Soc..

[133] Aznar-Tortonda V., Palazón-Bru A., la Rosa D.M.F.-D., Espínola-Morel V., Pérez-Pérez B.F., León-Ruiz A.B., Gil-Guillén V.F. (2020). Detection of frailty in older patients using a mobile app: Cross-sectional observational study in primary care. Br. J. Gen. Pract..

[134] Sanghai S., Wong C., Wang Z., Clive P., Tran W., Waring M., Goldberg R., Hayward R., Saczynski J.S., McManus D.D. (2020). Rates of potentially inappropriate dosing of direct-acting oral anticoagulants and associations with geriatric conditions among older patients with atrial fibrillation: The SAGE-AF study. J. Am. Heart Assoc..

[135] Vergara I., Mateo-Abad M., Saucedo-Figueredo M.C., Machón M., Montiel-Luque A., Vrotsou K., del Val M.A.N., Díez-Ruiz A., Güell C., Matheu A. (2019). Description of frail older people profiles according to four screening tools applied in primary care settings: A cross sectional analysis. BMC Geriatr..

[136] Lorenzo-López L., López-López R., Maseda A., Buján A., Rodríguez-Villamil J.L., Millán-Calenti J.C. (2019). Changes in frailty status in a community-dwelling cohort of older adults: The VERISAÚDE study. Maturitas.

[137] Rea F., Savaré L., Valsassina V., Ciardullo S., Perseghin G., Corrao G., Mancia G. (2023). Adherence to antidiabetic drug therapy and reduction of fatal events in elderly frail patients. Cardiovasc. Diabetol..

[138] Anh D., Nguyen T., Nguyen T., Nguyen T.V. (2022). The validity of the FRAIL scale in frailty screening among Vietnamese older people. Aging Med. Healthc..

[139] Tembo M.C., Holloway-Kew K.L., Bortolasci C.C., Brennan-Olsen S.L., Williams L.J., Kotowicz M.A., Pasco J.A. (2021). Association between serum interleukin-6 and frailty in older men: Cross-sectional data. Eur. Geriatr. Med..

[140] Hasan S.S., Burud I.A.S., Kow C.S., Rasheed M.K., Chan K.S.C., Tay P.K., Ahmed S.I. (2021). Use of potentially inappropriate medications among older outpatients and inpatients in a tertiary care hospital in Malaysia. Int. J. Clin. Pract..

[141] Thiruchelvam K., Byles J., Hasan S.S., Egan N., Cavenagh D., Kairuz T. (2021). Common combinations of medications used among oldest-old women: A population-based study over 15 years. Aging Clin. Exp. Res..

[142] Arias-Fernández L., Smith-Plaza A.M., Barrera-Castillo M., Prado-Suárez J., Lopez-Garcia E., Rodríguez-Artalejo F., Lana A. (2021). Sleep patterns and physical function in older adults attending primary health care. Fam. Pract..

[143] Novaes P.H., Da Cruz D.T., Lucchetti A.L., Leite I.C., Lucchetti G. (2017). The “iatrogenic triad”: Polypharmacy, drug–drug interactions, and potentially inappropriate medications in older adults. Int. J. Clin. Pharm..

[144] Kim M.-J., Seo S.-H., Seo A.-R., Kim B.-K., Lee G.-Y., Choi Y.-S., Kim J.-H., Kim J.-R., Kang Y.-S., Jeong B.-G. (2019). The association of perceived neighborhood walkability and environmental pollution with frailty among community-dwelling older adults in Korean rural areas: A cross-sectional study. J. Prev. Med. Public Health.

[145] Tan L.F., Lim Z.Y., Choe R., Seetharaman S., Merchant R. (2017). Screening for frailty and sarcopenia among older persons in medical outpatient clinics and its associations with healthcare burden. J. Am. Med. Dir. Assoc..

[146] Mertens B.J., Kwint H.F., Van Marum R.J., Bouvy M.L. (2018). Are multidose drug dispensing systems initiated for the appropriate patients?. Eur. J. Clin. Pharmacol..

[147] Trevisan C., Veronese N., Maggi S., Baggio G., Toffanello E.D., Zambon S., Sartori L., Musacchio E., Perissinotto E., Crepaldi G. (2017). Factors influencing transitions between frailty states in elderly adults: The Progetto Veneto Anziani Longitudinal Study. J. Am. Geriatr. Soc..

[148] Yaghi N., Yaghi C., Abifadel M., Boulos C., Feart C. (2021). Dietary patterns and risk factors of frailty in Lebanese older adults. Nutrients.

[149] Lee Y., Kim S., Kim M., Kim B., Jeong E., Shim H., Won C. (2020). A later menopausal age is associated with a lower prevalence of physical frailty in community-dwelling older adults: The Korean Frailty and Aging Cohort Study (KFACS). Arch. Gerontol. Geriatr..

[150] Romera-Liebana L., Orfila F., Segura J.M., Real J., Fabra M.L., Möller M., Lancho S., Ramirez A., Marti N., Cullell M. (2018). Effects of a primary care-based multifactorial intervention on physical and cognitive function in frail, elderly individuals: A randomized controlled trial. J. Gerontol. Ser. A.

[151] Machón M., Mateo-Abad M., Vrotsou K., Zupiria X., Güell C., Rico L., Vergara I. (2018). Dietary patterns and their relationship with frailty in functionally independent older adults. Nutrients.

[152] Buttery A.K., Busch M.A., Gaertner B., Scheidt-Nave C., Fuchs J. (2015). Prevalence and correlates of frailty among older adults: Findings from the German health interview and examination survey. BMC Geriatr..

[153] Anjum D.Z., Strange J.E., Fosbøl E., Garred C.H., Malik M.E., Andersson C., Jhund P.S., McMurray J.J.V., Petrie M.C., Kober L. (2023). Initiation of Medical Therapy for Heart Failure Patients According to Kidney Function: A Danish Nationwide Study. Clin. Epidemiol..

[154] Díez-Villanueva P., Cosín-Sales J., Roldán-Schilling V., Barrios V., Riba-Artés D., Gavín-Sebastián O. (2023). Use of direct acting oral anticoagulants in elderly patients with atrial fibrillation: A multicenter, cross-sectional study in Spain. J. Clin. Med..

[155] Islam F., Panda M., Pathak R., Agarwalla R., Singh V., Singh F. (2020). Interplay of multimorbidity and polypharmacy on a community dwelling frail elderly cohort in the peri-urban slums of Delhi, India. J. Fam. Med. Prim. Care.

[156] Panagiotakis S.H., Simos P., Basta M., Zaganas I., Perysinaki G.S., Akoumianakis I., Tziraki C., Lionis C., Vgontzas A., Boumpas D. (2022). Interactions of mediterranean diet, obesity, polypharmacy, depression and systemic inflammation with frailty status. MAEDICA–J. Clin. Med..

[157] Chao C.-T., Huang J.-W., COGENT (COhort of GEriatric Nephrology in NTUH) Study Group (2016). Geriatric syndromes are potential determinants of the medication adherence status in prevalent dialysis patients. PeerJ.

[158] van Kempen J.A.L., Melis R.J.F., Perry M., Schers H.J., Rikkert M.G.M.O. (2015). Diagnosis of frailty after a comprehensive geriatric assessment: Differences between family physicians and geriatricians. J. Am. Board Fam. Med..

[159] Strandberg T.E., Urtamo A., Kähärä J., Strandberg A.Y., Pitkälä K.H., Kautiainen H. (2018). Statin treatment is associated with a neutral effect on health-related quality of life among community-dwelling octogenarian men: The Helsinki businessmen study. J. Gerontol. Ser. A.

[160] Cheong V.-L., Mehdizadeh D., Todd O.M., Gardner P., Zaman H., Clegg A., Alldred D.P., Faisal M. (2023). The extent of anticholinergic burden across an older Welsh population living with frailty: Cross-sectional analysis of general practice records. Age Ageing.

[161] Ye L., Elstgeest L.E.M., Zhang X., Alhambra-Borrás T., Tan S.S., Raat H. (2021). Factors associated with physical, psychological and social frailty among community-dwelling older persons in Europe: A cross-sectional study of Urban Health Centres Europe (UHCE). BMC Geriatr..

[162] Salinas-Rodríguez A., Manrique-Espinoza B., Rivera-Almaraz A., Ávila-Funes J.A. (2020). Polypharmacy is associated with multiple health-related outcomes in Mexican community-dwelling older adults. Salud Pública Méx..

[163] Martins B.A., Visvanathan R., Barrie H., Huang C.H., Matsushita E., Okada K., Satake S., Uno C., Kuzuya M. (2019). Frailty prevalence using Frailty Index, associated factors and level of agreement among frailty tools in a cohort of Japanese older adults. Arch. Gerontol. Geriatr..

[164] LaCroix A.Z., Gray S.L., Aragaki A., Cochrane B.B., Newman A.B., Kooperberg C.L., Black H., Curb J.D., Greenland P., Woods N.F. (2008). Statin use and incident frailty in women aged 65 years or older: Prospective findings from the Women’s Health Initiative Observational Study. J. Gerontol. Ser. A Biol. Sci. Med. Sci..

[165] Gafoor R., Charlton J., Ravindrarajah R., Gulliford M.C. (2019). Importance of frailty for association of antipsychotic drug use with risk of fracture: Cohort study using electronic health records. J. Am. Med. Dir. Assoc..

[166] Herr M., Cesari M., Landre B., Ankri J., Vellas B., Andrieu S., MAPT/DSA Study Group (2019). Factors associated with changes of the frailty status after age 70: Findings in the MAPT study. Ann. Epidemiol..

[167] Lee D.R., Santo E.C., Lo J.C., Weintraub M.L.R., Patton M., Gordon N.P. (2018). Understanding functional and social risk characteristics of frail older adults: A cross-sectional survey study. BMC Fam. Pract..

[168] Lampela P., Taipale H., Hartikainen S. (2016). Association between anticholinergic load and frailty in community-dwelling older people. J. Am. Geriatr. Soc..

[169] Chi C.-Y., Wang J., Lee S.-Y., Chao C.-T., Hung K.-Y., Chien K.-L. (2023). The Impact of Glucose-Lowering Strategy on the Risk of Increasing Frailty Severity among 49,519 Patients with Diabetes Mellitus: A Longitudinal Cohort Study. Aging Dis..

[170] Sargent L., Zimmerman K.M., Mohammed A., Barrett M.J., Nawaz H., Wyman-Chick K., Mackiewicz M., Roman Y., Slattum P., Russell S. (2023). Low-Income Older Adults’ Vulnerability to Anticholinergic Medication-Associated Frailty. Drugs Aging.

[171] Mizuno T., Godai K., Kabayama M., Akasaka H., Kido M., Isaka M., Kubo M., Gondo Y., Ogawa M., Ikebe K. (2023). Age Group Differences in the Association Between Sleep Status and Frailty Among Community-Dwelling Older Adults: The SONIC Study. Gerontol. Geriatr. Med..

[172] Jamieson H., Nishtala P.S., Bergler H.U., Weaver S.K., Pickering J.W., Ailabouni N.J., Abey-Nesbit R., Gullery C., Deely J., Gee S.B. (2023). Deprescribing anticholinergic and sedative drugs to reduce polypharmacy in frail older adults living in the community: A randomized controlled trial. J. Gerontol. Ser. A.

[173] Tabue-Téguo M., Villeneuve R., Helene-Pelage J., Cesari M., Chovino J., Boire A., Dramé M., Simo-Tabue N., Boucaud-Maitre D. (2023). Drug storage, polypharmacy and frailty syndrome in older people: An observational study. Pan Afr. Med. J..

[174] Ekram A.S., Ryan J., Espinoza S.E., Newman A.B., Murray A.M., Orchard S.G., Fitzgerald S.M., McNeil J.J., Ernst M.E., Woods R.L. (2023). The association between frailty and dementia-free and physical disability-free survival in community-dwelling older adults. Gerontology.

[175] Alqahtani B. (2023). Number of medications and polypharmacy are associated with frailty in older adults: Results from the Midlife in the United States study. Front. Public Health.

[176] Nishimura S., Kumamaru H., Shoji S., Nakatani E., Yamamoto H., Ichihara N., Sandhu A.T., Miyachi Y., Miyata H., Kohsaka S. (2023). Frailty and subsequent adverse outcomes in older patients with atrial fibrillation treated with oral anticoagulants: The Shizuoka study. Res. Pract. Thromb. Haemost..

[177] Kravvariti E., Ntouros P.A., Vlachogiannis N.I., Pappa M., Souliotis V.L., Sfikakis P.P. (2023). Geriatric frailty is associated with oxidative stress, accumulation, and defective repair of DNA double-strand breaks independently of age and comorbidities. J. Gerontol. Ser. A.

[178] Liu P., Pan Y., Song Y., Zhou Y., Zhang W., Li X., Li J., Li Y., Ma L. (2023). Association of metformin exposure with low risks of frailty and adverse outcomes in patients with diabetes. Eur. J. Med. Res..

[179] Şentürk Durmuş N., Akın S., Goksuluk D. (2023). Prevalence of Potentially Inappropriate Medication and Frailty: A Comparison of Three Criteria in Older Turkish Adults. J. Clin. Pract. Res..

[180] Arslan M., Çakır M., Koç E.M., Sozmen K. (2023). Determinants of Frailty and Gait Speed in People Over 65 Years of Age. J. Clin. Pract. Res..

[181] Doñate-Martínez A., Alhambra-Borrás T., Durá-Ferrandis E. (2022). Frailty as a predictor of adverse outcomes among Spanish community-dwelling older adults. Int. J. Environ. Res. Public Health.

[182] Ben Chaouacha C., Al Zaabi B., Al Farsi Y., Aftab N.M. (2022). Frailty profile of geriatric patients according to the multidimensional prognostic index: A cross-sectional study in primary care in the Sultanate of Oman. Aging Med. Health.

[183] Toepfer S., König M., Spira D., Drewelies J., Kreutz R., Bolbrinker J., Demuth I. (2022). Sex differences in characteristics associated with potentially inappropriate medication use and associations with functional capacity in older participants of the Berlin Aging Study II. Gerontology.

[184] Athuraliya N., Etherton-Beer C. (2022). Health in Men Study: Is frailty a predictor of medication-related hospitalization?. QJM Int. J. Med..

[185] Sütlü S. (2022). Frailty Status and Related Factors of Elderly People Recieving Aid from the Social Assitance Foundation in Burdur Province. Turk. J. Geriatr..

[186] Thapaliya K., Harris M.L., Byles J.E. (2021). Polypharmacy trajectories among older women with and without dementia: A longitudinal cohort study. Explor. Res. Clin. Soc. Pharm..

[187] Çakmak G., Öztürk Z.A. (2021). The Relationship Between Polypharmacy and Frailty in Older Adults: Which Frailty Assessment Tool Shows the Relationship Best?. Prog. Nutr..

[188] de Breij S., Rijnhart J.J., Schuster N.A., Rietman M.L., Peters M.J., Hoogendijk E.O. (2021). Explaining the association between frailty and mortality in older adults: The mediating role of lifestyle, social, psychological, cognitive, and physical factors. Prev. Med. Rep..

[189] Hung C.-D., Yang C.-C., Lee C.-Y., Hu S.C.-S., Chen S.-C., Hung C.-H., Chuang H.-Y., Chen C.-Y., Kuo C.-H. (2021). Polypharmacy is significantly and positively associated with the frailty status assessed using the 5-item FRAIL scale, cardiovascular health phenotypic classification of frailty index, and study of osteoporotic fractures scale. J. Clin. Med..

[190] O’DOnovan M., Sezgin D., O’CAoimh R., Liew A. (2021). The relationship between frailty and diabetes: An investigation of self-rated health, depression symptoms and quality of life in the Study of Health Aging and Retirement in Europe. Arch. Gerontol. Geriatr..

[191] Setiati S., Soejono C.H., Harimurti K., Dwimartutie N., Aryana I.G.P.S., Sunarti S., Budiningsih F., Mulyana R., Dwipa L., Sudarso A. (2021). Frailty and its associated risk factors: First phase analysis of multicentre Indonesia longitudinal aging study. Front. Med..

[192] Blanco-Reina E., Aguilar-Cano L., García-Merino M.R., Ocaña-Riola R., Valdellós J., Bellido-Estévez I., Ariza-Zafra G. (2021). Assessing prevalence and factors related to frailty in community-dwelling older adults: A multinomial logistic analysis. J. Clin. Med..

[193] Kabayama M., Kamide K., Gondo Y., Masui Y., Nakagawa T., Ogawa M., Yasumoto S., Ryuno H., Akagi Y., Kiyoshige E. (2020). The association of blood pressure with physical frailty and cognitive function in community-dwelling septuagenarians, octogenarians, and nonagenarians: The SONIC study. Hypertens. Res..

[194] Elhussein L., Jödicke A.M., He Y., Delmestri A., Robinson D.E., Strauss V.Y., Prieto-Alhambra D. (2023). Characterising complex health needs and the use of preventive therapies in the older population: A population-based cohort analysis of UK primary care and hospital linked data. BMC Geriatr..

[195] Sanghai S.R., Liu W., Wang W., Rongali S., Orkaby A.R., Saczynski J.S., Rose A.J., Kapoor A., Li W., Yu H. (2022). Prevalence of frailty and associations with oral anticoagulant prescribing in atrial fibrillation. J. Gen. Intern. Med..

[196] Melo Filho J., Moreira N.B., Vojciechowski A.S., Biesek S., Bento P.C., Gomes A.R. (2020). Frailty prevalence and related factors in older adults from southern Brazil: A cross-sectional observational study. Clinics.

[197] Machón M., Mateo-Abad M., Clerencia-Sierra M., Güell C., Poblador-Pou B., Vrotsou K., Gimeno-Miguel A., Prados-Torres A., Vergara I. (2020). Multimorbidity and functional status in older people: A cluster analysis. Eur. Geriatr. Med..

[198] Shim H., Kim S., Kim M., Kim B.S., Jeong E., Lee Y.J., Won C.W. (2020). Older men living with spouse and older women living with spouse and children have lower frailty prevalence: The Korean Frailty and Aging Cohort Study (KFACS). Ann. Geriatr. Med. Res..

[199] Chumha N., Funsueb S., Kittiwachana S., Rattanapattanakul P., Lerttrakarnnon P. (2020). An artificial neural network model for assessing frailty-associated factors in the Thai population. Int. J. Environ. Res. Public Health.

[200] Oetsma S., Boonen A., Starmans M., Peeters R., van Onna M. (2020). Validation of two frailty questionnaires in older patients with rheumatoid arthritis: A cross-sectional study. Clin. Exp. Rheumatol..

[201] Tembo M.C., Holloway-Kew K.L., Sui S.X., Dunning T., Low A.C.H., Yong S.-J., Ng B.L., Brennan-Olsen S.L., Williams L.J., Kotowicz M.A. (2020). Prevalence of frailty in older men and women: Cross-sectional data from the Geelong Osteoporosis Study. Calcif. Tissue Int..

[202] Chen S., Honda T., Narazaki K., Chen T., Kishimoto H., Kumagai S. (2019). Physical frailty and risk of needing long-term care in community-dwelling older adults: A 6-year prospective study in Japan. J. Nutr. Health Aging.

[203] Moulis F., Moulis G., Balardy L., Gérard S., Sourdet S., Rougé-Bugat M.-E., Lapeyre-Mestre M., Montastruc J.-L., Rolland Y., Vellas B. (2015). Searching for a polypharmacy threshold associated with frailty. J. Am. Med. Dir. Assoc..

[204] Serra-Prat M., Papiol M., Vico J., Palomera E., Sist X., Cabré M. (2016). Factors associated with frailty in community-dwelling elderly population. A cross-sectional study. Eur. Geriatr. Med..

[205] Meid A.D., Quinzler R., Freigofas J., Saum K.-U., Schöttker B., Holleczek B., Heider D., König H.-H., Brenner H., Haefeli W.E. (2015). Medication underuse in aging outpatients with cardiovascular disease: Prevalence, determinants, and outcomes in a prospective cohort study. PLoS ONE.

[206] Eyigor S., Kutsal Y.G., Duran E., Huner B., Paker N., Durmus B., Sahin N., Civelek G.M., Gokkaya K., Doğan A. (2015). Frailty prevalence and related factors in the older adult—FrailTURK Project. AGE.

[207] Peel N.M., Runganga M., Hubbard R. (2014). Multiple medication use in older patients in post-acute transitional care: A prospective cohort study. Clin. Interv. Aging.

[208] Koponen M.P., Bell J.S., Karttunen N.M., Nykänen I.A., Desplenter F.A., Hartikainen S.A. (2013). Analgesic use and frailty among community-dwelling older people: A population-based study. Drugs Aging.

[209] Jazbar J., Pišek Š., Locatelli I., Kos M. (2021). Prevalence and incidence of frailty among community-dwelling older adults in Slovenia. Slov. J. Public Health.

[210] Carneiro J.A., Lima C.D.A., da Costa F.M., Caldeira A.P. (2019). Health care are associated with worsening of frailty in community older adults. Rev. Saude Publica.

[211] Porter B., Arthur A., Savva G.M. (2019). How do potentially inappropriate medications and polypharmacy affect mortality in frail and non-frail cognitively impaired older adults? A cohort study. BMJ Open.

[212] Nguyen A.T., Nguyen L.H., Nguyen T.X., Nguyen T.T.H., Nguyen H.T.T., Nguyen T.N., Pham H.Q., Tran B.X., Latkin C.A., Ho C.S.H. (2019). Frailty prevalence and association with health-related quality of life impairment among rural community-dwelling older adults in Vietnam. Int. J. Environ. Res. Public Health.

[213] Panagiotakis S.H., Simos P., Zaganas I., Basta M., Perysinaki G.S., Fountoulakis N., Giaka M., Kapetanaki S., Koutentaki I., Bertsias A. (2018). Self-reported fatigue as a risk index for dementia diagnosis. Eur. Geriatr. Med..

[214] Chen P.-J., Yang K.-Y., Perng W.-C., Lin K.-C., Wang K.-Y. (2018). Effect of dyspnea on frailty stages and related factors in Taiwanese men with COPD. Int. J. Chronic Obstr. Pulm. Dis..

[215] Takeuchi H., Uchida H.A., Kakio Y., Okuyama Y., Okuyama M., Umebayashi R., Wada K., Sugiyama H., Sugimoto K., Rakugi H. (2018). The prevalence of frailty and its associated factors in Japanese hemodialysis patients. Aging Dis..

[216] Wong S., Katz A., Williamson T., Singer A., Peterson S., Taylor C., Price M., McCracken R., Thandi M. (2020). Can linked electronic medical record and administrative data help us identify those living with frailty?. Int. J. Popul. Data Sci..

[217] Herr M., Robine J.-M., Pinot J., Arvieu J.-J., Ankri J. (2015). Polypharmacy and frailty: Prevalence, relationship, and impact on mortality in a French sample of 2350 old people. Pharmacoepidemiol. Drug Saf..

[218] Meng L., Shi H., Shi J., Yu P.-L., Xi H. (2019). Differences in clinical characteristics, muscle mass, and physical performance among different frailty levels in Chinese older men. Chin. Med. J..

[219] Herr M., Sirven N., Grondin H., Pichetti S., Sermet C. (2017). Frailty, polypharmacy, and potentially inappropriate medications in old people: Findings in a representative sample of the French population. Eur. J. Clin. Pharmacol..

[220] Merchant R.A., Chen M.Z., Tan L.W.L., Lim M.Y., Ho H.K., van Dam R.M. (2017). Singapore healthy older people everyday (HOPE) study: Prevalence of frailty and associated factors in older adults. J. Am. Med. Dir. Assoc..

[221] Pao Y.-C., Chen C.-Y., Chang C.-I., Chen C.-Y., Tsai J.-S. (2018). Self-reported exhaustion, physical activity, and grip strength predict frailty transitions in older outpatients with chronic diseases. Medicine.

[222] Gnjidic D., Hilmer S.N., Blyth F.M., Naganathan V., Waite L., Seibel M.J., McLachlan A.J., Cumming R.G., Handelsman D.J., Le Couteur D.G. (2012). Polypharmacy cutoff and outcomes: Five or more medicines were used to identify community-dwelling older men at risk of different adverse outcomes. J. Clin. Epidemiol..

[223] Lakey S.L., LaCroix A.Z., Gray S.L., Borson S., Williams C.D., Calhoun D., Goveas J.S., Smoller J.W., Ockene J.K., Masaki K.H. (2012). Antidepressant use, depressive symptoms, and incident frailty in women aged 65 and older from the w omen’s h ealth i nitiative observational study. J. Am. Geriatr. Soc..

[224] Crentsil V., Ricks M.O., Xue Q.-L., Fried L.P. (2010). A pharmacoepidemiologic study of community-dwelling, disabled older women: Factors associated with medication use. Am. J. Geriatr. Pharmacother..

[225] Pérez-Ros P., Vila-Candel R., López-Hernández L., Martínez-Arnau F.M. (2020). Nutritional status and risk factors for frailty in community-dwelling older people: A cross-sectional study. Nutrients.

[226] García-Esquinas E., Graciani A., Guallar-Castillón P., López-García E., Rodríguez-Mañas L., Rodríguez-Artalejo F. (2015). Diabetes and risk of frailty and its potential mechanisms: A prospective cohort study of older adults. J. Am. Med. Dir. Assoc..

[227] Byrne C.J., Walsh C., Cahir C., Bennett K. (2019). Impact of drug burden index on adverse health outcomes in Irish community-dwelling older people: A cohort study. BMC Geriatr..

[228] Saeidimehr S., Delbari A., Zanjari N., Vatan R.F. (2021). Factors related to frailty among older adults in Khuzestan, Iran. Salmand.

[229] Güngör Başaran A.Y., Akal Yıldız E. (2022). Nutrition status, muscle mass, and frailty in older people: A cross-sectional study conducted in Cyprus. J. Am. Nutr. Assoc..

[230] Chen C.-Y., Wu S.-C., Chen L.-J., Lue B.-H. (2010). The prevalence of subjective frailty and factors associated with frailty in Taiwan. Arch. Gerontol. Geriatr..

[231] Chang C.-I., Chan D.-C., Kuo K.-N., Hsiung C.A., Chen C.-Y. (2011). Prevalence and correlates of geriatric frailty in a northern Taiwan community. J. Formos. Med. Assoc..

[232] Woo J., Leung J. (2014). Multi-morbidity, dependency, and frailty singly or in combination have different impact on health outcomes. AGE.

[233] Moulis F., Moulis G., Balardy L., Gérard S., Montastruc F., Sourdet S., Rougé-Bugat M.-E., Lapeyre-Mestre M., Montastruc J.-L., Rolland Y. (2015). Exposure to atropinic drugs and frailty status. J. Am. Med. Dir. Assoc..

[234] Woo J., Yu R., Wong M., Yeung F., Wong M., Lum C. (2015). Frailty screening in the community using the FRAIL scale. J. Am. Med. Dir. Assoc..

[235] Ravindrarajah R., Dregan A., Hazra N.C., Hamada S., Jackson S.H., Gulliford M.C. (2017). Declining blood pressure and intensification of blood pressure management among people over 80 years: Cohort study using electronic health records. J. Hypertens..

[236] Woo J., Zheng Z., Leung J., Chan P. (2015). Prevalence of frailty and contributory factors in three Chinese populations with different socioeconomic and healthcare characteristics. BMC Geriatr..

[237] Thompson M.Q., Theou O., Yu S., Adams R.J., Tucker G.R., Visvanathan R. (2018). Frailty prevalence and factors associated with the frailty phenotype and frailty index: Findings from the north West Adelaide health study. Australas. J. Ageing.

[238] Kleipool E.E.F., Nielen M.M.J., Korevaar J.C., Harskamp R.E., Smulders Y.M., Serné E., Thijs A., Peters M.J.L., Muller M. (2019). Prescription patterns of lipid lowering agents among older patients in general practice: An analysis from a national database in the Netherlands. Age Ageing.

[239] Tabue-Teguo M., Grasset L., Avila-Funes J.A., Genuer R., Proust-Lima C., Péres K., Féart C., Amieva H., Harmand M.G.-C., Helmer C. (2018). Prevalence and co-occurrence of geriatric syndromes in people aged 75 years and older in France: Results from the Bordeaux three-city study. J. Gerontol. Ser. A.

[240] Hironaka S., Kugimiya Y., Watanabe Y., Motokawa K., Hirano H., Kawai H., Kera T., Kojima M., Fujiwara Y., Ihara K. (2020). Association between oral, social, and physical frailty in community-dwelling older adults. Arch. Gerontol. Geriatr..

[241] Threapleton C.J., Kimpton J.E., Carey I.M., DeWilde S., Cook D.G., Harris T., Baker E.H. (2020). Development of a structured clinical pharmacology review for specialist support for management of complex polypharmacy in primary care. Br. J. Clin. Pharmacol..

[242] Taci D.Y., Yılmaz S., Arslan I., Fidancı İ., Çelik M. (2023). The evaluation of frailty in the elderly and affecting biopsychosocial factors: A cross-sectional observational study. Iran. J. Public Health.

[243] Erdoğan I., Tuncer O. (2023). Evaluation of the Relationship Between Frailty, Polypharmacy, and Depression in People 65 Years of Age and Older. Ank. Med. J..

[244] Sánchez-García S., Gallegos-Carrillo K., Espinel-Bermudez M.C., Doubova S.V., Sánchez-Arenas R., García-Peña C., Salvà A., Briseño-Fabian S.C. (2017). Comparison of quality of life among community-dwelling older adults with the frailty phenotype. Qual. Life Res..

[245] Kleipool E.E., Hoogendijk E.O., Trappenburg M.C., Handoko M.L., Huisman M., Peters M.J., Muller M. (2018). Frailty in older adults with cardiovascular disease: Cause, effect or both?. Aging Dis..

[246] Thein F.S., Li Y., Nyunt M.S.Z., Gao Q., Wee S.L., Ng T.P. (2018). Physical frailty and cognitive impairment is associated with diabetes and adversely impact functional status and mortality. Postgrad. Med..

[247] Esenkaya M.E., Dokuzlar O., Soysal P., Smith L., Jackson S.E., Isik A.T. (2019). Validity of the Kihon Checklist for evaluating frailty status in Turkish older adults. Geriatr. Gerontol. Int..

[248] Qiao X., Tian X., Liu N., Dong L., Jin Y., Si H., Liu X., Wang C. (2020). The association between frailty and medication adherence among community-dwelling older adults with chronic diseases: Medication beliefs acting as mediators. Patient Educ. Couns..

[249] Okui N., Okui M. (2023). Ninjin’yoeito improves genitourinary symptoms in patients with frailty. Cureus.

[250] Hoogendijk E.O., van der Horst H.E., Deeg D.J.H., Frijters D.H.M., Prins B.A.H., Jansen A.P.D., Nijpels G., van Hout H.P.J. (2013). The identification of frail older adults in primary care: Comparing the accuracy of five simple instruments. Age Ageing.

[251] Prieto-Contreras L., Martínez-Arnau F.M., Sancho-Cantus D., Cubero-Plazas L., Pérez-Ros P. (2023). Fear of falling score is a predictor of falls in community-dwelling pre-frail and frail older people. Healthcare.

[252] Jung H.-W., Yoo H.-J., Park S.-Y., Kim S.-W., Choi J.-Y., Yoon S.-J., Kim C.-H., Kim K.-I. (2016). The Korean version of the FRAIL scale: Clinical feasibility and validity of assessing the frailty status of Korean elderly. Korean J. Intern. Med..

[253] Jung H.-W., Kim S., Won C.W. (2021). Validation of the Korean Frailty Index in community-dwelling older adults in a nationwide Korean Frailty and Aging Cohort study. Korean J. Intern. Med..

[254] Ambrož M., de Vries S.T., Sidorenkov G., Hoogenberg K., Denig P. (2020). Changes in blood pressure thresholds for initiating antihypertensive medication in patients with diabetes: A repeated cross-sectional study focusing on the impact of age and frailty. BMJ Open.

[255] Pel-Littel R.E., Schuurmans M.J., Emmelot-Vonk M.H., Verhaar H.J.J. (2009). Frailty: Defining and measuring of a concept. J. Nutr. Health Aging.

[256] van Kan G.A., Rolland Y., Houles M., Gillette-Guyonnet S., Soto M., Vellas B. (2010). The assessment of frailty in older adults. Clin. Geriatr. Med..

[257] Bonaga B., Sánchez-Jurado P.M., Martínez-Reig M., Ariza G., Rodríguez-Mañas L., Gnjidic D., Salvador T., Abizanda P. (2018). Frailty, polypharmacy, and health outcomes in older adults: The frailty and dependence in albacete study. J. Am. Med. Dir. Assoc..

[258] Curtin D., Gallagher P.F., O’mahony D. (2019). Explicit criteria as clinical tools to minimize inappropriate medication use and its consequences. Ther. Adv. Drug Saf..

[259] Ma W., Wang H., Wen Z., Liu L., Zhang X. (2023). Potentially inappropriate medication and frailty in older adults: A systematic review and meta-analysis. Arch. Gerontol. Geriatr..

[260] Randles M.A., O’mahony D., Gallagher P.F. (2022). Frailty and potentially inappropriate prescribing in older people with polypharmacy: A bi-directional relationship?. Drugs Aging.

