# Supplementary material for: Medication Use by Older Adults with Frailty: A Scoping Review
Source: Pharmacy (Basel). 2025 Nov 21;13(6):170. doi: 10.3390/pharmacy13060170 (PMC12641866; doi:10.3390/pharmacy13060170)
Supplement: Supplementary file 1 [file pharmacy-13-00170-s001.zip › pharmacy-3960381-supplementary.pdf]

Supplementary Table S1. Preferred Reporting Items for Systematic reviews and Meta-Analyses extension for Scoping Reviews (PRISMA-ScR) Checklist

SECTION	ITEM	PRISMA-ScR CHECKLIST ITEM	REPORTED ON PAGE #
**TITLE**			
Title	1	Identify the report as a scoping review.	Page 1; Line1-2
**ABSTRACT**			
Structured summary	2	Provide a structured summary that includes (as applicable): background, objectives, eligibility criteria, sources of evidence, charting methods, results, and conclusions that relate to the review questions and objectives.	Page 1; Line 10- 30
**INTRODUCTION**			
Rationale	3	Describe the rationale for the review in the context of what is already known. Explain why the review questions/objectives lend themselves to a scoping review approach.	Page1,2; Line 34- 82
Objectives	4	Provide an explicit statement of the questions and objectives being addressed with reference to their key elements (e.g., population or participants, concepts, and context) or other relevant key elements used to conceptualize the review questions and/or objectives.	Page 2; Line 83- 90
**METHODS**			
Protocol and registration	5	Indicate whether a review protocol exists; state if and where it can be accessed (e.g., a Web address); and if available, provide registration information, including the registration number.	Not registered
Eligibility criteria	6	Specify characteristics of the sources of evidence used as eligibility criteria (e.g., years considered, language, and publication status), and provide a rationale.	Page 3; Line 107- 121
Information sources*	7	Describe all information sources in the search (e.g., databases with dates of coverage and contact with authors to identify additional sources), as well as the date the most recent search was executed.	Page 3; Line 108- 110
Search	8	Present the full electronic search strategy for at least 1 database, including any limits used, such that it could be repeated.	Supplementary table 1
Selection of sources of evidence†	9	State the process for selecting sources of evidence (i.e., screening and eligibility) included in the scoping review.	Page 3; Line 123- 138
Data charting process‡	10	Describe the methods of charting data from the included sources of evidence (e.g., calibrated forms or forms that have been tested by the team before their use, and whether data charting was done independently or in duplicate) and any processes for obtaining and confirming data from investigators.	Page 4; Line 140- 152
Data items	11	List and define all variables for which data were sought and any assumptions and simplifications made.	Page 4; Line 154- 165

SECTION	ITEM	PRISMA-ScR CHECKLIST ITEM	REPORTED ON PAGE #
Critical appraisal of individual sources of evidence§	12	If done, provide a rationale for conducting a critical appraisal of included sources of evidence; describe the methods used and how this information was used in any data synthesis (if appropriate).	Not done
Synthesis of results	13	Describe the methods of handling and summarizing the data that were charted.	Page 4; Line 153- 165
**RESULTS**			
Selection of sources of evidence	14	Give numbers of sources of evidence screened, assessed for eligibility, and included in the review, with reasons for exclusions at each stage, ideally using a flow diagram.	Page 4; Line 166- 172
Characteristics of sources of evidence	15	For each source of evidence, present characteristics for which data were charted and provide the citations.	Page 4,5; Line 173- 222
Critical appraisal within sources of evidence	16	If done, present data on critical appraisal of included sources of evidence (see item 12).	Not done
Results of individual sources of evidence	17	For each included source of evidence, present the relevant data that were charted that relate to the review questions and objectives.	Page 5- 17; Line 223- 558
Synthesis of results	18	Summarize and/or present the charting results as they relate to the review questions and objectives.	Page 5- 17; Line 223- 558
**DISCUSSION**			
Summary of evidence	19	Summarize the main results (including an overview of concepts, themes, and types of evidence available), link to the review questions and objectives, and consider the relevance to key groups.	Page 17- 20; Line 559- 707
Limitations	20	Discuss the limitations of the scoping review process.	Page 20; Line 709- 734
Conclusions	21	Provide a general interpretation of the results with respect to the review questions and objectives, as well as potential implications and/or next steps.	Page 20; Line 735- 743
**FUNDING**			
Funding	22	Describe sources of funding for the included sources of evidence, as well as sources of funding for the scoping review. Describe the role of the funders of the scoping review.	Page 20; Line 750

JBI = Joanna Briggs Institute; PRISMA-ScR = Preferred Reporting Items for Systematic reviews and Meta-Analyses extension for Scoping Reviews.

* Where *sources of evidence* (see second footnote) are compiled from, such as bibliographic databases, social media platforms, and Web sites.

† A more inclusive/heterogeneous term used to account for the different types of evidence or data sources (e.g., quantitative and/or qualitative research, expert opinion, and policy documents) that may be eligible in a scoping review as opposed to only studies. This is not to be confused with *information sources* (see first footnote).

‡ The frameworks by Arksey and O'Malley (6) and Levac and colleagues (7) and the JBI guidance (4, 5) refer to the process of data extraction in a scoping review as data charting.

§ The process of systematically examining research evidence to assess its validity, results, and relevance before using it to inform a decision. This term is used for items 12 and 19 instead of "risk of bias" (which is more applicable to systematic reviews of interventions) to include and acknowledge the various sources of evidence that may be used in a scoping review (e.g., quantitative and/or qualitative research, expert opinion, and policy document).

From: Tricco AC, Lillie E, Zarin W, O'Brien KK, Colquhoun H, Levac D, et al. PRISMA Extension for Scoping Reviews (PRISMAScR): Checklist and Explanation. Ann Intern Med. 2018;169:467–473. doi: 10.7326/M18-0850.

Supplementary Table S2. Search Strategies

**PubMed(MEDLINE):**
(frailty[mesh] OR sarcopenia[mesh] OR frail*[tiab] OR sarcopeni*[tiab] OR sarcopaeni*[tiab] OR weak*[tiab] OR feeble[tiab] OR debilit*[tiab]) **AND** (aged[mesh] OR nursing homes[mesh] OR homes for the aged[mesh] OR aged[tiab] OR “older adult*”[tiab] OR “older person*”[tiab] OR “older people*”[tiab] OR “older generation”[tiab] OR “older individual*”[tiab] OR “older patient*”[tiab] OR “older man”[tiab] OR “older men”[tiab] OR “older woman”[tiab] OR “older women”[tiab] OR “old age”[tiab] OR elder*[tiab] OR geriatric[tiab] OR “senior citizen*”[tiab] OR retiree*[tiab] OR septuagenarian*[tiab] OR octagenarian*[tiab] OR octogenarian*[tiab] OR nonagenarian*[tiab] OR centenarian*[tiab] OR “nursing home*”[tiab]) **AND** (medication errors[mesh] OR medication therapy management[mesh] OR deprescriptions[mesh] OR potentially inappropriate medication list[mesh] OR polypharmacy[mesh] OR drug monitoring[mesh] OR medication review[mesh] OR “medication error*”[tiab] OR “medication administration error*”[tiab] OR “drug error*”[tiab] OR “drug administration error*”[tiab] OR “prescription error*”[tiab] OR “prescription administration error*”[tiab] OR “erroneous medication administration”[tiab] OR “erroneous drug administration”[tiab] OR “erroneous prescription administration”[tiab] OR “wrong medication*”[tiab] OR “wrong drug*”[tiab] OR “wrong prescription*”[tiab] OR “medication reconciliation”[tiab] OR “drug reconciliation”[tiab] OR “prescription reconciliation”[tiab] OR “medication therapy management”[tiab:~3] OR “medication management”[tiab:~3] OR “manage medication”[tiab:~3] OR “manage medications”[tiab:~3] OR “managing medication”[tiab:~3] OR “managing medications”[tiab:~3] OR “managed medication”[tiab:~3] OR “managed medications”[tiab:~3] OR “drug therapy management”[tiab:~3] OR “drug management”[tiab:~3] OR “management drugs”[tiab:~3] OR “manage drugs”[tiab:~3] OR “managing drugs”[tiab:~3] OR “managed drugs”[tiab:~3] OR “prescription management”[tiab:~3] OR “manage prescriptions”[tiab:~3] OR “managing prescriptions”[tiab:~3] OR “managed prescriptions”[tiab:~3] OR “medication use”[tiab:~3] OR “medications use”[tiab:~3] OR “prescription use”[tiab:~3] OR “prescriptions use”[tiab:~3] OR “monitor medication”[tiab:~3] OR “monitor medications”[tiab:~3] OR “monitoring medication”[tiab:~3] OR “monitoring medications”[tiab:~3] OR “monitored medication”[tiab:~3] OR “monitored medications”[tiab:~3] OR “monitor drugs”[tiab:~3] OR “drug monitoring”[tiab:~3] OR “monitoring drugs”[tiab:~3] OR “monitored drugs”[tiab:~3] OR “monitor prescriptions”[tiab:~3] OR “prescription monitoring”[tiab:~3] OR “monitored prescriptions”[tiab:~3] OR “discontinue medication”[tiab:~3] OR “discontinue medications”[tiab:~3] OR “discontinuing medication”[tiab:~3] OR “discontinuing medications”[tiab:~3] OR “discontinued medication”[tiab:~3] OR “discontinued medications”[tiab:~3] OR “discontinue drug”[tiab:~3] OR “discontinue drugs”[tiab:~3] OR “discontinuing drug”[tiab:~3] OR “discontinuing drugs”[tiab:~3] OR “discontinued drug”[tiab:~3] OR “discontinued drugs”[tiab:~3] OR “discontinue prescription”[tiab:~3] OR “discontinue prescriptions”[tiab:~3] OR “discontinuing prescription”[tiab:~3] OR “discontinuing prescriptions”[tiab:~3] OR “discontinued prescription”[tiab:~3] OR “discontinued prescriptions”[tiab:~3] OR “taper medication”[tiab:~3] OR “taper medications”[tiab:~3] OR “tapering medication”[tiab:~3] OR “tapering medications”[tiab:~3] OR “tapered medication”[tiab:~3] OR “tapered medications”[tiab:~3] OR “taper drug”[tiab:~3] OR “taper drugs”[tiab:~3] OR “tapering drug”[tiab:~3] OR “tapering drugs”[tiab:~3] OR “tapered drug”[tiab:~3] OR “tapered drugs”[tiab:~3] OR “taper prescription”[tiab:~3] OR “taper prescriptions”[tiab:~3] OR “tapering prescription”[tiab:~3] OR “tapering prescriptions”[tiab:~3] OR “tapered prescription”[tiab:~3] OR “tapered prescriptions”[tiab:~3] OR “stop medication”[tiab:~3] OR “stop medications”[tiab:~3] OR “stopping medication”[tiab:~3] OR “stopping medications”[tiab:~3] OR “stopped medication”[tiab:~3] OR “stopped medications”[tiab:~3] OR “stop drug”[tiab:~3] OR “stop drugs”[tiab:~3] OR “stopping drug”[tiab:~3] OR “stopping drugs”[tiab:~3] OR “stopped drug”[tiab:~3] OR “stopped

drugs”[tiab:~3] OR “stop prescription”[tiab:~3] OR “stop prescriptions”[tiab:~3] OR “stopping prescription”[tiab:~3] OR “stopping prescriptions”[tiab:~3] OR “stopped prescription”[tiab:~3] OR “stopped prescriptions”[tiab:~3] OR “reduce medication”[tiab:~3] OR “reduce medications”[tiab:~3] OR “reducing medication”[tiab:~3] OR “reducing medications”[tiab:~3] OR “reduced medication”[tiab:~3] OR “reduced medications”[tiab:~3] OR “reduce drug”[tiab:~3] OR “reduce drugs”[tiab:~3] OR “reducing drugs”[tiab:~3] OR “reduced drugs”[tiab:~3] OR “reduce prescription”[tiab:~3] OR “reduce prescriptions”[tiab:~3] OR “reducing prescriptions”[tiab:~3] OR “reduced prescription”[tiab:~3] OR “reduced prescriptions”[tiab:~3] OR “cease medication”[tiab:~3] OR “cease medications”[tiab:~3] OR “ceasing medication”[tiab:~3] OR “ceasing medications”[tiab:~3] OR “ceased medication”[tiab:~3] OR “ceased medications”[tiab:~3] OR “cease drug”[tiab:~3] OR “cease drugs”[tiab:~3] OR “ceasing drug”[tiab:~3] OR “ceasing drugs”[tiab:~3] OR “ceased drug”[tiab:~3] OR “ceased drugs”[tiab:~3] OR “cease prescription”[tiab:~3] OR “cease prescriptions”[tiab:~3] OR “ceasing prescription”[tiab:~3] OR “ceasing prescriptions”[tiab:~3] OR “ceased prescription”[tiab:~3] OR “ceased prescriptions”[tiab:~3] OR “decrease medication”[tiab:~3] OR “decrease medications”[tiab:~3] OR “decreasing medication”[tiab:~3] OR “decreasing medications”[tiab:~3] OR “decreased medication”[tiab:~3] OR “decreased medications”[tiab:~3] OR “decrease drug”[tiab:~3] OR “decrease drugs”[tiab:~3] OR “decreasing drug”[tiab:~3] OR “decreasing drugs”[tiab:~3] OR “decreased drugs”[tiab:~3] OR “decrease prescription”[tiab:~3] OR “decrease prescriptions”[tiab:~3] OR “decreasing prescription”[tiab:~3] OR “decreasing prescriptions”[tiab:~3] OR “decreased prescription”[tiab:~3] OR “decreased prescriptions”[tiab:~3] OR “review medication”[tiab:~3] OR “review medications”[tiab:~3] OR “reviewing medication”[tiab:~3] OR “reviewing medications”[tiab:~3] OR “reviewed medication”[tiab:~3] OR “reviewed medications”[tiab:~3] OR “review drug”[tiab:~3] OR “review drugs”[tiab:~3] OR “reviewing drug”[tiab:~3] OR “reviewing drugs”[tiab:~3] OR “reviewed drug”[tiab:~3] OR “reviewed drugs”[tiab:~3] OR “review prescription”[tiab:~3] OR “review prescriptions”[tiab:~3] OR “reviewing prescription”[tiab:~3] OR “reviewing prescriptions”[tiab:~3] OR “reviewed prescription”[tiab:~3] OR “reviewed prescriptions”[tiab:~3] OR “titrate medication”[tiab:~3] OR “titrate medications”[tiab:~3] OR “titrating medication”[tiab:~3] OR “titrating medications”[tiab:~3] OR “titrated medication”[tiab:~3] OR “titrated medications”[tiab:~3] OR “titrate drug”[tiab:~3] OR “titrate drugs”[tiab:~3] OR “titrating drug”[tiab:~3] OR “titrating drugs”[tiab:~3] OR “titrated drug”[tiab:~3] OR “titrated drugs”[tiab:~3] OR “titrate prescription”[tiab:~3] OR “titrate prescriptions”[tiab:~3] OR “titrating prescription”[tiab:~3] OR “titrating prescriptions”[tiab:~3] OR “titrated prescription”[tiab:~3] OR “titrated prescriptions”[tiab:~3] OR “start medication”[tiab:~3] OR “start medications”[tiab:~3] OR “starting medication”[tiab:~3] OR “starting medications”[tiab:~3] OR “started medication”[tiab:~3] OR “started medications”[tiab:~3] OR “start drug”[tiab:~3] OR “start drugs”[tiab:~3] OR “starting drug”[tiab:~3] OR “starting drugs”[tiab:~3] OR “started drug”[tiab:~3] OR “started drugs”[tiab:~3] OR “start prescription”[tiab:~3] OR “start prescriptions”[tiab:~3] OR “starting prescription”[tiab:~3] OR “starting prescriptions”[tiab:~3] OR “started prescription”[tiab:~3] OR “started prescriptions”[tiab:~3] OR “initiate medication”[tiab:~3] OR “initiate medications”[tiab:~3] OR “initiating medication”[tiab:~3] OR “initiating medications”[tiab:~3] OR “initiated medication”[tiab:~3] OR “initiated medications”[tiab:~3] OR “initiate drug”[tiab:~3] OR “initiate drugs”[tiab:~3] OR “initiating drug”[tiab:~3] OR “initiating drugs”[tiab:~3] OR “initiated drug”[tiab:~3] OR “initiated drugs”[tiab:~3] OR “initiate prescription”[tiab:~3] OR “initiate prescriptions”[tiab:~3] OR “initiating prescription”[tiab:~3] OR “initiating prescriptions”[tiab:~3] OR “initiated prescription”[tiab:~3] OR “initiated prescriptions”[tiab:~3] OR “adjust medication”[tiab:~3] OR “adjust medications”[tiab:~3] OR “adjusting medication”[tiab:~3] OR “adjusting medications”[tiab:~3] OR “adjusted medication”[tiab:~3] OR “adjusted medications”[tiab:~3] OR “adjust drug”[tiab:~3] OR “adjust drugs”[tiab:~3] OR “adjusting drug”[tiab:~3] OR “adjusting drugs”[tiab:~3] OR “adjusted drug”[tiab:~3] OR “adjusted drugs”[tiab:~3] OR

“adjust prescription”[tiab:~3] OR “adjust prescriptions”[tiab:~3] OR “adjusting prescription”[tiab:~3] OR “adjusting prescriptions”[tiab:~3] OR “adjusted prescription”[tiab:~3] OR “adjusted prescriptions”[tiab:~3] OR “switch medication”[tiab:~3] OR “switch medications”[tiab:~3] OR “switching medication”[tiab:~3] OR “switching medications”[tiab:~3] OR “switched medication”[tiab:~3] OR “switched medications”[tiab:~3] OR “switch drug”[tiab:~3] OR “switch drugs”[tiab:~3] OR “switching drug”[tiab:~3] OR “switching drugs”[tiab:~3] OR “switched drug”[tiab:~3] OR “switched drugs”[tiab:~3] OR “switch prescription”[tiab:~3] OR “switch prescriptions”[tiab:~3] OR “switching prescription”[tiab:~3] OR “switching prescriptions”[tiab:~3] OR “switched prescription”[tiab:~3] OR “switched prescriptions”[tiab:~3] OR “change medication”[tiab:~3] OR “change medications”[tiab:~3] OR “changing medication”[tiab:~3] OR “changing medications”[tiab:~3] OR “changed medication”[tiab:~3] OR “changed medications”[tiab:~3] OR “change drug”[tiab:~3] OR “change drugs”[tiab:~3] OR “changing drug”[tiab:~3] OR “changing drugs”[tiab:~3] OR “changed drug”[tiab:~3] OR “changed drugs”[tiab:~3] OR “change prescription”[tiab:~3] OR “change prescriptions”[tiab:~3] OR “changing prescription”[tiab:~3] OR “changing prescriptions”[tiab:~3] OR “changed prescription”[tiab:~3] OR “changed prescriptions”[tiab:~3] OR deprescrib*[tiab] OR deprescription*[tiab] OR “de-prescrib*”[tiab] OR “de-prescription*”[tiab] OR “over-prescrib*”[tiab] OR overprescrib*[tiab] OR “potentially inappropriate medication*”[tiab] OR “potentially inappropriate drug*”[tiab] OR “potentially inappropriate prescription*”[tiab] OR “potentially inappropriate prescribing”[tiab] OR PIM[tiab] OR PIMS[tiab] OR polypharmacy[tiab] OR “multi-drug therapy”[tiab] OR “multidrug therapy”[tiab] OR polymedication[tiab] OR “medication regimen”[tiab] OR “drug regimen”[tiab] OR “prescription regimen”[tiab]) **AND** English[lang]
Ovid Embase:<br><br>1 exp frail elderly/ 12326<br>2 exp sarcopenia/21244<br>3 (frail* or sarcopeni* or sarcopaeni* or weak* or feeble or debilit*).ti,ab. 700471<br>4 1 or 2 or 3 707292<br>5 exp aged/ 3663323<br>6 exp nursing home/ 62176<br>7 exp homes for the aged/ 11577<br>8 (aged or "older adult*" or "older person*" or "older people*" or "older generation" or "older individual*" or "older patient*" or "older man" or "older men" or "older woman" or "older women" or "old age" or elder* or geriatric or "senior citizen*" or retiree* or septuagenarian* or octagenarian* or octogenarian* or nonagenarian* or centenarian* or "nursing home*").ti,ab. 1641790<br>9 5 or 6 or 7 or 8 4590557<br>10 exp medication error/ 23757<br>11 exp medication therapy management/ 15170<br>12 exp deprescription/ 1775<br>13 exp potentially inappropriate medication/ 2761<br>14 exp polypharmacy/ 24160

15 exp drug monitoring/ 61000
16 exp "drug utilization review"/ 12481
17 ((error* or erroneous or wrong or reconciliation) adj3 (medication or drug or prescription)).ti,ab. 18535
18 ((manag* or monitor* or discontinu* or taper* or stop* or reduc* or ceas* or decreas* or review* or titrat* or start* or initiat* or adjust* or switch* or change*) adj3 (medication* or drug* or prescription*)).ti,ab. 273374
19 ("use" adj3 (medication* or prescription*)).ti,ab. 84320
20 (deprescrib* or deprescription* or "de-prescrib*" or "de-prescription*" or "over-prescrib*" or overprescrib* or "potentially inappropriate medication*" or "potentially inappropriate drug*" or "potentially inappropriate prescription*" or "potentially inappropriate prescribing" or PIM or PIMS or polypharmacy or "multi-drug therapy" or "multidrug therapy" or polymedication or "medication regimen" or "drug regimen" or "prescription regimen").ti,ab. 42304
21 10 or 11 or 12 or 13 or 14 or 15 or 16 or 17 or 18 or 19 or 20 458295
22 4 and 9 and 21 5530
23 limit 22 to english language 5287
24 (conference abstract or conference review).pt. 4944543
25 23 not 24 3303

Ovid International Pharmaceutical Abstracts:

1 (frail* or sarcopeni* or sarcopaeni* or weak* or feeble or debilit*).ti,ab. 7835
2 (aged or "older adult*" or "older person*" or "older people*" or "older generation" or "older individual*" or "older patient*" or "older man" or "older men" or "older woman" or "older women" or "old age" or elder* or geriatric or "senior citizen*" or retiree* or septuagenarian* or octagenarian* or octogenarian* or nonagenarian* or centenarian* or "nursing home*").ti,ab. 34288
3 ((error* or erroneous or wrong or reconciliation) adj3 (medication or drug or prescription)).ti,ab. 4996
4 ((manag* or monitor* or discontinu* or taper* or stop* or reduc* or ceas* or decreas* or review* or titrat* or start* or initiat* or adjust* or switch* or change*) adj3 (medication* or drug* or prescription*)).ti,ab. 31454
5 ("use" adj3 (medication* or prescription*)).ti,ab. 7235
6 (deprescrib* or deprescription* or "de-prescrib*" or "de-prescription*" or "over-prescrib*" or overprescrib* or "potentially inappropriate medication*" or "potentially inappropriate drug*" or "potentially inappropriate prescription*" or "potentially inappropriate prescribing" or PIM or PIMS or polypharmacy or "multi-drug therapy" or "multidrug therapy" or polymedication or "medication regimen" or "drug regimen" or "prescription regimen").ti,ab. 3388
7 3 or 4 or 5 or 6 41452
8 1 and 2 and 7 172
9 limit 8 to english language 155

Scopus:

(TITLE-ABS((frail* OR sarcopeni* OR sarcopaeni* OR weak* OR feeble OR debilit*)) AND TITLE-ABS((aged OR "older adult*" OR "older person*" OR "older people*" OR "older generation" OR "older individual*" OR "older patient*" OR "older man" OR "older men" OR "older woman" OR "older women" OR "old age" OR elder* OR geriatric OR "senior citizen*" OR retiree* OR septuagenarian* OR octagenarian* OR octogenarian* OR nonagenarian* OR centenarian* OR "nursing home*")) AND TITLE-ABS((error* OR erroneous OR wrong OR reconciliation) W/3 (medication OR drug OR prescription)) OR TITLE-ABS((manag* OR monitor* OR discontinu* OR taper* OR stop* OR reduc* OR ceas* OR decreas* OR review* OR titrat* OR start* OR initiat* OR adjust* OR switch* OR change*) W/3 (medication* OR drug* OR prescription*)) OR TITLE-ABS(use W/3 (medication* OR prescription*)) OR TITLE-ABS((deprescrib* OR deprescription* OR "de-prescrib*" OR "de-prescription*" OR "over-prescrib*" OR overprescrib* OR "potentially inappropriate medication*" OR "potentially inappropriate drug*" OR "potentially inappropriate prescription*" OR "potentially inappropriate prescribing" OR pim OR pims OR polypharmacy OR "multi-drug therapy" OR "multidrug therapy" OR polymedication OR "medication regimen" OR "drug regimen" OR "prescription regimen")) AND LANGUAGE(english))
2,401 results

EBSCOhost CINAHL:

#	Query	Limiters/Expanders	Last Run Via	Results
S27	S4 AND S9 AND S25	Limiters - English Language<br>Expanders - Apply equivalent subjects<br>Search modes - Boolean/Phrase	Interface - EBSCOhost Research Databases<br>Search Screen - Advanced Search<br>Database - CINAHL	1,586
S26	S4 AND S9 AND S25	Expanders - Apply equivalent subjects<br>Search modes - Boolean/Phrase	Interface - EBSCOhost Research Databases<br>Search Screen - Advanced Search<br>Database - CINAHL	1,621
S25	S10 OR S11 OR S12 OR S13 OR S14 OR S15 OR S16 OR S17 OR S18 OR S19 OR S20 OR S21 OR S22 OR S23 OR S24	Expanders - Apply equivalent subjects	Interface - EBSCOhost	113,069

		Search modes - Boolean/Phrase	Research Databases Search Screen - Advanced Search Database - CINAHL	
S24	AB (deprescrib* or deprescription* or "de-prescrib*" or "de-prescription*" or "over-prescrib*" or overprescrib* or "potentially inappropriate medication*" or "potentially inappropriate drug*" or "potentially inappropriate prescription*" or "potentially inappropriate prescribing" or PIM or PIMS or polypharmacy or "multi-drug therapy" or "multidrug therapy" or polymedication or "medication regimen" or "drug regimen" or "prescription regimen")	Expanders - Apply equivalent subjects Search modes - Boolean/Phrase	Interface - EBSCOhost Research Databases Search Screen - Advanced Search Database - CINAHL	8,272
S23	TI (deprescrib* or deprescription* or "de-prescrib*" or "de-prescription*" or "over-prescrib*" or overprescrib* or "potentially inappropriate medication*" or "potentially inappropriate drug*" or "potentially inappropriate prescription*" or "potentially inappropriate prescribing" or PIM or PIMS or polypharmacy or "multi-drug therapy" or "multidrug therapy" or polymedication or "medication regimen" or "drug regimen" or "prescription regimen")	Expanders - Apply equivalent subjects Search modes - Boolean/Phrase	Interface - EBSCOhost Research Databases Search Screen - Advanced Search Database - CINAHL	3,636
S22	AB "use" N3 (medication* or prescription*)	Expanders - Apply equivalent subjects Search modes - Boolean/Phrase	Interface - EBSCOhost Research Databases Search Screen - Advanced Search	23,979

			Database - CINAHL	
S21	TI "use" N3 (medication* or prescription*)	Expanders - Apply equivalent subjects Search modes - Boolean/Phrase	Interface - EBSCOhost Research Databases Search Screen - Advanced Search Database - CINAHL	5,499
S20	AB (manag* or monitor* or discontinu* or taper* or stop* or reduc* or ceas* or decreas* or review* or titrat* or start* or initiat* or adjust* or switch* or change*) N3 (medication* or drug* or prescription*)	Expanders - Apply equivalent subjects Search modes - Boolean/Phrase	Interface - EBSCOhost Research Databases Search Screen - Advanced Search Database - CINAHL	52,218
S19	TI (manag* or monitor* or discontinu* or taper* or stop* or reduc* or ceas* or decreas* or review* or titrat* or start* or initiat* or adjust* or switch* or change*) N3 (medication* or drug* or prescription*)	Expanders - Apply equivalent subjects Search modes - Boolean/Phrase	Interface - EBSCOhost Research Databases Search Screen - Advanced Search Database - CINAHL	12,651
S18	AB (error* or erroneous or wrong or reconciliation) N3 (medication or drug or prescription)	Expanders - Apply equivalent subjects	Interface - EBSCOhost Research	6,012

		Search modes - Boolean/Phrase	Databases Search Screen - Advanced Search Database - CINAHL	
S17	TI (error* or erroneous or wrong or reconciliation) N3 (medication or drug or prescription)	Expanders - Apply equivalent subjects Search modes - Boolean/Phrase	Interface - EBSCOhost Research Databases Search Screen - Advanced Search Database - CINAHL	4,653
S16	(MH "Medication Reconciliation")	Expanders - Apply equivalent subjects Search modes - Boolean/Phrase	Interface - EBSCOhost Research Databases Search Screen - Advanced Search Database - CINAHL	2,125
S15	(MH "Medication Review")	Expanders - Apply equivalent subjects Search modes - Boolean/Phrase	Interface - EBSCOhost Research Databases Search Screen - Advanced Search Database - CINAHL	153

S14	(MH "Drug Monitoring")	Expanders - Apply equivalent subjects Search modes - Boolean/Phrase	Interface - EBSCOhost Research Databases Search Screen - Advanced Search Database - CINAHL	8,599
S13	(MH "Polypharmacy+")	Expanders - Apply equivalent subjects Search modes - Boolean/Phrase	Interface - EBSCOhost Research Databases Search Screen - Advanced Search Database - CINAHL	5,870
S12	(MH "Deprescribing")	Expanders - Apply equivalent subjects Search modes - Boolean/Phrase	Interface - EBSCOhost Research Databases Search Screen - Advanced Search Database - CINAHL	345
S11	(MH "Medication Management")	Expanders - Apply equivalent subjects Search modes - Boolean/Phrase	Interface - EBSCOhost Research Databases Search Screen - Advanced	1,746

			Search Database - CINAHL	
S10	(MH "Medication Errors+")	Expanders - Apply equivalent subjects Search modes - Boolean/Phrase	Interface - EBSCOhost Research Databases Search Screen - Advanced Search Database - CINAHL	18,283
S9	S5 OR S6 OR S7 OR S8	Expanders - Apply equivalent subjects Search modes - Boolean/Phrase	Interface - EBSCOhost Research Databases Search Screen - Advanced Search Database - CINAHL	1,197,156
S8	AB (aged or "older adult*" or "older person*" or "older people*" or "older generation" or "older individual*" or "older patient*" or "older man" or "older men" or "older woman" or "older women" or "old age" or elder* or geriatric or "senior citizen*" or retiree* or septuagenarian* or octagenarian* or octogenarian* or nonagenarian* or centenarian* or "nursing home*")	Expanders - Apply equivalent subjects Search modes - Boolean/Phrase	Interface - EBSCOhost Research Databases Search Screen - Advanced Search Database - CINAHL	396,735
S7	TI (aged or "older adult*" or "older person*" or "older people*" or "older generation" or "older individual*" or "older patient*" or "older man" or "older	Expanders - Apply equivalent subjects	Interface - EBSCOhost	195,879

	men" or "older woman" or "older women" or "old age" or elder* or geriatric or "senior citizen*" or retiree* or septuagenarian* or octagenarian* or octogenarian* or nonagenarian* or centenarian* or "nursing home*")	Search modes - Boolean/Phrase	Research Databases Search Screen - Advanced Search Database - CINAHL	
S6	(MH "Nursing Homes+")	Expanders - Apply equivalent subjects Search modes - Boolean/Phrase	Interface - EBSCOhost Research Databases Search Screen - Advanced Search Database - CINAHL	30,104
S5	(MH "Aged+")	Expanders - Apply equivalent subjects Search modes - Boolean/Phrase	Interface - EBSCOhost Research Databases Search Screen - Advanced Search Database - CINAHL	950,635
S4	S1 OR S2 OR S3	Expanders - Apply equivalent subjects Search modes - Boolean/Phrase	Interface - EBSCOhost Research Databases Search Screen - Advanced Search	104,808

			Database - CINAHL	
S3	AB (frail* or sarcopeni* or sarcopaeni* or weak* or feeble or debilit*)	Expanders - Apply equivalent subjects Search modes - Boolean/Phrase	Interface - EBSCOhost Research Databases Search Screen - Advanced Search Database - CINAHL	92,975
S2	TI (frail* or sarcopeni* or sarcopaeni* or weak* or feeble or debilit*)	Expanders - Apply equivalent subjects Search modes - Boolean/Phrase	Interface - EBSCOhost Research Databases Search Screen - Advanced Search Database - CINAHL	18,696
S1	(MH "Frail Elderly") OR (MH "Sarcopenia")	Expanders - Apply equivalent subjects Search modes - Boolean/Phrase	Interface - EBSCOhost Research Databases Search Screen - Advanced Search Database - CINAHL	12,930

Supplementary Table S3. Characteristics of the included studies (n= 223)

Study ID	Study design	Country	Participant characteristics				Duration of study	Follow-up
			Sample size (n=)	Study population	Mean Age (+/- standard deviation/IQR/ range)	Sex (%)		
1.#7863 (Ye., 2021)[161]	Cross-sectional study	UK, Greece, Croatia, Netherlands, Spain	2289	Community-dwelling older persons	79.7 (5.7)	M 910 (39.6)<br><br>F 1379 (60.4)	2015	No follow up
2. #176 (Thiruchelvam., 2021)[40]<br><br>2a. #116 (Thiruchelvam., 2021)[49]<br><br>2b. #134 (Thiruchelvam., 2021)[97]	Prospective cohort study	Australia	8996<br>No continuous polypharmacy in frail (n= 1263)<br>Continuous polypharmacy in frail (n= 820)<br>No continuous polypharmacy in non-frail (n= 5655)<br>Continuous polypharmacy in non-frail (n= 1258)	Older Women	No continuous polypharmacy in frail 79.68 (1.47)<br>Continuous polypharmacy in frail 79.65 (1.46)<br>No continuous polypharmacy in non-frail 79.50 (1.47)<br>Continuous polypharmacy in non-frail 79.48 (1.47)	Women only	Women aged 77–82 years in 2003, and 91–96 years in 2017 were analysed	15 years
2c. #243 (Thiruchelvam., 2021)[141]	Prospective cohort study	Australia	10334	Women aged 77–96 years		Women only	The first determined medication use from 2003 (age 77–82 years) to 2017 (age 91–96 years), and the second was cross-sectional at 2011 (age 85–90 years).	15 years
3. #713 (Jamsen., 2016)[63]	Prospective cohort study	Australia	1705	Community-dwelling men aged 70+ older	76 (8)*	Men only	January 2005-June 2007	2 year and 5 year follow up

4. #4168 (Gnjidic., 2012)[83]	Prospective cohort study	Australia	1662	Community dwelling older men	76.9 (5.4)	Men only	January 2005- June 2007	2 year follow up
5. #312 (Ambagtsheer., 2020)[102]	Cross-sectional study	Australia	228	Community dwelling adults aged 75 years or older	79 (6)*	M 103 (45.2)<br><br>F 125 (54.8)	March 2017- November 2018	No follow up
6. #2444 (Tembo., 2021)[139]	Cross-sectional study	Australia	581	Men aged 60-90 years of age	74 (67-83)*	Men only	2001 – 2006	No follow up
7. #1057 (Arakawa Martins., 2019)[163]	Secondary analysis of a cohort study	Australia	673	Healthy older adults from a community college for older adults	69.4 (4.5)	M 345 (43.2)<br><br>F 382 (56.8)	2014	No follow up
8. #2183 (Athuraliya., 2022)[184]	Prospective cohort study	Australia	4324	Community dwelling older men	Mean age 75.6 years) Proportion in each age group Age 69–74y= 2095 (49.5%) Age 75–79y= 1398 (33%) Age 80–84y= 627 (14.8%) Age 85+ y= 114 (2.7%)	Men only	Participants who completed the wave 2 questions In 2001- 2004	Followed-up at 12 and 24 months
9. #2292 (Thapaliya., 2021)[186]	Prospective cohort study	Australia	10372	Oldest birth cohort (1921–1926) of the Australian Longitudinal Study on women's Health (ALSWH)	Proportion in each age group Age ≤ 79= 5061 (48.8%) Age ≥ 80= 5311 (51.2%)	Women only	2003–2015	No follow up
10. #2746 (Tembo., 2020)[201]	Cross-sectional study	Australia	707: 360 women and 347 men	Aged ≥ 60 years	Women median 71.0 (IRQ: 65.2–77.5)*	Male 347 (49.1) Female 360 (50.9)	Assessed between 2016–2019 and 2011–2014, respectively	Data from the 15-year follow-up for both women (2011–2014)

								and men (2016–2019)
11. #3887 (Runganga., 2014)[207]	Prospective cohort study	Australia	351	Patients discharged home from hospital with support from Transition Care Program (TCP)	78.9 (8.8)	M 119 (34.3) F 228 (65.7)	November 2009-September 2010	No follow up
12. #576 (Thompson., 2018)[237]	Secondary analysis of a cohort study	Australia	909	Community-dwelling participants aged ≥65 years	65–74 years= 554 (56) >75 years= 355 (44)	M 435, (45) F 456, (55)	2004–2006	No follow up
13. #1091 (Gnjidic., 2012)[222]	Prospective cohort study	Australia	1705	Community-dwelling older men	76.9 (5.5)	Men only	January 2005-June 2007	2 years subsequently for follow-up assessment
14. #2663 (Lockery., 2020)[70]<br><br>14a. #1757 (Ekram., 2023)[174]<br><br>#14b. 376 (Ekram., 2022)[54]	Randomized-controlled trial	Australia, USA	19114 No polypharmacy= 14026<br><br>Polypharmacy= 4665<br><br>Hyperpolypharmacy= 423	Community-dwelling people aged 70 years or older	No polypharmacy median 73.8 (IQR 71.6-77.)* Polypharmacy median 74.6 (IQR 71.8- 78.5) Hyperpolypharmacy median 74.5 (IQR 71.8- 78.2)	M 8332, (44) F 10782, (56)	March 2010-December 2014	No follow up
15. #21 (Fravel., 2023)[93]	Secondary analysis of a trial	Australia, USA	15,732	Community-dwelling adults aged 70 years and older (65 and older for US minorities)	79.6 mean (range 79.5-79.7)	M 6861 (43.6) F 8871 (56.4)	2010 and 2014 Visits January 2017-January 2018	8- year and 11-year follow up
16. #3129 (Aprahamian., 2018)[25]	Cross-sectional analysis of cohort study	Brazil	629	Age ≥ 50 years, stable medical conditions over the past month	69.99 (7.04)	**M** 310 (49.3)<br><br>**F** 319 (50.7)	June 2014-July 2016	No follow up

17. #31<br>(Santos., 2023)[31]	Cross-sectional study	Brazil	216	Older adults 60 years old living in the urban area of the city	Proportion in each age group:<br>60- 79 y= 171 (79.2%)<br>≥ 80 y= 45 (20.8%)	M<br>101<br>(46.8)<br><br>F<br>115<br>(53.2)	2014	No follow up
18. #131<br>(Sousa., 2022)[38]	Cross-sectional study	Brazil	251 (65.4%) non-vulnerable and 133 (34.6%) vulnerable elders	Community dwelling adults >60 years old	Proportion in each age group:<br>Age 60- 79y= 113 (81.3%)<br>Age 80- 100 y= 26 (18.7%)	M<br>125<br>(32.6)<br><br>F 259<br>(67.4)	May 2018- June 2019	No follow up
19. #260<br>(Bolina., 2019)[45]	Cross-sectional study	Brazil	1607	Community-dwelling older adults	Proportion in each age group:<br>Age 60-70= 611 (38%)<br>Age 70-80= 707 (44%)<br>Age 80 or more= 289 (18%)	M<br>571<br>(35.5)<br>F 1036<br>(64.5)	2005, 2008, 2012	No follow up
20. #793<br>(Fernandes., 2021)[64]	Cross-sectional study	Brazil	265	Individuals >60 years old, residents of the urban area of Coari-AM	Proportion in each age group:<br>60- 69 years= 115 (43.3)<br>70- 79 years= 106 (40)<br>≥ 80 years= 44 (16.6)	M 99<br>(37.4)<br><br>F 166<br>(62.6)	October 2013-February 2015	No follow up
21. #2634<br>(Carneiro., 2020)[69]	Cross-sectional study	Brazil	394	Community-dwelling older people aged ≥ 60 years	73.9 (7.9)	M<br>131<br>(33.2)<br><br>F 263<br>(66.8)	November 2016-February 2017	No follow up
22. #3293<br>(Fhon., 2018)[74]	Longitudinal study	Brazil	262	Older adult aged ≥ 65 years living at home	79.3 (6.3)	M 88<br>(33.6)<br><br>F 174<br>(66.4)	Period 1: October 2007 - February 2008<br>Period 2,: July - December 2013	5 years

23. #3492 (Closs., 2016)[77]	Cross-sectional study	Brazil	521	Individuals aged 60 years or older, registered in the Family Health Strategy (FHS) program	68.5 (6.8)	M 186 (35.7)<br><br>F 335 (64.3)	March 2011-December 2012	No follow up
24. #4169 (Miguel., 2012)[84]	Cross-sectional study	Brazil	58	Community dwelling elderly with osteoarthritis	Non-frail 74 (5)<br>Pre-frail 73 (6)<br>Frail 75 (6)	M 4 (6.9)<br><br>F 54 (93.1)	first (2006-2009), second (2011- 2013), and third (2014-2017) waves of the study	No follow up
25. #141 (Oliveira., 2022)[86]	Cross-sectional study	Brazil	291	Older adults aged 65 years and older, residing in the community	Proportion in each age group:<br>65 to 69 y= 132 (45.4%)<br>70 to 79 y= 139 (47.8%)<br>80 years and older y= 20 (6.9%)	M 121 (41.6)<br><br>F 170 (58.4)	June-August 2018	No follow up
26. #1694 (Vendola., 2023)[109]	Cross-sectional study	Brazil	52	Non frail and frail older adults	Frail 84.8<br>Non frail 81.2	M 9 (17.3)<br><br>F 43 (82.7)		No follow up
27. #2063 (Ribeiro., 2022)[115]	Cross-sectional study	Brazil	106	Outpatients from a tertiary hospital with heart failure, aged ≥ 60	Median 68 (range 63-74)*	M 71 (67)<br><br>F 35 (33)		No follow up
28. #363 (Novaes., 2017)[143]	Cross-sectional study	Brazil	368	Older adults 60 years or older	73.80 (8.0)	M 142 (35.5)<br><br>F 258 (64.5)	October 2014-March 2015	No follow up

29. #2679 (MeloFilho., 2020)[196]	Cross-sectional study	Brazil	1,716	Community-dwelling older adults aged ≥60 years	71.0 (7.3)	M:<br>417<br>(24.3)<br><br>F<br>1299<br>(75.7)	March-September 2016	No follow up
30. #2929 (Carneiro., 2019)[210]	Longitudinal study	Brazil	394	Older adult residents in the urban region	≥60	M<br>130<br>(33)<br><br>F<br>264<br>(67)	Between May and July 2013-November 2016 and February 2017	November 2016 to February 2017
31. #2667 (Alves., 2020)[71]	Cross-sectional study	Brazil	580	Older adults aged 60 or older living in the urban area of the municipality	Proportion in each age group:<br>60- 69 y= 256 (44.1%)<br>70- 79y= 230 (39.7%)<br>80 or older= 94 (16.2%)	M<br>185<br>(31.9)<br><br>F<br>395<br>(68.1)	March-June 2016	No follow up
32. #930 (Larsen., 2020)[57]	Prospective cohort study	Canada	250428	Home care clients aged 65 years or older	Proportion in each age group:<br>65 -74 = 52868 (21.1)<br>75 -84 y= 107030 (42.7)<br>85 -94 y= 84049 (33.6)<br>>95 y= 6481 (2.6)	M<br>101009<br>(40.3)<br><br>F<br>149419<br>(59.7)	January 1, 2010-December 31, 2014	No follow up
33. #305 (Khera., 2019)[90]	Quasi-experimental pretest-posttest design	Canada	54	Community dwelling older adults 65 years and older with frailty who have polypharmacy and/or 2 or more chronic conditions	81.7 (6.74)<br>(range: 65-95)	M 21<br>(38.9)<br><br>F 33<br>(61.1)	April 2017-May 2018	No follow up
34. #1689 (Fikree., 2023)[108]	Retrospective pre-post study	Canada	658	Individuals ≥65 years of age with	Proportion in each age group:	M<br>257	February 19 2019-March 14	14 months

				a frailty assessment and 1 or more of the conditions	65-69y= 183 (27.8 %)<br>70-74 y= 185 (28.1%)<br>75-79 y= 127 (19.3%)<br>80-84 y= 87 (13.2%)<br>85 and older y= 76 (11.6%)	(39.1)<br><br>F<br>401<br>(60.9)	2020 and March 15 2020-May 9 2021	
35. #2855 (Wong., 2020)[216]	Cross-sectional study	Canada	33,663<br><br>Frail population<br>EMR: British Columbia= 91 (2.3)<br>Admin: British Columbia= 517 (12.9)<br>EMR: Manitoba= 1589 (39.5)<br>Admin: Manitoba= 1821 (45.3)	Aged 65 years and older who were frail	Proportion in each age group (frail):<br><br>British Columbia<br>65-74: 154 (22.7%)<br>75-84: 240 (35.4%)<br>85+: 284 (41.9%)<br>Manitoba<br>65-74: 831 (22.4%)<br>75-84: 1314 (35.3%)<br>85+: 1572 (42.3%)	EMR: British Columbia<br>M 36 (39.6)<br>F 55 (60.4)<br>Admin: British Columbia<br>M 217 (42)<br>F 300 (58)<br>EMR: Manitoba<br>M 583 (36.7)<br>F 1006 (63.3)<br>Admin: Manitoba<br>M 658 (36.1)<br>F 1163 (63.9)	Jan. 1, 2013-Dec. 31, 2014	Not mentioned
36. #2775 (Arauna., 2020)[123]	Cross-sectional study	Chile	1,205	Older adults aged 65 and older from four urban provincial capitals and two rural communes per capital	73.1 (5.9)	M<br>385<br>(32)<br><br>F<br>819<br>(68)	September 2016 -October 2017	No follow up
37. #3705 (Wang., 2015)[82]	Prospective cohort study	China	1562	Older men aged ≥80 years at a geriatric outpatient clinic	85.2 (range 80-104)	Men only	2009	5-year follow up

38. #95 (Xu., 2022)[95]	Cross-sectional study	China	231	Outpatients ≥ 60 years in the West China Hospital	Proportion in each age group: 60-74y= 152 (65.8%) 75-89 y= 70 (30.3%) ≥ 90 y= 9 (3.9%)	M 87 (37.7) F 144 (62.3)	December 8, 2020-April 30, 2021	No follow up
39. #1885 (Liu., 2023)[178]	Prospective cohort study	China	422	Patients (≥ 40 years old) with type 2 diabetes	70.35 (10.71)	M 270 (64) F 152 (36)	Data for the first participant were collected in January 2018	Follow-up was conducted after 4.5 years
40. #2858 (Meng., 2019)[218]	Cross-sectional study	China	101	Chinese men aged 60 years and over	79.4 (7.7) (range 63–95 years)	Men only	October 2015-October 2016	No follow up
41. #3925 (Woo., 2014)[232]	Cross-sectional study	China	4000	Community-living cohort of 4,000 men and women aged 65 years and over			2001-2003	4 years and returned for assessment during the fifth year
42. #772 (Woo., 2015)[234]	Cross-sectional study	China	816	Older Chinese people aged 65 years and older	Pre-frail 76.3 (7.4) Frail 80.0 (8.0)	M 119 (15) F 697 (85)		Not mentioned
43. #705 (Wang., 2015)[236]	Prospective cohort study	China	Beijing urban Male 2432 Beijing rural Male 419 Hong Kong male 2000 Beijing urban Female 3888 Beijing rural Female 559 Hong Kong Female 2000	Older Chinese people aged 65 years and older	Beijing urban Male 74.62 (5.62) Beijing rural Male 74.89 (5.79) Hong Kong male 74.47 (5.50) Beijing urban Female 73.85 (5.28)	Beijing urban M 2432 (38.5) F 3888 (61.5) Beijing rural M 419 (42.8) F 559 (57.2) Hong Kong	July-November 2009	No follow up

					Beijing rural Female 73.94 (5.07)<br>Hong Kong Female 73.73 (5.32)	M 2000 (50)<br>F 2000 (50)		
44. #2664 (Qiao., 2020)[248]	Cross-sectional study	China	780	Community-dwelling older patients	69.9 (6.5)	M 238 (30.5)<br><br>F 542 (69.5)	July-December 2016	No follow up
45. #5782 (GüngörBaşaran., 2021)[229]	Cross-sectional study	Cyprus	347	Individuals aged 65 and older residing in the Famagusta district of Cyprus	73.12 (6.78)	M 95 (59.01)<br><br>F 123 (66.13)	July 2018-September 2019	No follow up
46. #1709 (Anjum., 2023)[153]	Retrospective cohort study	Denmark	42,320	Heart Failure Patients aged 18-95	Median 74.3 (range 57.4–91.2)*	M 26,208 (61.9)<br>F 16,112 (38.1)	2014-2021	No follow up
47. #73 (Ye., 2022)[26]<br><br>47a. #24 (Ye., 2022)[48]	Longitudinal study	Netherlands, Greece, Croatia, Spain, United Kingdom	1791	Community-dwelling older -adults	79.6 (5.6)	M 702 (39.2)<br><br>F 1089 (60.8)	May 2015-June 2017	12 months follow up
48. #2490 (Midão., 2021)[59]	Longitudinal cohort study	Europe (Austria, Belgium, Croatia, Czech Respublic, Denmark, Estonia,	24693	Community-dwelling older adults	74.5 (6.9)	M 10983 (44.5)<br><br>F 13710 (55.5)	Wave 6 (with data from 2015) and wave 7 (with data from 2017), are the most recent waves. In this work, we used	Median follow-up time was 25 months (23–26, IQR)

		France, Germany, Greece, Israel, Italy, Luxembourg, Poland, Portugal, Slovenia, Spain, Sweden, Switzerland)					data from wave 6 and 7	
49. #254 (Jazbar., 2021)[60]	Retrospective cohort study	Europe (Austria, Germany, Sweden, Spain, Italy, France, Denmark, Switzerland, Belgium, Israel, Czech Republic, Luxembourg, Slovenia and Estonia)	22798<br><br>Non-frail population at baseline based on frailty index (FI-population)= 14665<br><br>Non-frail population at baseline based on frailty phenotype (FP-population)= 8133	Older adults	FI 72.5 (6)<br><br>FP 71.6 (5.4)	M 11556 (51)<br><br>F 11242 (49)	The first wave began in 2004 (Wave 1), and new waves are released approximately every two years; the most recent data from regular questionnaires were used in the current study (Waves 5 and 6)	2 year follow up
50. #258 (Cheung., 2020)[101]	Longitudinal study	Hong Kong, Israel, European countries	44,818	Community-dwelling older adults aged 60 or above	69.61 (9.81)	M 18,517 (41.3)<br><br>F 26,301 (58.7)	2015-2018	1-2 year follow-up
51. #2161 (DeGodoiRezendeCostaMolino., 2022)[117]	Secondary analysis of a trial	(European countries: Basel, Berlin, Coimbra, Geneva, Innsbruck, Toulouse and Zurich	2157	Community-dwelling adults age 70 and older	Median 74 (range 72-77)*	M 826 (38.3)<br>F 1331 (61.7)	December 2012 -November 2014	3 year follow-ups

52. #8386 (Strandberg.,2018)[159]	Secondary analysis of a cohort study	Finland	530<br>All current nonusers of statin= 301<br>Continuous Nonusers of Statin= 230<br>Discontinued After 2010= 71<br>Current Statin User in 2015= 229	Cohort of men born 1919–1934 (original n = 3,490)	All current nonusers of statin= median 86 (IQR 84-89)*<br>Continuous Nonusers of Statin= median 86 (IQR84–89)*<br>Discontinued After 2010= median 87 (IQR 84–90)*<br>Current Statin User in 2015= median 85 (IQR 84–88)*	Men only	Cohort of men born 1919–1934	Followed-up since the 1960s
53. #1382 (Lampela., 2016)[168]	Retrospective cohort study	Finland	621	Community-dwelling persons aged 75 and older were randomly selected from population			Data is from 2004-2007	No follow-up
54. #4023 (Koponen., 2013)[208]	Cross-sectional study	Finland	605	Community-dwelling people aged 75 years old	Proportion in each age group:<br>76–79 y= 258 (42.6%)<br>80–84 y= 207 (34.2%)<br>≥85 y= 140 (23.1%)	M<br>181<br>(29.9)<br><br>F<br>424<br>(70.1)		No follow-up
55. #1287 (Rieckert., 2018)[106]	Secondary analysis of a trial	UK/Manchester, Italy/Bolzano, Austria/Salzburg, Germany /Rostock, Germany	3,904	Adults age ≥ 75 taking ≥8 medications regularly	81.5 (4.4)	M<br>1664<br>(42.6)<br><br>F<br>2240<br>(57.4)	September 2014 -September 2015	No follow-up
56. #69 (Tchalla., 2022)[29]	Cross-sectional study	France	753	Community-dwelling adults	83.1 (5.8)	M<br>244	January 2010 - 31 August 2017	No follow - up

				over 75 years, or over 65 years with at least two comorbidities		(32.4)<br><br>F<br>509<br>(67.6)		
57. #196 (Rhalimi., 2021)[41]	Cross-sectional study	France	4090	Patients over 65 years of age who attended the community pharmacy to receive at least one prescription drug	77.5 (7.6)	M<br>1631<br>(39.9)<br><br>F<br>2459<br>(60.1)	March 20-May 30 2017	No follow - up
58. #952 (Martinot., 2018)[66]	Prospective cohort study	France	Population by year, 2012= 11891<br><br>2013= 12405<br><br>2013= 11732	Community-dwelling people aged 58 to 73 in 2012, and followed for 3 years	Proportion in each age group: 2012, < 65 years= 3847 (32.4%); 65-70 y= 5492 (46.2%); ≥ 70 y= 2552 (21.5%) 2013, < 65 years=2488 (20.1%); 65- 70 y= 6259 (50.5%); ≥ 70 y= 3658 (29.5%)<br><br>Proportion in each age group: 2014, < 65 years= 1165 (9.9%); 65-70 y= 6125 (52.2%); ≥ 70 y= 4442 (37.9%)	**2012**<br>M<br>8802<br>(74)<br><br>F<br>3089<br>(26)<br><br>**2013**<br>M<br>9172<br>(73.9)<br><br>F<br>3233<br>(26.1)<br><br>**2014**<br>M<br>8681<br>(74)<br><br>F<br>3051<br>(26	2012, 2013, 2014	3 Year follow up
59. #341 (Reallon., 2020)[105]	Cross-sectional study	France	403	Community-dwelling patients	80.48 (6.38)	M<br>175	July 2017-March 2018	No follow-up

				aged 65 years and over		(43.4)<br><br>F<br>228<br>(56.6)		
60. #2154 (Pilleron., 2022)[116]	Secondary analysis of a cohort study	France	643	Noninstitutionalized adults aged 75 years or older living in Bordeaux suburbs	82.1 (4.3)	M<br>230<br>(35.8)<br><br>F<br>413<br>(64.2)	2009-2010	4 years of follow-ups
61. #1188 (Herr.,2019)[166]	Randomized-controlled trial	France	842	Adults aged 70 and over who did not receive the multidomain intervention	Proportion in each age group:<br>70-74 y= 396 (49.1%)<br>75-79 y= 262 (32.5%)<br>80 y and over= 149 (18.5%)	M<br>285<br>(33.3)<br><br>F<br>522<br>(64.7)	May 2008-February 2011	Follow-up visits were scheduled every 6 months up to 36 months
62. #1717 (Tabue-Teguo., 2023)[173]	Cross-sectional study	France	115	Community-dwelling older adults (>65 years old)	76.0 (7.8)	M<br>37 (32.2)<br><br>F<br>78 (67.8)	October 1, 2019-March 15, 2020	No follow-ups
63. #3659 (Moulis., 2015)[203]	Cross-sectional study	France	437	Patients consulting for the first time at the Geriatric Frailty Clinic	83.0 (6.1)	M<br>163<br>(37.3)<br><br>F<br>274<br>(62.7)	January 2013-October 2013	No follow-up
64. #3751 (Herr., 2015)[217]	Cross-sectional study	France	2,350	People aged 70 years and over	Proportion in each age group:<br>70–79 y= 863 (36.7%)<br>80–89 y= 975 (41.5%)	M<br>955<br>(40.6)<br><br>F<br>1395	2008–2010	No follow up

					90 years and over= 512 (21.8%)	(59.4)		
65. #386 (Herr., 2017)[219]	Cross-sectional study	France	1,890	Community-dwellers aged 65 and over	74.7 (7.4)	M 887 (39.5)<br><br>F 1003 (60.5)	2012	No follow up
66. #804 (Moulis., 2015)[233]	Cross-sectional study	France	437	Patients consulting for the first time at Geriatric Frailty Clinic	83.05 (6.15)	M 163 (37.3)<br><br>F 274 (62.7)	January 2013-October 2013	No follow up
67. #3470 (Tabue-Teguo., 2018)[239]	Cross-sectional study	France	630	Adults aged 75 years or older participating in the 10-year follow-up of the Bordeaux suburbs study	83.3		2009	10-year follow-up
68. #496 (Rhalimi., 2018)[130]	Cross-sectional study	France	892	Adults aged over 65 years who are regular pharmacy customers taking at least one drug	80.6 (6.6) (range 65-102)	M 340 (38)<br><br>F 552 (62)	January-May 2014	No follow up
69. #110 (Mortsiefer., 2023)[35]	Cluster randomized trial	Germany	521 Intervention (COFRAIL intervention consisted of 2 steps. The 2 steps consisted of 3 education sessions and 3 family conferences)= 272 Control (received usual care)= 249	Community-dwelling adults aged 70 years or older with frailty syndrome, daily use of at least 5 different medications, a	Intervention 83.6 (6.08)<br><br>Control 83.2 (6.29)	Intervention M 91 (33.5) F 181 (66.5)<br><br>Control M 74 (29.7) F 175 (70.3)	April 30, 2019 - June 30, 2021	12 months T0 (baseline), T1 (6-month follow-up), and T2 (12-month follow-up)

				life expectancy of at least 6 months, and no moderate or severe dementia				
70. #536 (Saum., 2017)[47]	Secondary analysis of a cohort study	Germany	3058	Community-dwelling adults aged between 57 and 84 years	69.6 (6.3)	M 1456 (47.6)<br><br>F 1602 (52.4)	July 2000 - December 2002	8-year and 11-year follow-up, conducted between July 2008 and December 2010 and between July 2011 and December 2013
71. #264 (Muhlack., 2019)[51]<br><br>71a. #273 (Muhlack., 2018)[52]	Prospective cohort study	Germany	2865	Participants age between 50 and 75 years proficient in German	70.2 (5.9)	M 1385 (48.3)<br><br>F 1480 (51.7)	2000 - 2002	8 year follow up home visit used as the baseline; 11 year follow up; 14 year follow up
72. #2938 (Braun., 2019)[72]	Cross-sectional study	Germany	258	Community-dwelling older physiotherapy patients	73.8 (5.6)	M 97 (38)<br><br>F 161 (62)	Phase 1: - October 2015; Phase II:- October 2016 - April 2017	No follow up
73. #3457 (König., 2018)[76]	Secondary analysis of a cohort study	Germany	1502	Community-dwelling, comparably well-functioning and aged between 60 and 84 years	68.7 (3.7)	M 741 (49.3)<br><br>F 761 (50.7)	2009 - 2013	No follow up

74. #1446 (Buttery.,2015)[152]	Secondary analysis of a cohort study	Germany	1843	Community-dwelling people aged 65–79 years	Proportion in each age group: 65- 69 y= 641 (34.8%) 70- 74 y= 792 (43%) 75- 79 y= 407 (22.1%)	M 919 (49.8) F 924 (50.2)	2008–2011	No follow up
75. #2155 (Toepfer., 2022)[183]	Secondary analysis of a cohort study	Germany	1382	Adults who were at least 65 years old at baseline assessments	69 (67-71)*	M 673 (48.7) F 709 (51.3)		No follow-up
76. #3742 (Meid., 2015)[205]	Prospective cohort study	Germany	1454	Men and women, aged 50–74 years at baseline	71.1 (6.1)	M 672 (46.2) F 782 (53.8)	July 2008 - December 2010	Follow-ups after 2, 5, 8, and 11 years
77. #3 (Tampaki., 2023)[91]	Prospective cohort study	Greece	104	Outpatients ≥ 65 years in a rural Greek primary care center	Median 78 (IQR 71.5 - 83.5)*	M 53 (51) F 51 (49.1)	February - October 2019	6-month foll ow up
78. #9308 (Panagiotakis., 2022)[156]	Prospective cohort study	Greece	186	A rural cohort of 186 subjects aged 60-89 years recruited from a community-based study	Frail 81.8 (6.1) Pre-frail 77.2 (7.5) Robust 72.9 (6.6)	Frail M 3 (18.8) F 13 (81.2) Pre-frail M 26 (36.6) F 45 (63.4)	March 2013 - June 2015	No follow up

						Robust M 33 (33.3) F 66 (66.7)		
79. #1839 (Kravvariti., 2023)[177]	Cross-sectional study	Greece	53<br>Non frail 21<br>Frail 32	Community-dwelling older adults, aged 65–102 years	Non-frail 76 (9)<br>Frail 86 (8)	M<br>19 (36)<br><br>F<br>34 (64)		No follow up
80. #3097 (Panagiotakis., 2018)[213]	Cross-sectional study	Greece	402<br>CNI cognitively non-impaired 127<br>MCI mild cognitive impairment 175<br>PwD persons with dementia 100	Persons aged 60–100 years	CNI 73.5 (6.9)<br>MCI 76.9 (6.7)<br>PwD 79.7 (5.9)	M<br>129<br>(32)<br><br>F<br>273<br>(68)		No follow up
81. #9594 (Panda., 2020)[155]	Cross-sectional study	India	127	Elderly (>60 years) residing in a peri-urban slum area in Delhi	Proportion in each age group:<br>age 60-65= 66 (52%)<br>age 65-75= 42 (33%)<br>age 75 above= 19 (15%)	M<br>54<br>(42.5)<br><br>F<br>73<br>(57.5)	December 2018 - July 2019	No follow-up
82. #2454 (Setiati., 2021)[191]	Secondary analysis of a cohort study	Indonesia	908	Community-dwelling outpatients aged 60 years and older without acute illness in nine geriatric service care centres	Proportion in each age group:<br>60–69 years old= 484 (53.3%)<br>> 70 years old= 424 (46.7%)	M<br>438<br>(48.2)<br><br>F<br>470<br>(51.8)	March - October 2020	5 years follow up
83. #96 (Sobhani., 2022)[34]	Cross-sectional study	Iran	1529	Community-dwelling older adults ≥60 years living in urban and rural regions of Birjand county	70.6 (8.2) (range 60- 97)	M<br>740<br>(48.4)<br><br>F<br>789<br>(51.6)	2018	No follow up

84. #1716 (Badrkhahan., 2023)[110]	Cross-sectional study	Iran	2,392	Urban residents from Bushehr in the south of Iran.	70.13 (range 70.07-70.19)	M 1163 (48.63) F 1229 (51.37)	first stage were implemented from March 2013 to October 2014. The second stage has begun from October 2015, and data recollection is designed to be in 2.5-year intervals	Every 5 years for three consecutive periods (a total of 15 years of follow-up
85. #5848 (Saeidimehr., 2021)[228]	Cross-sectional study	Iran	540	Older adults aged ≥60 years in Khuzestan province	72.61 (8.72)	M 186 (34.4) F 354 (65.6)	2019	No follow up
86. #48 (Zuniga., 2023)[30]	Cross-sectional study	Ireland	523	Community dwelling adults	68.8 (7.3)	M 254 (48.6) F 269 (51.4)	March 2014–December 2015	No follow up
87. #2368 (O'Donoghue., 2021)[119]	Pilot study	Ireland	104	Patients aged 50 years or more who attended FASU in St. James's Hospital	Non-frail median 72 (IQR 68.3-76.8)* Pre-frail median 76 (IQR 68.8-80.8)* Frail median 74.5 (IQR 68-80.5)*	M 36 (35) F 68 (65)	November 23, 2019 - March 9, 2020	No follow up
88. #2808 (O'Connell., 2020)[124]	Cross-sectional study	Ireland	570	Older adults with intellectual disability (ID)	Proportion in each age group: 44- 59 y= 381 (66.8%) 60+ y= 189 (33.2%)	M 237 (42) F 333 (58)	Data drawn from 2013/2014 of the Intellectual Disability Supplement to the Irish Longitudinal Study on Ageing	No follow up

89. #2439<br>(O'Donovan., 2021)[190]	Secondary analysis of a cohort study	Ireland	97,691	Adults aged ≥50 years	64.40 (9.89)	M<br>44,612<br>(45.7)<br><br>F<br>53,079<br>(54.3)	Wave 1 (2004/05), 1956 or earlier in Wave 2 (2005/06) and 1960 or earlier in Wave 4 (2010/11)	
90. #9238<br>(Byrne., 2019)[227]	Retrospective cohort study	Ireland	Cohort 1: 1924 Cohort 2: 1781	Individuals aged ≥65 years participating in TILDA and enrolled in the General Medical Services scheme	Cohort 1: 75.0 (6.1)<br>Cohort 2: 75.3 (6.1)	**Cohort 1**<br>M<br>872<br>(45.3)<br><br>F<br>1052<br>(54.7)<br><br>**Cohort 2**<br>M<br>804<br>(45.1)<br>F<br>977<br>(54.9)	October 2009 - February 2011	No follow up
91. #415<br>(Sargent., 2020)[55]	Cross-sectional study	Italy	1479<br>Frailty (cardiovascular health study (CHS)) 595<br>Cognitive frailty (MMSE) (Frail (≥ 1 criterion) and cognitive impairment (MMSE ≤ 23)) 257<br>Cogntive frailty (Frail (≥1 criterion) and cognitive impairment (Trail A ≥ 78) 302<br>Frail (≥1 criterion) and cognitive impairment (Trail B ≥ 106) 325	Adults aged 20–102 years	78 (7.9)<br><br>82 (7.4)<br><br>78 (7.4)<br><br>76 (6.9)	M 519 (35.1)<br>F 960 (64.9)	1998 - 2000	No follow up
92. #247<br>(Salaffi., 2021)[100]	Cross-sectional study	Italy	350<br>Symptomatic knee osteoarthritis (KOA) 170	Age ≥ 50 years, morning stiffness < 30 min	KOA 70.1 (7.1) (range 53-81)	M<br>79<br>(22)	2018	No follow-up

			Healthy controls 186	evaluated by the WOMAC stiffness subscale, other characteristics of enlarged/tender knees	Healthy 69.1 (8.2) (range 54-83)	F 277 (78)		
93. #1842 (Rea., 2023)[137]	Case-control study	Italy	49,201 Patients who died (cases)= 26,035 Patients who survived (controls)= 26,035	Older adults ≥65 years who received ≥3 consecutive prescriptions of antidiabetic agents during 2012	Patients who died (cases) 79.7 (7.2) Patients who survived (controls) 79.6 (7.2)	M 26035 (52.9) F 23166 (47.1)	Throughout 2012	Follow-up periods for the cohort members from 2012 to 2018
94. #633 (Trevisan., 2017)[147]	Longitudinal study	Italy	2,925	Individuals aged 65 and older	74.4 (7.3)	M 1179 (40) F 1746 (60)	1995–97	mean follow-up of 4.4 years
95. #122 (Kume., 2021)[36]	Cross-sectional study	Japan	313	Older adults aged ≥65 years	Robust social frail 74.6 (5.7) Prefrailty social 75.7 (5.9) Frailty social 74.8 (5.9)	M 110 (35) F 213 (68)	2018 - 2020	No follow-up
96. #250 (Bonfiglio., 2019)[44]	Cross-sectional study	Japan	160	Individuals with a Clinical Dementia Rating of 0.5–1 and MMSE score of 20–30	78.3 (5.8)	M 73 (45.6) F 87 (54.4)		No follow-up
97. #2474 (Uragami., 2021)[58]	Cross-sectional study	Japan	923 Nonfrail (frailty score 0)= 556 Pre-frail (frailty score 1-2)= 318	Outpatients aged 65–74 years who visited any of 11 community pharmacies	Nonfrail median 70 (IQR 68- 72)* Pre-frail median 71 (IQR 68.25-72)	M 423 (47) F	February - April 2020	No follow-up

			Frail (frailty score ≥ 3)= 49		Frail median 70 (IQR 68- 72)	491 (53)		
98. #2531 (Kume., 2021)[68]	Cross-sectional study	Japan	313	Community-dwellers aged 65 or more	Robust 73.7 (5.4)<br>Prefrail 74.7 (5.9)<br>Frail 77.3 (6.2)	M<br>98<br>(31)<br><br>F<br>215<br>(69)	2018 - 2020	No follow up
99. #1925 (Kinoshita., 2022)[113]	Cross-sectional study	Japan	555	Outpatients of a frailty clinic aged ≥ 65 with no history of erythropoietin administration	77.7 (6) (range 65- 96)	M<br>181<br>(33)<br><br>F<br>374<br>(67)	June 2017 - May 2020	No follow-up
100. #413 (Yuki., 2018)[129]	Longitudinal Study	Japan	299	Older adults	70.6 (4.2) (range 65-81)	M<br>166<br>(55.5)<br><br>F<br>133<br>(44.5)	the first- wave examination (November 1997 to April 2000). Second-wave exami-nations of the NILS-LSA between April 2000 and May 2002. NILS-LSA fifth-wave examination; from July 2006 to July 2008	6-year follow-up
101. #1609 (Mizuno., 2023)[171]	Cross-sectional study	Japan	866	Community-dwelling older adults in their 70s and 80s	Proportion in each age group:<br>70s= 561 (64.8%)<br>80s= 305 (35.2%)	M<br>426<br>(49.2)<br><br>F<br>440<br>(50.8)	2017 - 2018	follow-ups every 3years (2017)

102. #1808 (Nishimura., 2023)[176]	Retrospective cohort study	Japan	12,585	Patients aged ≥65 years with nonvalvular AF	Median 80.0 (IQR 72.0- 85.0)*	M 6872 (54.6)<br><br>F 5713 (45.4)	2012 - 2018	Follow-up of outcomes started in the index month (first OAC prescription)
103. #2656 (Kabayama., 2020)[193]	Cross-sectional study	Japan	2,235	Community-dwelling older Japanese aged 70 ± 1 (n = 1000), 80 ± 1 (n = 978), and 90 ± 1 (n = 272) years	a Proportion in each age group: ge 70 ± 1= 1000 (44.7%)<br>age 80 ± 1= 978 (43.8%)<br>age 90 ± 1= 272 (2.2%)	M 1048 (47)<br><br>F 1187 (53)	Completed the baseline survey in 2010, 2011, and 2012	No follow-up
104. #2881 (Chen., 2019)[202]	Prospective cohort study	Japan	1,554	Older adults aged 65 years and over who were initially free of long-term care needs at baseline	CHS: Robust= 71.5 (5.1)<br>CHS: Pre-frail= 74.0 (5.9)<br>CHS: Frail= 79.3 (6.2)<br>FRAIL: Robust= 71.8 (5.2)<br>FRAIL: Pre-frail= 74.6 (6.3)<br>FRAIL: frail= 76.3 (6.7)	M 616 (40)<br><br>F 938 (60)	May - August 2011	Follow-up time was counted from the date of baseline survey until the date of being ascertained as needing long-term care, death, loss-to-follow-up, or March 31, 2017
105. #3274 (Takeuchi., 2018)[215]	Cross-sectional study	Japan	388	Japanese Hemodialysis Patients	Not-frail 63.6 (11.2)<br>Pre-frail 67.5 (12.3)<br>Frail 71.0 (10.2)	M 242 (62.3)<br>F 146 (37.7)	October 2015 - January 2016	No follow up
106. #8051 (Hironaka., 2020)[240]	Cross-sectional study	Japan	682	Community-dwelling individuals aged ≥ 65 years	73.3 (6.6)	M 267 (39.1)<br>F 415 (60.9)	2013–2018	No follow up

107. #9428 (Okui., 2023)[249]	Retrospective cohort study	Japan	318	Frail patients aged ≥ 65 years with GSM who underwent pelvic floor muscle training	Ninjin'yoeito (NYT) group= 78.13 (5.93) No-NYT group= 78.03 (5.76)	Women only	November 2016 - November 2022	Median follow-up of 14.5 months
108. #221 (Kim., 2021)[61]	Cross-sectional study	Korea	818337	South Korean older adults aged 66 from 2009 to 2012		M 361056 (44.1)<br><br>F 457281 (55.9)	2009 - 2012	No follow-up
109. #3642 (Jung., 2016)[80]	Secondary analysis of a cohort study	Korea	382	Adults aged ≥ 65 years residing in rural communities of Korea	74.4 (6.5)	M 167 (43.8)<br><br>F 215 (56.2)	October 2014 - December 2014	No follow-up
110. #784 (Kim., 2021)[88]	Secondary analysis of a cohort study	Korea	1374	Community-dwelling older adults	75.90 (3.85)	M 665 (48.4)<br><br>F 709 (51.6)	July 2018 - March 2019	No follow up
111. #390 (Mi-Ji Kim., 2019)[144]	Cross-sectional study	Korea	808	Community-dwelling men and women aged 65 years and older in 2 rural towns	74.58 (6.36)	M 331 (41)<br>F 477 (59)	June - August 2018	No follow-up
112. #928 (Lee.,2020)[149]	Cross-sectional study	Korea	1264	Women (70–84 years) from the Korean and Aging Cohort Study	Frail 78.2 (3.7) Non-frail 75.3 (3.8)	Women only	2009 - 2012	No follow up
113. #2718 (Shim., 2020)[198]	Cross-sectional study	Korea	2,128	Community-dwelling adults	Men non-frail 76.3 (3.8)	M 986	2016 - 2017	No follow up

				aged between 70 and 84 years	Men frail 79.39 (3.1)<br>Women non-frail 75.4 (3.7)<br>Women frail 78.2 (3.7)	(46.3)<br><br>F<br>1142<br>(53.7)		
114. #3545 (Jung., 2016)[252]	Cross-sectional study	Korea	103	Patients aged ≥ 65 years	Robust 75.6 (5.9)<br>Pre-frail 76.6 (6.0)<br>Frail 79.1 (6.4)	M<br>48<br>(47)<br><br>F<br>55<br>(53)	July 2014 - August 2014	No follow up
115. #2526 (Jung., 2021)[253]	Cross-sectional study	Korea	2,886	Older adults aged 70 to 84 years who participated in the KFACS	76.0 (3.9)	M<br>1374<br>(47.6)<br><br>F<br>1512<br>(52.4)	2016 - 2017	No follow up
116. #301 (Moon., 2019)[46]	Prospective cohort study	South Korea	2392<br>Non-polypharmacy group= 1652<br>Polypharmacy group= 642<br>Hyper-polypharmacy group= 98	Community-dwelling elderly aged between 70 and 84 years	Non-polypharmacy group 75.50 (3.80)<br>Polypharmacy group 76.57 (3.84)<br>Hyper-polypharmacy group 76.66 (4.04)	M<br>742<br>(44.9)<br><br>F<br>910<br>(55.1)	2016 - 2017	No follow up
117. #2514 (Lee., 2021)[67]	Prospective cohort study	South Korea	1292<br>Robust 471<br>Pre-frail 524<br>Frail 297	Community-dwelling older people aged 65 years or older	Robust 72.2 (5.2)<br>Pre-frail 74.6 (6.1)<br>Frail 78.3 (6.3)	M<br>575<br>(44.5)<br>F<br>717<br>(55.5)	December 2014 - December 2017	3 Year follow up
118. #2716 (Jung., 2020)[122]	Longitudinal study	South Korea	2,907	Older adults in community-	Proportion in each age group:	M<br>1383	2016–2017	No follow up

				dwelling residents in urban and rural areas	Age 70-74= 1154 (39.7%)<br>Age 75-79= 1080 (37.2%)<br>Age 80-84= 673 (23.2%)	(47.6)<br><br>F<br>1524<br>(52.4)		
119. #86 (Daou., 2022)[94]	Cross-sectional study	Lebanon	112	Community-dwelling older adults aged ≥ 65 years	73 (12.8)	M<br>39<br>(34.8)<br><br>F<br>73<br>(65.2)	Between September 2019 and February 2020	No follow-up
120. #788 (Yaghi.,2021)[148]	Cross-sectional study	Lebanon	352	60 years of age from Lebanon	73 (67–79)*	M<br>176<br>(50)<br><br>F<br>176<br>(50)	October 2017 - October 2019	No follow-up
121. #2866 (Mino-Leon., 2019)[126]	Secondary analysis of a cohort study	Mexico	1,252	Older adults aged 60 and older who attended one of the 48 primary care centers	68.4 (7.1)	M<br>502<br>(40.1)<br><br>F<br>750<br>(59.9)	2014	No follow up
122. #5965 (Salinas-Rodríguez., 2020)[162]	Cross-sectional study	Mexico	274	Community-dwelling older adults aged ≥60 years in Mexico City	No polypharmacy 80.24 (8.28)<br>Yes polypharmacy 80.82 (8.30)	M<br>122<br>(44.5)<br><br>F<br>152<br>(55.5)	2014-2016	Three semiannual follow-up measurements from 2015 to 2016
123. #3347 (Sanchez-Garcia., 2017)[244]	Cross-sectional study	Mexico	1252	Adults 60 years of age and older affiliated with 48	68.5 (7.2)	M<br>502<br>(40.1)	April - September 2014	No follow-up

				Family Medicine Units (FMU)		F<br>750<br>(59.9)		
124. #380<br>(Hasan., 2018)[128]	Cross-sectional study	Malaysia	135	Community-dwelling older adults (aged ≥ 60 years) in Malaysia	68.89 (6.14)	M<br>62<br>(45.9)<br><br>F<br>73<br>(54.1)	3 months (July - September 2017)	No follow up
125. #2522<br>(Hasan., 2021)[140]	Cross-sectional study	Malaysia	344	Inpatients and outpatients aged 60 years and above	70.71 (7.71)	M<br>189<br>(55.1)<br><br>F<br>154<br>(44.9)		No follow-up
126. #8<br>(Nishtala., 2023)[92]	Secondary analysis of a trial	New Zealand	295	Older adults > 65 years of age	Median 79 (IQR 74- 85)*	M<br>97<br>(33)<br><br>F<br>198<br>(67)	Prescribing trial previously conducted in 2021. The trial was affected by the COVID-19 pandemic public health measures (“lockdown”), which were in place from March 23. 2020, to May 13, 2020 (8 weeks)	6 month follow-up visits (any pending) were conducted by phone
127. #1680<br>(Jamieson., 2023)[172]	Randomized-controlled trial	New Zealand	363	Community-based older adults (≥65 years) from 2 New Zealand district health boards	79.9 (6.9) (range 60–97)	M<br>122<br>(33.6)<br><br>F<br>241<br>(66.4)	September 25, 2018 – October 30, 2020	Follow-up review) in their homes at least 6 months after the first.

								a median follow-up of 254 days
128. #826 (Coelho., 2015)[65]	Cross-sectional study	Portugal	252	Community-dwelling elderly (aged 65 years and over), in three northern Portuguese cities	79.2 (7.3)	M 105 (35.5)<br><br>F 191 (64.5)	January 2015 - November 2015	No follow up
129. #1878 (Dixe., 2023)[111]	Cross-sectional study	Portugal	112	Home-dwelling older adults (65 years) visiting a single primary healthcare centre in the Leiria region in Portugal	76.6 (7.1)	M 52 (46.4)<br><br>F 60 (53.6)	March - April 2022	No follow up
130. #3514 (Polańska., 2016)[78]	Cross-sectional study	Poland	296	Individuals aged 60 years or old confirmed with hypertension	68.8 (8)	M 131 (44.3)<br><br>F 165 (55.7)	January 2015 - November 2015	No follow-up
131. #1977 (Chaouacha., 2022)[182]	Cross-sectional study	Oman	197	Older adults aged ≥65 years attending Wadi Maawil primary healthcare center	72.87 (6.8)	M 75 (38.1)<br><br>F 122 (61.9)	April - June 2021	No follow up
132. #200 (Chen., 2021)[42]	Cross-sectional study	Singapore	292	Older adults aged 65 years and older with Metabolic syndrome (MetS)	70.6 (5.0)	M 123 (42.1)<br><br>F 169 (57.9)	April 2015 - August 2016	No follow up

133. #497<br>(Tan., 2017)[145]	Cross-sectional study	Singapore	115	Patients aged 65 years and older	76.6 (6.5)	M<br>55<br>(47.8)<br><br>F<br>60<br>(52.2)	May 2015 - February 2016	No follow-up
134. #1268<br>(Merchant., 2017)[220]	Prospective cohort study	Singapore	1,051	Older adults aged 65 years and older	Proportion in each age group:<br>65-79 y= 958 (91.2%)<br>≥80 y= 93 (8.8%)	M 450 (42.8)<br>F 601 (57.2)	April 2015 - August 2016	No follow up
135. #3236<br>(Thein., 2018)[246]	Longitudinal study	Singapore	2696<br>Diabetes 486<br>Non-diabetes 2210	Participants aged 55 years of age and older	Diabetes 67.3 (7.5)<br>Non-diabetes 65.6 (7.6)	1693 (62.7)<br>1003 (37.3)	2003–2009	12 years follow up
136. #5680<br>(Jazbar., 2021)[209]	Retrospective cohort study	Slovenia	Slovenia= 2286<br><br>Europe= 37,471	Older adults in Slovenia and other European countries	Proportion in each age group:<br>Slovenia 65-74 y= 1300 (56.9%)<br>75-84 y= 770 (33.7%)<br>≥85 y= 216 (9.4%)<br>Europe<br>65-74 y= 21006 (56.1%)<br>75-84 y= 12834 (34.3%)<br>≥85 y= 3631 (9.7%)	Slovenia M 990 (43) F 1296 (57)<br>Europe M 16833 (45) F 20638 (55)	2011 - 2015	4-year follow-up from 2011 to 2015
137. #77<br>(Rodríguez-Laso.,2023)[85]	Prospective cohort study	Spain	387	Community-dwelling Spaniards 65 years or older from the Toledo Study of Healthy Ageing	72.3 (range 71.8-72.8)	M 163 (42.1)<br>F 224 (57.9)	First (2006-2009), second (2011- 2013), and third (2014-2017) waves of the study	
138. #109<br>(Molist-Brunet., 2022)[96]	Quasi-experimental (uncontrolled pre-post)	Spain	428	Patients aged 65 or older with multimorbidity	85.52 (7.67)	M<br>143<br>(33.4)	June 2019 - October 2020	3 month follow-up

						F<br>285<br>(66.6)		
139. #235 (Mejías-Trueba., 2023)[99]	Cross-sectional study	Spain	83	Polymedicated (>5 active drugs) and 65-year-old patients from reference university hospital	80.5 (range 75.2-85.3)	M 45 (54.2)<br>F 38 (45.8)	October 2020 - April 2021	No follow-up
140. #2215 (Alvarez-Bustos., 2022)[118]	Secondary analysis of a cohort study	Spain	975	Older adults > 65 years	73.14 (4.69)	M<br>423<br>(43.38)<br><br>F<br>552<br>(56.62)	Data analyzed from 2006-2009, 2011-2013, and 2015-2017	No follow up
141. # 374 (GenéHuguet., 2018)[127]	Randomized-controlled trial	Spain	200	Community-dwelling pre-frail elderly patients aged ≥ 80 years	84.5 (3.5)	M<br>71<br>(35.5)<br><br>F<br>129<br>(64.5)	Assessment was from June to September 2016, The intervention lasted from October 2016 to march 2017	March to July 2017
142. #332 (Aznar-Tortonda., 2020)[133]	Cross-sectional study	Spain	621	Patients ≥60 who attended consultations at health centers	73.1 (8.0)	M<br>256<br>(41.2)<br><br>F<br>365<br>(58.8)	Jan 2017-May 2018	No follow up
143. #352 (Vergara., 2019)[135]	Secondary analysis of a cohort study	Spain	865	Community-dwelling older adults ≥70	78.2 (4.9)	M<br>407<br>(47)<br><br>F<br>458<br>(53)	May 2015 - July 2016	2 years of follow-up
144. #362 (Lorenzo-López 2019)[136]	Longitudinal study	Spain	749 at baseline and 537 at 1-year follow-up	Community-dwelling older	75.8 (7.2)<br><br>76.3 (7.0)	M<br>295<br>(39.4)		1-year follow up

				adults aged ≥65 years		F<br>454<br>(60.6)		
145. #257 (Arias-FernÃ¡ndez., 2021)[142]	Cross-sectional study	Spain	392	Non-institutionalized adults aged ≥65 years	Proportion in each age group:<br>65- 74 y= 175 (44.6%)<br>75- 84 y= 152 (38.8%)<br>≥85 y= 65 (16.6%)	M 172 (43.9)<br>F 220 (56.1)		No follow up
146. #1093 (Romera-Liebana.,2018)[150]	Randomized-controlled trial	Spain	347<br>Control 176<br>Intervention 176	Community-dwelling adults aged older than 65 from different areas of Barcelona	Control 77.4 (7.7)<br>Intervention 77.2 (6.8)	**Control**<br>M 45 (25.6)<br>F131 (74.4)<br>**Intervention**<br>M 42 (23.9)<br>F134 (76.1)	12 weeks<br>Between January 2013 and January 2015, participants were rand-omized to the multi-intervention or the control	18 month follo w up
147. #1243 (Machón., 2018)[151]	Cross-sectional study	Spain	527	Non-institutionalized functionally independent older people aged ≥70 years from Gipuzkoa (Spain)	76.22 (5.21)	M<br>237<br>(45)<br><br>F<br>290<br>(55)	August 2016 - October 2017	one-year follow-up
148. #10097 (Díez-Villanueva., 2023)[154]	Cross-sectional study	Spain	500	Elderly (≥75 years) AF patients treated with NOACs for at least 3 months for stroke prevention	81.48 (4.73)	M<br>250<br>(50)<br><br>F<br>250<br>(50)	September 2019–August 2020	No follow up
149. #1954 (Donate-Martinez., 2022)[181]	Longitudinal study	Spain	552	Community-dwelling people aged 70 years or older from the	79.1 (6.3)	M<br>208<br>(37.7)<br><br>F		6 months follow up

				city of Valencia (Spain)		344 (62.3)		
150. #2463 (Blanco-Reina., 2021)[192]	Cross-sectional study	Spain	582	Non-institutionalized adults aged 65 years or more	73.1 (5.5)	M 248 (42.6)<br><br>F 334 (57.4)		No follow-up
151. #2690 (Machon., 2020)[197]	Cross-sectional study	Spain	813	Community-dwelling older people	77.4 (5.0)	M 367 (45)<br><br>F 446 (55)	The data collection was conducted between May 2015 and July 2016 in the first study and from August 2016 to October 2017 in the second one	No follow up
152. #3619 (Serra-Prat., 2016)[204]	Cross-sectional study	Spain	324<br>Robust 104<br>Pre-frail 174<br>Frail 46	Community-dwelling individuals aged 75 years and older	Robust 79.6 (3.07)<br>Pre-frail 80.1 (3.6)<br>Frail 81.1 (3.8)	M 170 (52.5)<br>F 154 (47.5)	January - July 2014	No follow-up
153. #911 (Pérez-Ros., 2020)[225]	Cross-sectional study	Spain	564<br>Robust 91<br>Prefrail/Frail 457	Community-dwelling older people, independent for walking and without impaired cognition	Robust 74.37 (3.23)<br>Prefrail/Frail 76.37 (4.02)	M 208 (37)<br><br>F 356 (63)		No follow-up
154. #812 (García-Esquinas., 2015)[226]	Prospective cohort study	Spain	1750<br>No diabetes 1404<br>Diabetes 346	Noninstitutionalized individuals aged 60 years or older	No diabetes 68.4 (6.3)<br>Diabetes 69.4 (6.4)	M 851 (49)<br><br>F 899 (51)	2008-2012	mean 3.5-year follow-up
155. #5070	Cross-sectional study	Spain	229<br>Fallers 89	Community-dwelling pre-frail	Fallers 77.54 (4.53)	M 68	August - December 2016	No follow up

(Prieto-Contreras., 2023)[251]			Non-fallers 140	and frail people aged 70 years and older	Non-fallers 77.88 (5.07)	(30)<br><br>F<br>161<br>(70)		
156. #3602 (Verloo., 2016)[79]	Secondary analysis of a trial	Switzerland	114	Home-dwelling older adults aged 65 years or older discharged after hospitalization of at least 48 hours	83.2 (7.2)	M<br>40<br>(35.1)<br><br>F<br>74<br>(64.9)	2012	No follow up
157. #3098 (Huang., 2018)[73]	Cross-sectional study	Taiwan	603	Community-dwelling seniors older than 65 years old	70.9 (5.82)	M<br>296<br>(49)<br>F<br>307<br>(51)	February - October 2017	No follow up
158. #701 (Chen., 2021)[87]	Retrospective cohort study	Taiwan	100000	Individuals aged 65 to 100 years old on January 1, 2007	73 (68- 79)*	M<br>48412<br>(48.5)<br><br>F<br>51588<br>(51.5)	2006 - 2015	Mean follow up 7.58
159. #9268 (Chao., 2016)[157]	Prospective cohort study	Taiwan	51	ESRD patients receiving chronic hemodialysis for more than three months	68 (11.8)	M<br>22<br>(43)<br><br>F 29<br>(57)	January 2014 - September 2015	No follow up
160. #1592 (Chi., 2023)[169]	Longitudinal study	Taiwan	49519<br>No glucose lowering drug (GLD)= 21,160<br>oGLD monotherapy= 11901<br>oGLD combination= 14097	Adults older than 40 years with at least 1 time of physician-diagnosed type 2 DM at out-	No glucose lowering drug (GLD) 64.0 (11.2)<br>oGLD monotherapy 65.0 (11.2)	M<br>26083<br>(53)<br><br>F<br>23,436<br>(47)	2008 - 2016	Followed up from the index date, which was 1 year after DM diagnosis,

			Insulin users= 2361	patient, in-patient,<br>or the emergency department	oGLD combination 64.0 (10.8)<br>Insulin users 65.1 (10.9)			until the developmen t of frailty or the end of follow-up, which was Dec 31st, 2017
161. #2361 (Hung., 2021)[189]	Cross-sectional study	Taiwan	205	People aged ≥65 years	Robust 70.8 (5.6)<br>Pre-frail 71.0 (5.1)<br>Frail 71.9 (5.3)	M 95 (46.3)<br>F 110 (53.7)	October 2020 - April 2021	No follow up
162. #3169 (Chen., 2018)[214]	Cross-sectional study	Taiwan	125	Taiwanese men with COPD	77.36 (10.26)	Men only		No follow up
163. #1262 (Pao., 2018)[221]	Cross-sectional study	Taiwan	103 Improved frailty 26 Stable or worsening frailty 77	Older patients aged 65 years and over with chronic diseases	Improved frailty 75.7 (6.6)<br>Stable or worsening frailty 77.6 (5.8)	M 49 (48)<br>F 54 (52)	January 2007 - June 2009	2-year follow-up
164. #4294 (Chen., 2010)[230]	Prospective cohort study	Taiwan	2,238	Individuals aged ≥65 years	73.3 (1.5)	M 1,147 (51.3)<br><br>F 1,092 (48.8)	2003	No follow up
165. #4244 (Chang., 2011)[231]	Cross-sectional study	Taiwan	275	Community-dwelling residents aged Adults aged 65–79 years old in the 31 administrative areas of Toufen	Proportion in each age group:<br>65–68y= 87 (31.6%)<br>69–73 y= 112 (40.7%)<br>74–79 y= 76 (27.6%)	M 127 (46.2)<br><br>F 148 (53.8)		No follow up
166. #284 (Lewis., 2020)[89]	Cross-sectional study	Tanzania	235	People aged ≥ 60 years	73 (65-83)*	M 99 (42.1)<br><br>F	Data collected between February 27 and August 4, 2017	No follow up

						136<br>(57.9)		
167. #6<br>(Derhem., 2023)[27]	Cross-sectional study	Turkey	298	Older adults aged ≥65 years	72.6 (5.85)	M<br>134<br>(45)<br><br>F<br>164<br>(55)	May 2022 - July 2022	No follow up
168. #14<br>(PALA., 2023)[28]	Cross-sectional study	Turkey	400	Older people aged ≥65 years	71.67 (5.49)	M<br>161<br>(40)<br><br>F<br>239<br>(60)	November 2020 - May 2021	No follow up
169. #909<br>(Naharci., 2020)[37]	Case-control study	Turkey	520	Community-dwelling older adults aged 65 years and older	65- 74 y= 179 (34.4)<br>75+ y= 341 (65.6)	M<br>194<br>(37.3)<br>F<br>326<br>(62.7)	May 2017 - April 2019	No follow up
170. #3682<br>(Çakmur., 2015)[81]	Cross-sectional study	Turkey	168	People aged 65 years and older in the rural area of Kars Province	72.7 (7.73)	M<br>78<br>(46.4)<br><br>F<br>90<br>(53.6)	April - September 2014	No follow up
171. #1628<br>(Ozturk., 2023)[107]	Cross-sectional study	Turkey	136<br><br>No polypharmacy= 65<br>Polypharmacy= 71	Patients, who were 65 years and older, and who applied to the geriatric outpatient clinic of a university hospital	No polypharmacy 70 (65-90)*<br>Polypharmacy 74 (65-89)	M<br>52<br>(38)<br><br>F<br>84<br>(62)	October 2020 - October 2021	No follow up
172. #2810<br>(Dogan Varan., 2020)[125]	Cross-sectional study	Turkey	1001	People over the age of 65 years who lived in the	73 (65-94)*	M 378 (37.8)<br>F 623 (62.2)		No follow up

				city center of Burdur and received aid from the Social Assistance Foundation				
173. #1898 (Durmus., 2023)[179]	Cross-sectional study	Turkey	382	Older Turkish adults in the geriatric outpatient clinic	Beers: PIM 72.77 (7.79)<br>Beers: Non-PIM 72.23 (7.22)	M 123 (32.2)<br>F 259 (67.8)	September 2020 - March 2021	No follow up
174. #1899 (Arslan., 2023)[180]	Cross-sectional study	Turkey	263	People over 65 years of age who applied to family medicine outpatient clinics and family health training centers	72.53 (5.83)	M<br>135<br>(51)<br><br>F<br>128<br>(49)	June - August 2018	No follow up
175. #2191 (Sutlu., 2022)[185]	Cross-sectional study	Turkey	383	People over the age of 65 years who lived in the city center of Burdur and received aid from the Social Assistance Foundation	Proportion in each age group:<br>65- 69y= 37 (9.7%)<br>70- 74 y= 76 (19.8%)<br>75- 79 y= 72 (18.8%)<br>80- 84 y= 8 (21.7%)<br>85+ y= 115 (30%)	M<br>104<br>(27.2)<br><br>F<br>279<br>(72.8)		No follow up
176. #2349 (Cakmak., 2021)[187]	Cross-sectional study	Turkey	150	93 women and 57 men with a mean age of 73 +/- 9 years	73 (9)	M<br>57<br>(38)<br>F<br>93<br>(62)	September 2019 - January 2020	No follow up
177. #3755 (Eyigor., 2015)[206]	Cross-sectional study	Turkey	1126<br>Frail= 441<br>Pre-frail= 488<br>Non-frail= 197	Individuals over 65 years of age who presented to the Physical Medicine and Rehabilitation	Proportion in each age group:<br>Frail 65- 74 y= 230 (52.2%); 75-84y= 187 (42.4%); >85 y= 24 (5.4%)	M<br>386<br>(34)<br><br>F<br>740	December 2012 - June 2013	No follow up

				(PMR) outpatient clinics	Pre-frail= 65- 74 y= 345 (70.7%); 75- 84y= 131 (26.8%); >85 y= 12 (2.5%) Non-frail= 65- 74 y= 162 (82.2%); 75- 84y= 131 (26.8%); >85 y= 3 (1.5)	(66)		
178. #5333 (Taci., 2023)[242]	Cross-sectional study	Turkey	261	Individuals aged 65 and over	71.95 (6.14)	M 132 (50.6)<br><br>F 129 (49.4)	1 Jan - 1 Feb 2020	No follow up
179. #5230 (Erdoğan., 2023)[243]	Cross-sectional study	Turkey	144	Participants aged 65 and over	<75y= 94 (65.3) 75- 84y= 39 (27.1) ≥85y= 11 (7.5)	M 57 (39.59) F 87 (60.41)	May 2020 - July 2020	No follow up
180. #3026 (Esenkaya., 2019)[247]	Cross-sectional study	Turkey	300	Turkish older adults	73.85 (7.12)	M 84 (28) F 216 (72)	January 2017 - April 2017	No follow up
181. #2719 (Chumha., 2020)[199]	Cross-sectional study	Thailand	251	Elderly patients (98 males and 153 females) of the Out-Patient Clinic at Maharaj Nakorn Chiang Mai Hospital	60- 69y= 164 (65.3) 70- 79y= 71 (28.2) >80y= 16 (6.3)	M 98 (39)<br><br>F 153 (61)		No follow-up
182. #47 (Cox., 2023)[33]	Secondary analysis of a cohort study	UK	100	Participants aged ≥65 years attending a fracture clinic with a single upper limb fragility fracture	73 (70- 80)*	M 20 (20)<br><br>F 80 (80)	March 2019 - March 2020	baseline to 3-month follow-up, and from 3-month to 6-month follow-up

				from a low-trauma fall				
183. #160 (Österdahl., 2022)[39]	Retrospective cohort study	UK	113779	New users of first-line antihypertensives aged 65 years or over	Fit 71.9 (5.8)<br>Mildly frail 74.9 (0.1) Moderately frail 78.5 (7.5)<br>Severely frail 82.1 (7.6)	M<br>50219<br>(44.1)<br>F<br>63560<br>(55.9)	2007 - 2017	4.1 years [standard deviation (SD) 2.8 years]
184. #214 (McKechnie., 2021)[43]	Retrospective cohort study	UK	1722<br><br>Did not develop frailty 890<br><br>Did develop frailty 91	People aged > 65 on 1st January 2010, registered in CPRD for ≥ 1 year	Did not develop frailty 77.5 (4.2)<br><br>Did develop frailty 79.8 (4.8)	Men only	In 2010–2012, all 3,137 surviving men were invited to attend a 30-year re-examination	3 year follow up
185. #588 (Gulliford., 2017)[62]	Retrospective cohort study	UK	212566<br>Year of entry to study 2001- 2005= 81353<br>Year of entry to study 2006- 2010= 66817<br>Year of entry to study 2011- 2015= 64396	Participants aged ≥80 years	Proportion in each age group:<br>2001- 2005<br>80- 84Y= 23673 (29.1%)<br>85-89 Y= 22130 (27.2%)<br>90- 94Y= 20412 (25.1%)<br>95- 99Y= 12823 (15.8%)<br>100+ Y= 2315 (2.8%)<br><br>2006- 2010<br>80- 84Y= 23132 (34.6%)<br>85-89 Y= 19793 (29.6%)<br>90- 94Y= 15327 (22.9%)<br>95- 99Y= 7989 (12%)<br>100+ Y= 576 (0.9%)<br><br>2011- 2015	2001- 2005<br>M 24251 (29.8) F 57102 (80.2)<br><br>2006- 2010<br>M 22004 (32.9) F 44813 (67.1)<br><br>2011- 2015<br>M 22746 (35.3) F 41650 (64.7)	January 2001 - the 31 December 2015	No follow-up

					80- 84Y= 23060 (35.8%)<br>85-89 Y= 18981 (29.5%)<br>90- 94Y= 14724 (22.9%)<br>95- 99Y= 6905 (10.7%)<br>100+ Y= 726 (1.1%)			
186. #324 (Sheppard., 2020)[103]	Randomized, unblinded, noninferiority trial	UK	569<br>Medication reduction group (N= 282)<br>Usual Care group (N=287)	Participants were aged 80 years and older, had a baseline systolic blood pressure lower than 150mmHg, and were prescribed 2 or more antihypertensive treatments for at least 12 months	Medication reduction group 84.6 (3.3)<br>Usual Care group 85 (3.5)	M<br>282<br>(49.6)<br><br>F<br>287<br>(50.4)	March 20, 2017 - September 30, 2018	12 week follow-ups conducted
187. #2441 (Houghton., 2021)[120]	Retrospective cohort study	UK	Non-frail 92<br>Frail 98	Patients aged ≥50	Non-frail 70.3 (9.5)<br>Frail 77 (10.2)	M 126 (66.3)<br>F 64 (33.7)	February 2018 - April 2019	No follow up
188. #7851 (Cheong., 2023)[160]	Cross-sectional study	UK	529,095	Patients aged ≥65 at their first GP consultation between 1 January and 31 December 2018	75 (7.4)	M<br>244,285<br>(46.2)<br><br>F<br>284,810<br>(53.8)	1 January - 31 December 2018	No follow up
189. #1151 (Gafoor., 2019)[165]	Retrospective cohort study	UK	153,304	Adults aged 80 years and older	83 (range 80-114)	M<br>56,18<br>(39)<br><br>F<br>87,217<br>(61)	2006-2015	1 year follow-ups

190. #1913 (Elhussein., 2022)[194]	Retrospective cohort study	UK	110225 Frailty Cohort	People aged > 65 on 1st January 2010, registered in CPRD for ≥ 1 year	78.74 (7.4)	M 45272 (41.1) F 64953 (58.9)	People aged>65 on 1st January 2010, registered in CPRD for≥1 year	No follow up
191. #2952 (Porter., 2019)[211]	Prospective cohort study	UK	1154	Cognitively impaired participants, aged 65 years or older	78.8 (7.4)	M 437 (37.9) F 717 (62.1)	2008 - 2011	PIMS: 2-year follow-up and mortality: 8-year follow-up
192. #708 (Ravindrarajah., 2017)[235]	Retrospective cohort study	UK	265 225	Men and women aged 80 years and over	M 82.8 (4.12) F 84.5 (5.28)	M 116401 (43.9) F 148824 (56.1)	2001 - 2014	No follow-up
193. #7822 (ThreapletonCJD., 2020)[241]	Retrospective cohort study	UK	100	People with polypharmacy aged 65–84 years	74 (6)	M 50 (50) F 50 (50)	2016	No follow up
194. #43 (Chaitoff., 2023)[32]	Cross-sectional study	USA	400	Adults ≥65 years with frailty	75	M 157 (39.2) F 243 (60.8)	2005 - 2018	No follow up
195. #128 (Thomas., 2022)[37]	Secondary analysis of a cohort study	USA	5,533	Community dwelling adults ≥60 years old	70.1 (7)	M 2705 (49) F 2828 (51)	2011– 2012, 2013–2014, and 2015–2016 data	No follow-up
196. #145 (Callahan., 2022)[50]	Cross-sectional study	USA	16973	Patients who were ≥ 65 years of age with at least two cumulative visit diagnosis codes	75.2 (6.9)	M 7819 (46.1) F 9154 (53.9)	2020	No follow up

				for type 2 diabetes				
197. #139 (Bergen., 2022)[53]	Retrospective cohort study	USA	Frailty index model= 13,370; functional domain models= 13125; Frailty phenotype model= 7560	Community-living respondents 65 years of age and older	Proportion in each age group: Frailty Index 65- 69y= 4564 (51.8%) 70- 74y= 1887 (21.4%) 75- 79y= 1146 (13%) 80- 84y= 760 (8.6%) ≥85 y= 446 (5.1%)	M 4068 (46.2) F 4735 (53.8)	Six biennial waves (2006–2016)	Follow-up data from later waves (through 2016)
198. #3440 (Ballew., 2016)[75]	Cross-sectional study	USA	4987 Not frail 4646 Frail 341	Community-dwelling older men and women	Non-Frail 75.4 (5.1) Frail 78 (5.6)	M 2205 (44.2) F 2782 (55.8)	2011-2013	No follow up
199. #147 (Brown., 2022)[98]	Randomized-controlled trial	USA	121	60 years or older with a diagnosis of either major depressive disorder (MDD) or persistent depressive disorder (PDD)	70.82 (7.71)	M 43 (36) F 78 (64)		Assessed at pretreatment baseline, 8 weeks, 6 and 12 months
200. #334 (Cil., 2019)[104]	Longitudinal cohort study	USA	14208	Community-living respondents aged 65 years and older			2006 - 2014	Average follow-up was 5.4 years
201. #316 (Shmuel., 2019)[132]	Prospective cohort study	USA	1697	White and African American adults aged 50-95 residing in Johnston County, North Carolina	69 (9)	M 567 (33) F 1130 (67)	2006-2010 for polypharmacy measure, 2006-2010 and 2013-2015 for frailty measure	conducted over 2 consecutive visits (2006-2010 and then 2013-2015)

202. #338 (Sanghai., 2020)[134]	Prospective cohort study	USA	460	Older adults ≥65 with atrial fibrillation who were treated with direct oral anticoagulants (DOACs)	Recommended dose of DOAC 73.8 (6.3)<br>Off-label dose of DOAC 76.6 (6.5)<br>Over-dosed DOAC 76.1 (7.0)<br>under-dosed DOAC 76.8 (6.4)	M<br>236<br>(51)<br><br>F<br>224<br>(49)	2016-2018	No follow up
203. #1113 (LaCroix., 2008)[164]	Prospective cohort study	USA	25,378	Non-frail women 65–79 years old at baseline	65- 69y= 13085 (51.6)<br>70- 79y= 12293 (48.4)	Women only	1993 - 1998	3 year follow-up
204. #1293 (Lee., 2018)[167]	Cross-sectional study	USA	4551	Adults age 65–90	73.3	M<br>2071<br>(45.5)<br><br>F<br>2480<br>(54.5)	2014/2015	No follow up
205. #1593 (Sargent., 2023)[170]	Prospective cohort study	USA	80	Participants were 60 years or older and living independently in low-income subsidized housing community-based settings	69 (9.2)	M<br>36<br>(44.3)<br><br>F<br>44<br>(55.7)	2021	No follow up
206. #1770 (Alqahtani., 2023)[175]	Cross-sectional study	USA	328	Individual aged between 65 and 85 years	72.6 (5.8)	M<br>148<br>(45)<br><br>F<br>180<br>(55)	cross-sectional analysis was performed using data from the Midlife in the United States (MIDUS 2): Biomarker Project, 2004–2009	No follow up

207. #1968 (Sanghai., 2022)[195]	Retrospective cohort study	USA	308,664	Patients with NVAF and CHA2DS2VASC ≥2 receiving care	77.7 (9.6)	M 302182 (97.9)<br>F 6482 (2.1)	Patients with NVAF and CHA2DS2VASC ≥2 receiving care between Feb- ruary 2010 and September 2015	No follow up
208. #968 (Lakey., 2012)[223]	Secondary analysis of a cohort study	USA	27,652	Women aged 65 to 79, not frail at baseline	65- 69y= 14156 (51.2)<br>70- 79 y= 13496 (48.8)	Women only	1993 - 1998	3-year follow-up
209. #956 (Crentsil., 2010)[224]	Cross-sectional study	USA	975	Community-dwelling women aged ≥65 years who self-reported difficulty in at least 2 of 4 domains of physical functioning	78.3 (8.1)	Women only	1992 - 1995	No follow-up
210. #1920 (Salazar., 2022)[112]	Cross-sectional study	Venezuela	201	Subjects of both genders, over 60 years old that went to the Internal Medicine outpatient clinic of the Hospital General del Sur	Proportion in each age group:<br>60- 69y= 120 (59.7%)<br>70- 79 y= 58 (28.9%)<br>≥ 80 y= 23 (11.4%)	M<br>72<br>(36)<br><br>F<br>129<br>(64)	March 1, 2018 - June 1, 2018	No follow up
211. #2145 (Anh., 2022)[138]	Cross-sectional study	Vietnam	396	Older adults at the Geriatric clinic of Gia Dinh People's hospital in Ho Chi Minh City	72.6 (7.6)	M<br>150<br>(37.9)<br><br>F<br>246<br>(62.1)	2019 - May 2020	No follow up
212. #3007 (Nguyen., 2019)[212]	Cross-sectional study	Vietnam	523	Older adults (≥60 years old) residing in Soc Son district,	Non-frailty 67.4 (4.9)<br>Pre-frailty 72.6 (7.7)	M<br>154<br>(30)	February - April 2017	No follow up

				northern Vietnam	Frailty 76.7 (9.2)	F 358 (70)		
213. #1974 (Dautzenberg., 2022)[114]	Retrospective cohort study	Netherland	431	All transcatheter aortic valve replacement (TAVR) referred to the geriatric outpatient clinic for a geriatric preoperative screening	80.8 (6.2)	M 191 (44)<br>F 240 (56)	January 2014 - December 2019	Follow-ups conducted 3 months after TAVR
214. #2532 (de Breij., 2021)[121]	Prospective cohort study	Netherland	569	Older adults aged 75 years and over	79.4 (5.4)	M 231 (40.6)<br>F 338 (59.4)	Data from 2008-2009 and 2011-2012 were used	3 year follow-up
215. #618 (Hoeksema., 2017)[131]	Cross-sectional study	Netherland	1026	Dutch community-living elderly (≥75 years of age)	80 (77–84)*	M 424 (41)<br>F 602 (59)	June 2015 - November 2015	No follow up
216. #632 (Mertens.,2018)[146]	Case-control study	Netherland	Multi-drug users (MDD) 188<br>Non Multi-drug users (MDD) 230	65 years of age, home dwelling, and using at least five chronic drugs	MDD users 80.5 (76-85)*<br>Non MDD users 76 (71-81)*	Multi-drug users (MDD) M 62 (33) F 126 (67)<br>Non Multi-drug users (MDD) M 109 (47) F 121 (53)		
217. #8739 (vanKempen., 2015)[158]	Cross-sectional study	Netherland	587	Patients aged 70 years and older and registered in the six family practices	77 (5)	M 257 (44) F 330 (56)	February 2010 - August 2011	No follow up
218. #2355 (de Breij., 2021)[188]	Prospective cohort study	Netherland	1477	Older adults aged 65 years and over who participated in the study in 2008–2009	Men 75.5 (7.6)<br>Women 77.2 (8.2)	M 630 (42.7)<br>F 847 (57.3)	2008–2009 and linked their data to register data on mortality up to 2015	Mortality status at six year follow-up
219. #2723 (Oetsma., 2020)[200]	Cross-sectional study	Netherland	80	Patients ≥65 years with	74.6 (5.9)	M 27 (34)<br>F 53 (66)	January - April 2018	No follow up

				rheumatoid arthritis				
220. #480 (Kleipool., 2019)[238]	Retrospective cohort study	Netherland	244,328<br>lipid lowering drug (LLD) Cardiovascular disease (CVD)<br>LLD and with CVD= 38049<br>No LLD and with CVD= 17260<br>LLD and without CVD= 67428<br>No LLD and without CVD= 121563	Adults ≥70 years	LLD and with CVD 77.3 (6.0)<br>No LLD and with CVD 81.2 (7.6)<br>LLD and without CVD 76.0 (5.5)<br>No LLD and without CVD 77.3 (6.9)	LLD and with CVD M 21384 (56.2) F 16665 (43.8)<br>No LLD and with CVD M 7370 (42.7) F 9890 (57.3)<br>LLD and without CVD M 30275 (44.9) F 37153 (55.1)<br>No LLD and without CVD M 48261 (39.7) F 73302 (60.3)	2011–2015	
221. #3271 (Kleipool., 2018)[245]	Prospective cohort study	Netherland	1432<br>Yes CVD= 148<br>No CVD 1284	Older adults (aged 65- 88yrs)	Yes CVD 78.3 (5.9)<br>No CVD 75.4 (6.6)	Yes CVD M 78 (53) F 70 (47)<br>No CVD M 616 (48) F 668 (52)	1998-2012	followed over a period of 17 years during which follow-up measurements were collected approximately every three years. For the present study, we used data of 5 follow-up cycles

222. #8410 (Hoogendijk., 2013)[250]	Cross-sectional study	Netherland	102	People aged 65 and over from a primary care practice in Amsterdam	78.6 (7.1)	M 44 (43.1)<br>F 58 (56.9)	October - December 2009	No follow up
223. #2726 (Ambrož., 2020)[254]	Repeated cross-sectional cohort study	Netherland	4819	Patients with type 2 diabetes	Proportion in each age group:<br><60y= 1620 (34%)<br>60- 69 y= 1585 (33%)<br>70- 79 y= 1068 (22%)<br>≥80y= 546 (11%)	M 2560 (53)<br>F 2259 (47)	2007–2014	

*= median IQR; F = Female; M = male;

Supplementary Table S4. Studies reporting mean or median number of medications in older adults with different frailty status

Study ID		Non-Frail	Pre-frail	Frail	P-value (any test used)
**Mean number of medications/drugs (±SD)**					
Woo., 2015*[234]		N/A	2.9 (±2.2)	4.3 (±2.9)	N/A
Moon., 2019*[46]		2.9 (±2.57)	3.71 (±2.97)	4.582 (±3.03)	N/A
Eyigor., 2015*[206]		2.5 (±1.99)	3.31 (±2.32)	4.62 (±2.74)	N/A
Pérez-Ros., 2020*[225]		3.91 (2.61)	4.86 (3.09) pre-frail/frail		0.006 (STT)
Aprahamian., 2018*[25]		2.56 (2.0)	3.88 (2.7)	5.01 (2.7)	<0.001 (KW)
Arslan., 2023*[180]		4.25 (±2.80)	N/A	5.14 (±2.48)	0.008 (STT)
Jung 2020*[122]		3.9 (±0.08)	4.7 (±0.09)	5.7 (±0.23)	<0.001 (AN)
Gutiérrez-Zúñiga., 2023*[30]		1.3 (1.3)	3.2 (2.2)	6 (3.2)	<0.001 (AN)
Gnjidic., 2012*[83]		3.3 (2.5)	4.5 (2.9)	6.1 (3.4)	<0.0001 (MW)
Vendola 2023*[109]		3.2	N/A	6.4	<0.001 (MW)
Verloo., 2016*[79]		5.20 (2.4)	N/A	6.61 (2.7)	0.019 (STT)
Esenkaya., 2019*[247]		4.35 (±2.72)	5.41 (±2.49)	6.86 (±2.99)	<0.001 (KW)
Miguel., 2012*[84]		4 (2)	5 (2)	7 (2)	N/A
Rhalimi et al., 2021*[41]		4.7 (±3.1)	N/A	7.5 (±3.8)	N/A
Jung., 2016*[252]		5.4 (3.7)	6.4 (4.4)	9 (4.3)	0.014 (KW)
Houghton 2021*[120]		7.1 (±3.4)	N/A	9.4 (±3.9)	N/A
Ballew., 2016*[75]		8.8 (±4.6)	N/A	10.5 (±5)	N/A
Takeuchi., 2018*[215]		9.3 (±3.2)	9.9 (±3.7)	12.1 (±3.8)	<0.001 (AN)
Tabue-Teguo., 2023*#^[173]		14.3 (±18.9)#	N/A	15.6 (±16.8)#	N/A
		7.0 (±11.9)*	N/A	8.3 (±11.7)*	N/A
		7.4 (±9.8)^	N/A	7.3 (±7.3)^	N/A
Braun., 2019*[72]	FP	4.3 (±3.3)	N/A	7 (±4.8)	<0.001 (MW)
	FFI	3.7 (±2.8)	N/A	7.2 (±4.3)	<0.001 (MW)
Ribeiro, 2022*[115]	TFI	6.96 (±1.98)	N/A	8.58 (±2.65)	N/A
**Median number of medications/drugs (IQR/range)**					

Kume., 2021*[36]		2 (4)	3 (4)	3 (3.5)	0.87 (KW)
Uragami., 2021*[58]		3 (2- 5)	4 (3- 6)	5 (3- 7)	<0.001 (KW)
Moulis., 2015*[233]		N/A	N/A	6 (5)	N/A
Serra-Prat., 2016*[204]		5 (3)	6 (3)	8 (3)	<0.001 (KW)
Elhussein., 2022*[194]		N/A	N/A	10 (8-14)	N/A
Thiruchelvam., 2021@[49]	2003-2017	N/A	N/A	13-14 (10-19)	N/A
Callahan., 2022*[50]		9 (7-13)	12 (9-16)	16 (12-20)	N/A
Doğan Varan, 2020*[125]	FFI	3 (0-17)	4 (0-13)	5 (0-17)	<0.001 (MW)
	EFS	5 (0–14)	5 (0–14)	6 (0–17)	<0.001 (MW)
Ribeiro, 2022*[115]	FP	6.5 (6-8)	7.5 (6-9)	8 (6-10)	0.279 (STT)

AN = One-way ANOVA test; **EFS**: Edmonton Frailty Scale; **FFI**: Fried Frailty Index; **FP**: Frailty Phenotype; **IQR** = Interquartile Range; **KW** = Kruskal-Wallis Test; **MW** = Mann-Whitney U Test; **SD** = Standard Deviation; **STT** = Student T-Test; **TFI:** Tilburg Frailty Index; *= Prescribed medications; ^ = OTC medications; # = prescribed medications + OTC medications; @ = prescribed medications in older women

Supplementary Table S5. Distribution of patients across frailty groups based on medications prescribed

Study ID	Medication	Non-Frail (%)	Pre-frail	Frail (%)
(Chaitoff., 2023)[32]	Diabetes medication	N/A	N/A	135/400= 33.8
	Hypertension medication	N/A	N/A	301/400= 75.3
	Statin	N/A	N/A	182/400= 45.5
	Aspirin	N/A	N/A	124/226= 54.9
(Ballew., 2016)[75]	Any hypertension medication	3516/4646= 75.7	N/A	298/341= 87.4
	Statin use	2456/4646= 52.9	N/A	187/341= 54.8
	Anticoagulant use	341/4646= 7.3	N/A	41/341= 12
	Aspirin use	3207/4646= 69	N/A	245/341= 71.8
	Antianxiety use	294/4646= 6.3	N/A	32/341= 9.4
	Antipsychotic use	13/4646= 0.3	N/A	2/341= 0.6
	Hypnotic/Sedative	335/4646= 7.2	N/A	34/341= 10
	Anticonvulsant use	83/4646= 1.8	N/A	8/341= 2.3
	Antidementia	75/4646= 1.6	N/A	12/341= 3.5
	Antidepressant	38/4646= 0.8	N/A	4/341= 1.2
	CNS Altering use	296/4646= 6.4	N/A	36/341= 10.6
	Diabetic meds	931/4646= 20	N/A	106/341= 31.1
	Metformin	537/4646= 11.6	N/A	51/341= 15
	Beta blocker	1575/4646= 33.9	N/A	146/341= 42.8
	ACEI: angiotensin-converting-enzyme inhibitor	1101/4646= 23.7	N/A	92/341= 27
	ARB: angiotensin receptor blockers	505/4646= 10.9	N/A	45/341= 13.2

	Aldosterone antagonist	105/4646= 2.3	N/A	18/341= 5.3
	Loop diuretics	391/4646= 8.4	N/A	84/341= 24.6
	Thiazide diuretics	1462/4646= 31.5	N/A	102/341= 29.9
	K+ sparing diuretics	330/4646= 7.1	N/A	33/341= 9.7
	Digoxin use	73/4646= 1.6	N/A	15/341= 4.4
	Glucocorticoid use	124/4646= 2.7	N/A	20/241= 5.9
	Bisphosphonates	209/4646= 4.5	N/A	18/341= 5.3
(Jankowska-Polańska., 2016)[78]	Angiotensin-converting enzyme inhibitors taken	47/98= 48	N/A	85/198= 42.9
	β-Blockers	21/98= 21.4	N/A	61/198= 30.8
	Angiotensin II receptor antagonist	2/98= 2	N/A	15/198= 7.6
	Thiazide-like diuretics	23/98= 23.5	N/A	32/198= 16.2
	Calcium antagonists	34/98= 34.7	N/A	57/198= 28.8
(Ribeiro 2022)[115]	ACEI/ARB (Frailty phenotype)<br>(TFI)	11/12= 92<br>48/54= 89	60/64= 94	25/30= 83<br>44/48= 92
	b-blocker (Frailty phenotype)<br>(TFI)	12/12= 100<br>50/54= 93	59/64= 92	29/30= 97<br>46/48= 96
	Diuretics (Frailty phenotype)<br>(TFI)	12/12= 100<br>51/54= 94	58/64= 91	29/30= 97<br>45/48= 94
(Liu., 2023)[178]	Metformin	213/340= 62.6	N/A	32/82= 39
	Sulfonylureas	60/340= 17.6	N/A	14/82= 17.1
	Glinide	17/340= 5	N/A	6/82= 7.3
	Glucosidase inhibitor	140/340= 41.2	N/A	40/82= 48.8

	Thiazolidinedione	21/340= 6.2	N/A	8/82= 5.6
	DPP-4 inhibitors	27/340= 7.9	N/A	3/82= 3.7
	SGLT2 inhibitors	19/340= 5.6	N/A	4/82= 4.9
	GLP-1 agonist	7/340= 2.1	N/A	0/82= 0
	Insulin	100/340= 29.4	N/A	21/82= 25.6
(Koponen., 2013) [208]	Any analgesic	96/237= 40.5	163/299= 54.5	47/69= 68.1
	1 analgesic	79/237= 33.3	119/299= 39.8	32/69= 46.4
	2-4 analgesics	17/237= 7.2	44/299= 14.7	15/69= 21.7
	Daily analgesic use	6/237= 2.5	39/299= 13	10/69= 14.5
	As-needed analgesic use	92/237= 38.8	143/299= 47.8	45/69= 65.2
	Acetaminophen	45/237= 19	103/299= 34.4	37/69= 53.6
	NSAIDs	58/237= 24.5	72/299= 24.1	15/69= 21.7
	Traditional analgesics	51/237= 21.5	57/299= 19.1	12/69= 17.4
	Coxibs	1/237= 0.4	8/299= 2.7	2/69= 2.9
	Opioids	9/237= 3.8	31/299= 10.4	12/69= 17.4
(Chen., 2010) [230]	Hypnotics	86/1302= 6.8	110/753= 12.3	21/183= 19.3
	Analgesics for arthritis	117/1302= 9.5	193/753= 21.6	31/183= 28.4
	Other analgesics	61/1302= 5	74/753= 8.3	14/183= 12.9
	Sedatives	56/1302= 4.5	58/753= 6.5	9/183= 8.6
(Österdahl., 2022)[39]	ACEI/ARB angiotensin-converting enzyme inhibitor or angiotensin receptor blocker	19,302/64145= 30.1	Mi: 12,478/36373 = 34.3; Mo: 4170/11904 = 35<br>S: 504/1357 = 37.1	
	Calcium channel blockers	32,123/64145 = 50.1	Mi: 16,667/36,373 = 45.8 ; Mo: 5210/11904 = 43.8	

			S: 573/1357 = 42.2
	Thiazide diuretics	12,720/64145 = 19.8	Mi: 7228/36,373=19.9 ;Mo: 2524/11904=21.2;S: 280/1357=20.6
	Bisphosphonate ever prescribed	5032/64145 = 7.8	Mi: 280/1357 = 20.6; Mo: 3798/11904 = 31.9; S: 609/1357 = 44.9
#588 (Gulliford., 2017)[62]	Prevalent statin use for Year of entry to study 2001- 05	1956/36500 = 5.4	Mi: 3458/28373 = 12.2; Mo: 1993/12691 =15.7;S: 684/3789 = 18
	Prevalent statin use for Year of entry to study 2006- 10	3159/17190 = 18.4	Mi: 8855/25744=34.4;Mo: 7478/16811=44.4;S: 3801/7072= 53.7
	Prevalent statin use for Year of entry to study 2011- 15	3224/12394 = 26	Mi: 10587/23052=45.9;Mo: 10954/18814=58.2;S: 6982/10136=68.9
#7851 (Cheong., 2023)[160]	Prescribed at least one anticholinergic medicine	78515/255402= 30.7	Mi: 107317/185902 = 57.7; Mo: 50495/69867 = 72.3; S: 14523/17924 = 81
#1808 (Nishimura., 2023)[176]	Warfarin	189/980= 19.3	Mi: 709/3967 = 17.9; Mo: 745/4385 = 17; S: 602/3253 = 18.5
	Apixaban	172/980= 17.6	Mi: 849/3967 = 21.4; Mo: 1050/4385 = 24; S: 903/3253 = 27.8
	Dabigatran	126/980= 12.9	Mi: 400/3967 = 10.1; Mo: 449/4385 = 10.2; S: 283/3253 = 8.7
	Edoxaban	142/980= 14.5	Mi: 566/3967 = 14.3; Mo: 667/4385 = 15.2; S: 520/3253 = 16
	Rivaroxaban	351/980= 35.8	Mi: 1443/3967 = 36.4; Mo: 1474/4385 = 33.6; S: 945/3253 = 29.1
	Antihypertensives	575/980= 58.7	Mi: 2908/3967 = 73.3; Mo: 3530/4385 = 80.5;S: 2757/3253= 84.8
	Antidiabetic drugs	68/980= 6.9	Mi: 524/3967 = 13.2; Mo: 710/4385 = 16.2;S: 579/3253 = 17.8
	Nitrates	15/980= 1.5	Mi: 223/3967 = 5.6; Mo: 486/4385 = 11.1; S: 628/3253 = 19.3
	Statins	139/980= 14.2	Mi: 930/3967 = 23.4; Mo: 1347/4385 = 30.7; S: 1096/3253 = 33.7
	NSAIDs	205/980= 20.9	Mi: 1327/3967 = 33.5; Mo: 1920/4385 = 43.8; S: 1938/3253=59.6
	Antiarrhythmic drugs	222/980= 22.7	Mi: 826/3967 = 20.8; Mo: 964/4385 = 22; S: 785/3253 = 24.1
	Antiplatelet drugs	75/980= 7.7	Mi: 776/3967 = 19.6; Mo: 1309/4385 = 29.9; S: 1350/3253 = 41.5
	Other lipid-lowering drugs	35/980= 3.6	Mi: 191/3967 = 4.8; Mo: 294/4385 = 6.7; S: 221/3253 = 6.8
	Antidepressants	11/980= 1.1	Mi: 163/3967 = 4.1; Mo: 396/4385 = 9; S: 566/3253 = 17.4

	Antacid	110/980= 11.2	Mi: 1153/3967 = 29.1; Mo: 2130/4385 =48.6; S: 2220/3253 =68.2

Mi = Mild Frailty; **Mo** = Moderate Frailty; **S** = Severe Frailty

Supplementary Table S6. Distribution of polypharmacy based on frailty status

Study ID	Polypharmacy (definition)	Non-Frail (%)	Pre-frail (%)	Frail (%)	P-value (any test used)
(Aprahamian., 2018)[25]	5-9 medications/day	30/199 = 15.1	132/331= 39.9	57/99 = 57.6	<0.001 (CS)
(Pala., 2023)[28]	≥5 medications	8/125 = 6.4	15/177 = 8.4	8/125 = 6.4	0.002 (CS)
(Gutiérrez-Zúñiga., 2023)[30]	Not mentioned.	8/295 = 2.7	62/190 = 32.6	29/38 = 76.3	<0.001 (CS)
(Sobhani., 2022)[34]	≥3 medications	63/242 = 26	388/1141 = 34	69/146 = 47.2	<0.01 (MR)
(Larsen., 2020)[57]	≥9 medications	25357/75921= 33.4	28920/64210= 45	58075/110297= 52.7	
	≥5 medications	54717/75921= 72.1	52368/64210= 81.6	94295/110297= 85.5	
(Kume et al., 2021)[68]	≥6 medications	14/138= 10.1	32/163= 19.6	6/12= 50	0.001 (KW)
	≥5 medications	26/138= 18.8	48/163= 29.4	7/12= 58.3	0.004 (KW)
(Kurnat-Thoma., 2022)[37]	≥5 prescription medications in past 30 days	660/2619 = 25	1105/2432 = 45	319/482 = 66	<0.01 (MR)
(Chen., 2021)[42]	≥5 medications	51/153 = 33.3	51/118 = 43.2	15/21 = 71.4	<0.01 (BR)
(Ekram., 2022)[54]	≥ 5 medications	2392/11246 = 21.3	2464/7447 = 33.1	232/421 = 55.1	<0.001 (CS)
(Midão., 2021)[59]	Concurrent use of ≥5 medications/day	1836/9976 = 18.4	4076/11680 = 34.9	1804/3037 = 59.4	N/A
(Lee., 2021)[67]	≥5 medications	73/471 = 15.5	118/524 = 22.5	107/297 = 36	<0.001 (LRA)
(Alves., 2020)[71]	≥5 medications	41/181 = 22.7	104/323 = 32.2	39/76 = 51.3	<0.001 (LRM)
(König., 2018)[76]	≥5 medications	N/A	129/317 = 40.7	4/317 = 1.3	<0.001 (CS)
(Closs., 2016)[77]	≥5 medications	40/143 = 28.4	108/266 = 40.6	64/112 = 57.1	<0.001 (MR) (Frail vs. Non-frail)
					0.010 (MR) (Pre-frail vs. non-frail)
(Oliveira., 2022)[86]	≥5 medications	N/A	63/75 = 84	N/A	0.001 (CS)
(Xu., 2022)[95]	≥ 5 medications simultaneously	1/17= 6	3/62= 4.8	44/152= 29	0 (CS)

(Salaffi., 2021)[100]	5-9 medications mean(SD)	4.08 (1.02)	6.00 (1.64)	10.31 (1.84)	N/A
(Ambagtsheer 2020)[102]	≥5 medications (FP)	16/59 = 27.1	43/129 = 33.3	29/40 = 72.5	N/A
	≥5 medications (AFI)	6/51 = 11.8	17/66 = 25.8	65/111 = 58.6	N/A
(Öztürk 2023)[107]	≥5 medications daily	42/98 = 42.9	17/24 = 70.8	12/14 = 85.7	<0.001 (BR)
(Salazar 2022)[112]	≥5 medications simultaneously	17/99 = 17.2	13/40 = 32.5	23/62 = 37.1	0.01 (CS)
(O'Donoghue 2021)[119]	≥5 medications excluding OTC medications.	12/44 = 27.3	14/26 = 55.7	25/34 = 73.5	<0.001 (CS)
(Jung 2020)[122]	≥5 medications	320/1312 = 24.4	491/1366 = 36.0	125/229 = 55.0	<0.001 (LRA)
(Buttery.,2015) [152]	≥5 prescription medications in prior 7 days	472/1139 = 41.5	340/659 = 51.7	38/45 = 85.8	N/A
(Panda., 2020)[155]	≥5 prescription medications	N/A	13/51 = 26	37/76 = 74	0.0009 (CS)
(Arakawa Martins., 2019)[163]	≥5 medications	23/328 = 7	40/254 = 15.7	24/91 = 26.4	<0.001 (CS)
(Melo Filho., 2020)[196]	≥5 medications per day	89/325= 27.4	427/ 1120= 38.1	141/271= 52	N/A
(Tembo., 2020)[201]	≥ 5 medications	38/99 = 39.2	122/199 = 61.6	53/62 = 88.3	<0.001 (CS)
(Nguyen., 2019)[212]	≥5 medications	3/65 = 4.6	45/336 = 13.4	24/111 = 21.6	<0.01 (CS)
(Meng., 2019)[218]	> 5 medications	4/25 = 16	25/63 = 39.7	8/13 = 61.5	0.023 (CS)
(Merchant., 2017)[220]	≥5 long-term medications	108/597 = 18.1	116/389 = 29.8	27/65 = 41.5	<0.001 (CS)
(Saeidimehr., 2021)[228]	>5 medications	124/230= 53.9	53/230= 23	53/230 = 23	0.158 (CS)
(Hironaka., 2020)[240]	≥5 medications	36/237 = 15.2	111/380 = 29.2	27/65 = 41.5	<0.001 (CS)
(Sanchez-Garcia., 2017)[244]	≥3 medications	187/482 = 33.1	289/630 = 51.2	89/140 = 15.8	<0.001 (CS)
**Based on No frailty and Yes frailty**					
(Ye., 2022)[26]	≥5 medications simultaneously	288/804 = 35.9	N/A	520/979 = 53.1	<0.001 (CS)
(Thiruchelvam., 2021)[40]	5–9 unique medications	820/2083 = 39.4	N/A	1258/6913 = 18.2	N/A

(Braun., 2019)[72]	≥5 medications	88/212 = 42 (Physical Frailty Phenotype)	N/A	32/46 = 70	0.001 (CS)
		58/178 = 33 (Frailty Index)	N/A	62/80 = 78	<0.001 (CS)
(Wang., 2015)[82]	≥5 different prescription medications	N/A	N/A	156/532 = 29.3	0.261 (CS)
(Daou 2022)[94]	≥ 5 medications /day	20/96 = 20.8	N/A	9/16 = 56.3	0.003 (CS)
(Dixe 2023)[111]	≥5 medications	27/68 = 40	N/A	41/68 = 60	0.215
(Dautzenberg 2022)[114]	≥5 medications	201/276 = 73	N/A	139/155 = 90	< 0.001 (CS)
(Anh 2022)[138]	5 medications (minimum)	246/300 = 82	N/A	84/96 = 87.5	N/A
(Kim., 2019)[144]	≥3 medications daily	136/521 = 49.3	N/A	140/287 = 50.7	<0.001 (TT)
(Lee.,2020)[149]	≥5 medications	295/1123 = 26.3	N/A	79/141 = 56	<0.001 (CS)
(Tabue-Teguo., 2023)[173]	5 medications (minimum)	45/80 = 56.2	N/A	27/35 =77.1	0.033 (QP)
(Krawariti., 2023)[177]	≥4 chronic prescription medications	19/21 = 88.9	N/A	31/32 = 96.4	0.384 (CS)
(Liu., 2023)[178]	No definition provided	203/340 = 59.7	N/A	68/82 = 82.9	< 0.001 (CS)
(Arslan., 2023)[180]	≥5 medications daily	59/142 = 41.5	N/A	73/121 = 60.3	0.003 (CS)
(Athuraliya., 2022)[184]	≥5 medications	795/3550 = 22.3	N/A	372/684 = 54.3	N/A
(de Breij., 2021)[188]	≥5 medications	482/1477 = 32.6	N/A	987/1477 = 66.8	N/A
(O'Donovan., 2021)[190]	≥5 medications	3045/88084 = 3.5	N/A	2185/9607 = 22.8	<0.01 (CS)
(Setiati., 2021)[191]	≥5 medications daily	509/738 = 69	N/A	134/170 = 78.8	0.01 (CS)
(Elhussein., 2022)[194]	≥ 10 medications	N/A	N/A	35318/475371 = 7.4	N/A
(Shim., 2020)[198]	≥5 prescribed medications	668/1917= 34.8	N/A	123/211= 58.3	0.005 (TT & CS)
(Wong., 2020)[216]	>5 prescribed medications	N/A	N/A	80/91 = 87.9 (EBC)	N/A

				311/387 = 80.4 (ABC)<br>42/56 = 75 (BBC)	
		N/A	N/A	1221/1553 = 78.6 (EM)<br>649/1319 = 49.2 (AM)<br>144/216 = 66.7 (BM)	N/A
(Thompson., 2018)[237]	≥5 medications (FP)	319/759 = 42	N/A	111/150 = 74	N/A
	≥5 medications (FI)	156/496 = 31.4	N/A	274/413 = 66.3	N/A
(Jung., 2021)[253]	≥4 medications regularly	1162/2652 = 43.8	N/A	154/234 = 65.8	<0.001 (CS)
(Hoeksema 2017)[131]	4+ medications	252/584 = 43	N/A	163/225 = 72	N/A
(Jankowska-Polańska., 2016)[78]	5-9 medications	21/98 = 21.4	N/A	75/198 = 37.9	0.047 (CS)
(Tan., 2017)[145]	>5 medications	23/60 = 38.3	N/A	27/31 = 87.10	<0.001 (CS)

CS = Chi-Square Test; **MR** = Multinomial Logistic Regression; **BR** = Binomial Logistic Regression; **LRA** = Logistic Regression Analysis; **LRM** = Logistic Regression Model; **TT** = T-Tests; **QP** = Quasi-Poisson Model; KW= Kruskal Wallis test

OTC = Over-The-Counter Medications; **FP** = Frailty Phenotype; **AFI** = Adelaide Frailty Index; **FI** = Frailty Index; **EBC** = EMR Data (Frail) British Columbia; **ABC** = Admin Data (Frail) British Columbia; **BBC** = Both EMR & Admin (Frail) British Columbia; **EM** = EMR Data (Frail) Manitoba; **AM** = Admin Data (Frail) Manitoba; **BM** = Both EMR & Admin (Frail) Manitoba

Supplementary Table S7. Distribution of polypharmacy and hyper-polypharmacy based on frailty status

Study ID	Polypharmacy					Hyper-polypharmacy				
	**Defin-ition**	**NF (%)**	**PF (%)**	**F (%)**	**P-value**	**Defin-ition**	**NF (%)**	**PF (%)**	**F (%)**	**P-value**
Reallon 2020 [105]	5–9 /day	20/59 = 33.9	80/164 = 48.8	80/180 = 44.4	N/A	>9/day	9/59 = 15.3	23/164 = 14	37/180 = 20.6	N/A
Chaouacha., 2022[182]	5- 9	54/110 = 49.1	46/62 = 74.2	19/25 = 76	0.001 (CS)	>9	3/110 = 2.7	10/62 = 16.1	4/25 = 16	0.001 (CS)
Saum., 2017 [47]	5–9	324/1003 = 32.3	726/1784 = 40.6	144/271= 53.1	<0.001 (CS)	≥10	48/1003= 4.7	157/1784 = 8.8	68/271= 25	<0.001 (CS)
Lockery., 2020 [70]	5-9	2255/11246= 20	2213/7447 = 29.7	197/421 = 46.7	N/A	≥10	137/11246 = 1.2	251/7447= 3.3	35/421= 8.3	N/A
Gnjidic., 2012[83]	≥5	226/830 = 27.2	300/676 = 44.4	101/156 = 64.7	<0.0001 (LRM)	≥10	16/830 = 1.93	36/676 = 5.33	27/156 = 17.31	<0.0001 (LRM)
Houghton 2021[120]	≥5	N/A	N/A	N/A	N/A	≥10	19/92 = 21	N/A	34/98 = 35	0.031 (CS)
O'Connell 2020[124]	5-9	36/102 = 16.2	145/365 = 39.7	41/103 = 39.8	<0.001 (MR)	≥10	11/102 = 10.7	84/365 = 23	44/103 = 42.7	<0.001 (MR)
Serra-Prat., 2016[204]	6-10	33/104 = 32	76/174 = 45	30/46 = 65.2	<0.001 (CS)	>10	3/104 = 2.9	5/174 = 3.0	9/46 = 19.6	<0.001 (CS)
Koponen., 2013[208]	6-9	66/237 = 27.8	117/299 = 39.1	26/69 = 37.7	<0.001 (CS)	≥10	12/237 = 5.1	46/299 = 15.4	28/69 = 40.6	<0.001 (CS)
Porter., 2019[211]	5–9	56/204 = 27.5	229/530 = 43.2	208/420 = 49.5	<0.01 (CS)	≥10	5/204 = 2.5	41/530 = 7.7	64/420 = 15.2	<0.01 (CS)
Herr., 2015[217]	5–9	265/654 = 22.6	523/893 = 58.5	209/388= 53.8	<0.001 (MP)	≥10	27/654 = 4.1	97/893 = 10.8	85/388 = 21.9	<0.001 (MP)

CS = Chi-Square Test; LRM = Logistic Regression Model; MR = Multinomial Logistic Regression; MP = Multivariate Polytomous Regression
